# Supplementary figures and images for: Identification and characterization of distinct brown adipocyte subtypes in C57BL/6J mice
Source: Life Sci Alliance. 2020 Nov 30;4(1):e202000924. doi: 10.26508/lsa.202000924 (PMC7723269; doi:10.26508/lsa.202000924)

Merged

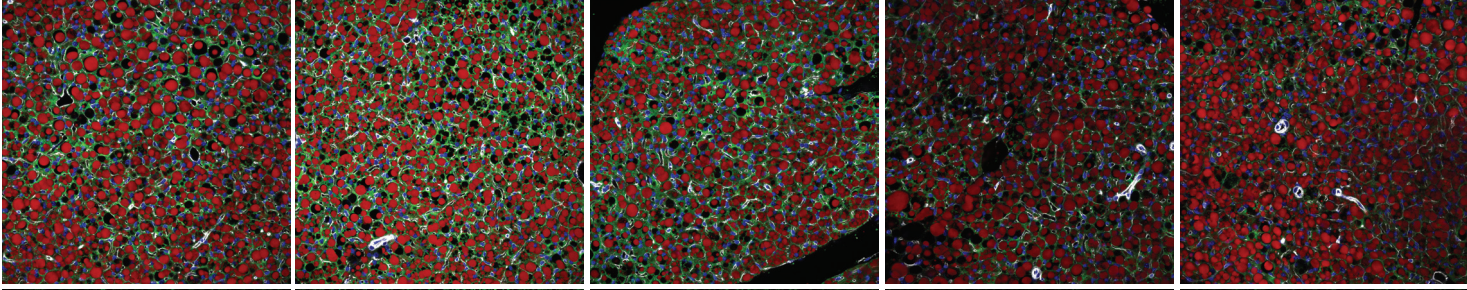

UCP1

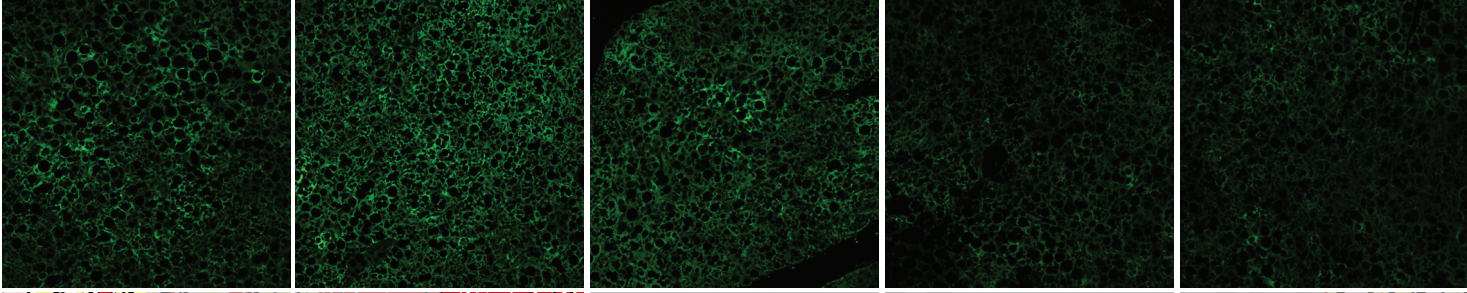

Quantification

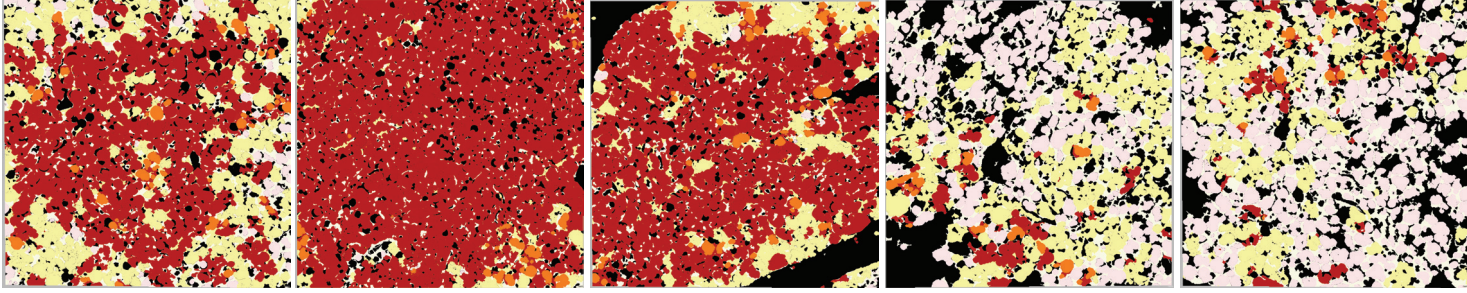

Merged

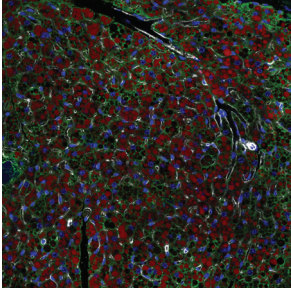

UCP1

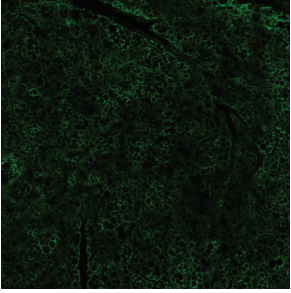

Quantification

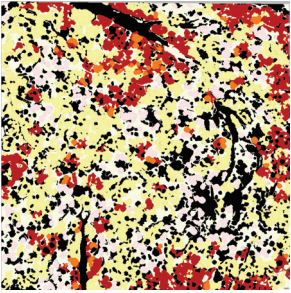

Supplement: Supplementary file 2 [file LSA-2020-00924_SdataF1.1.pdf]

Merged

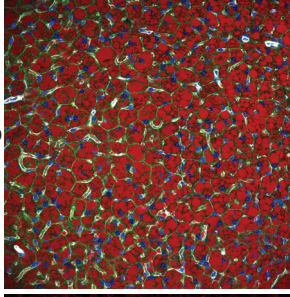

EIF5

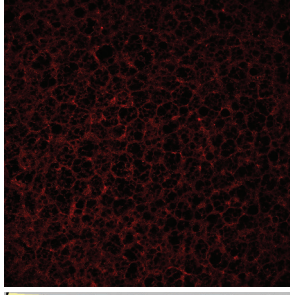

Quantification

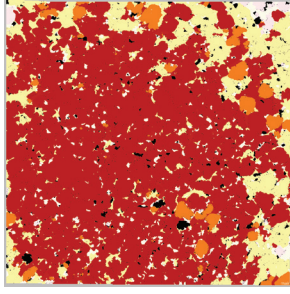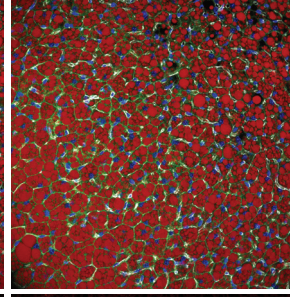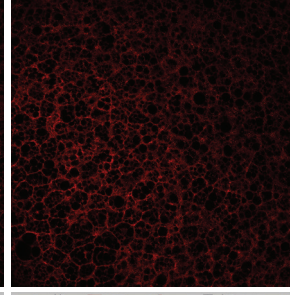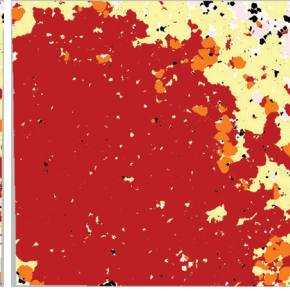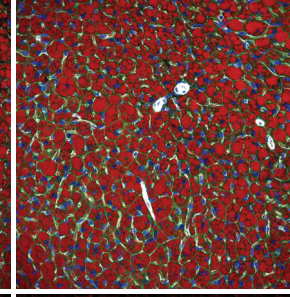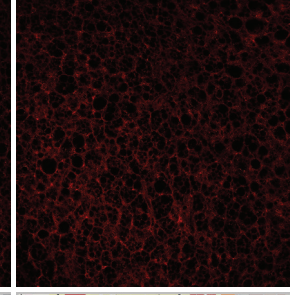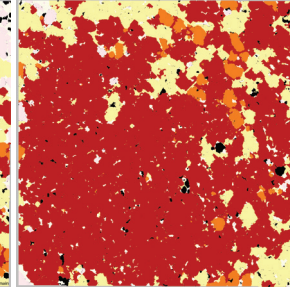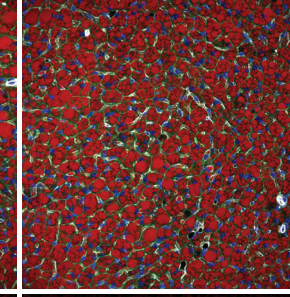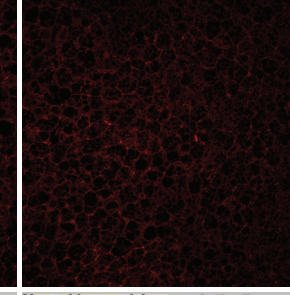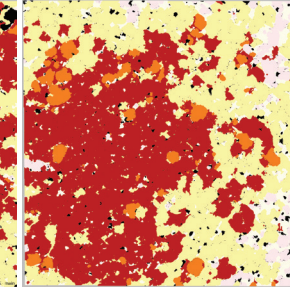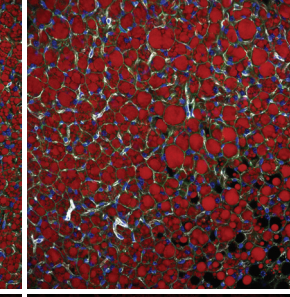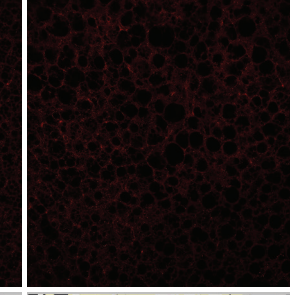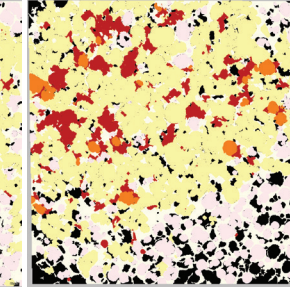

Merged

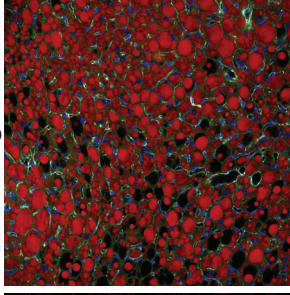

EIF5

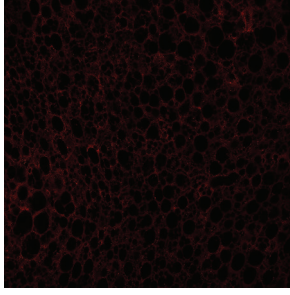

Quantification

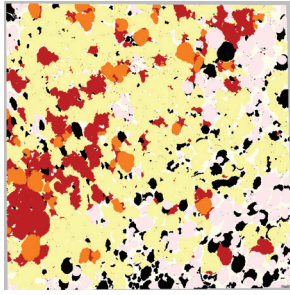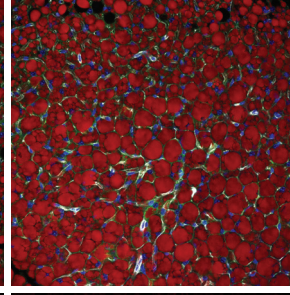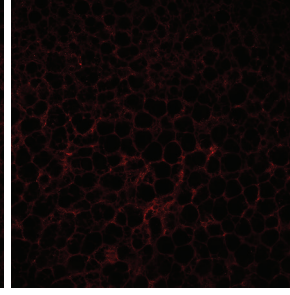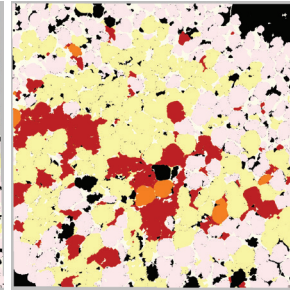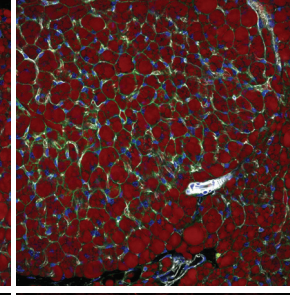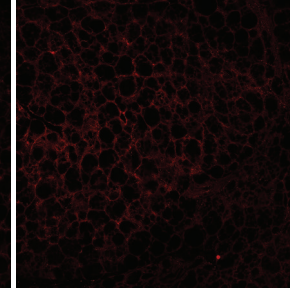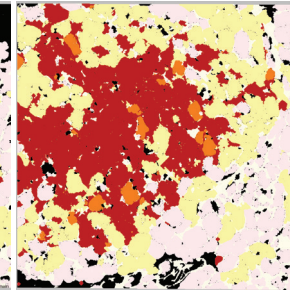

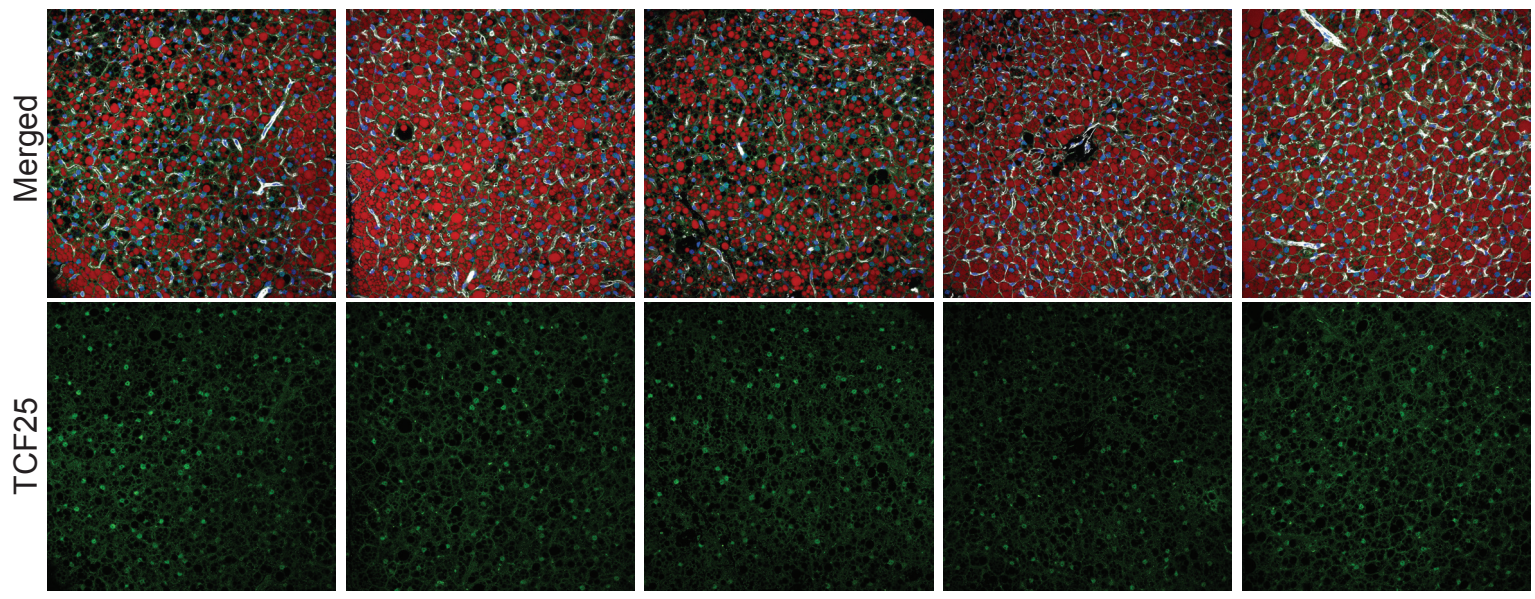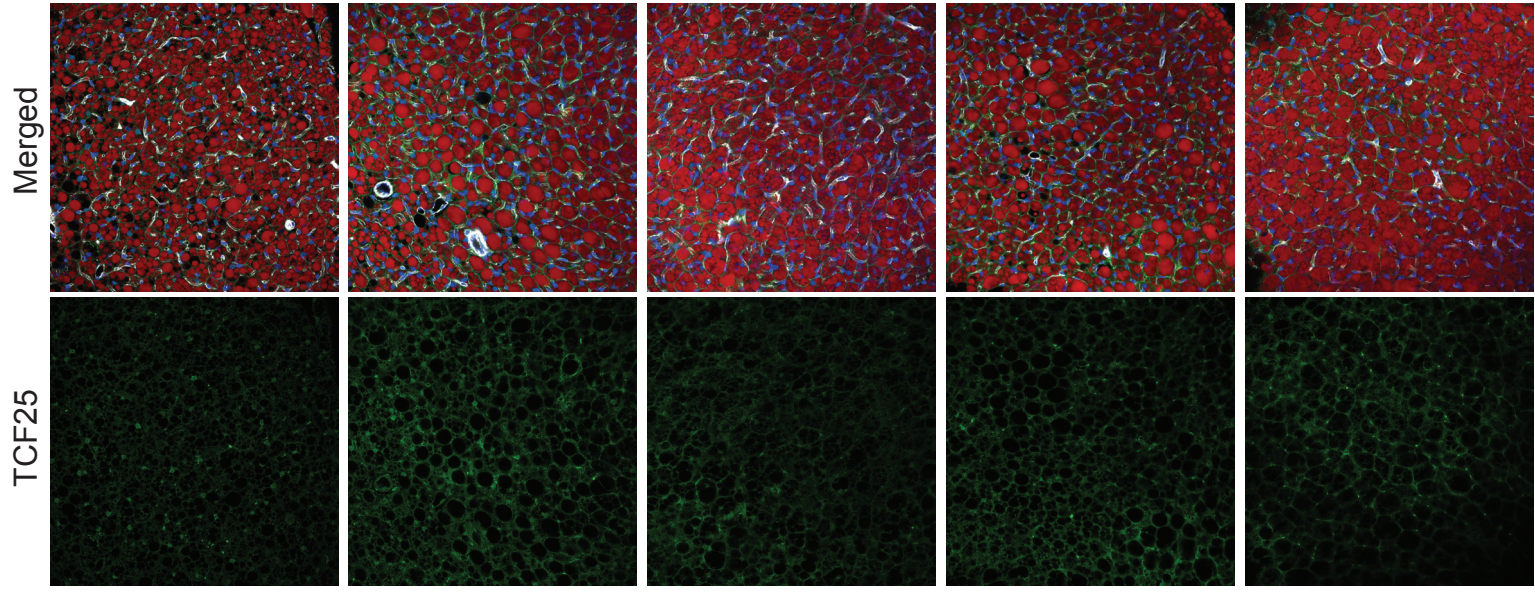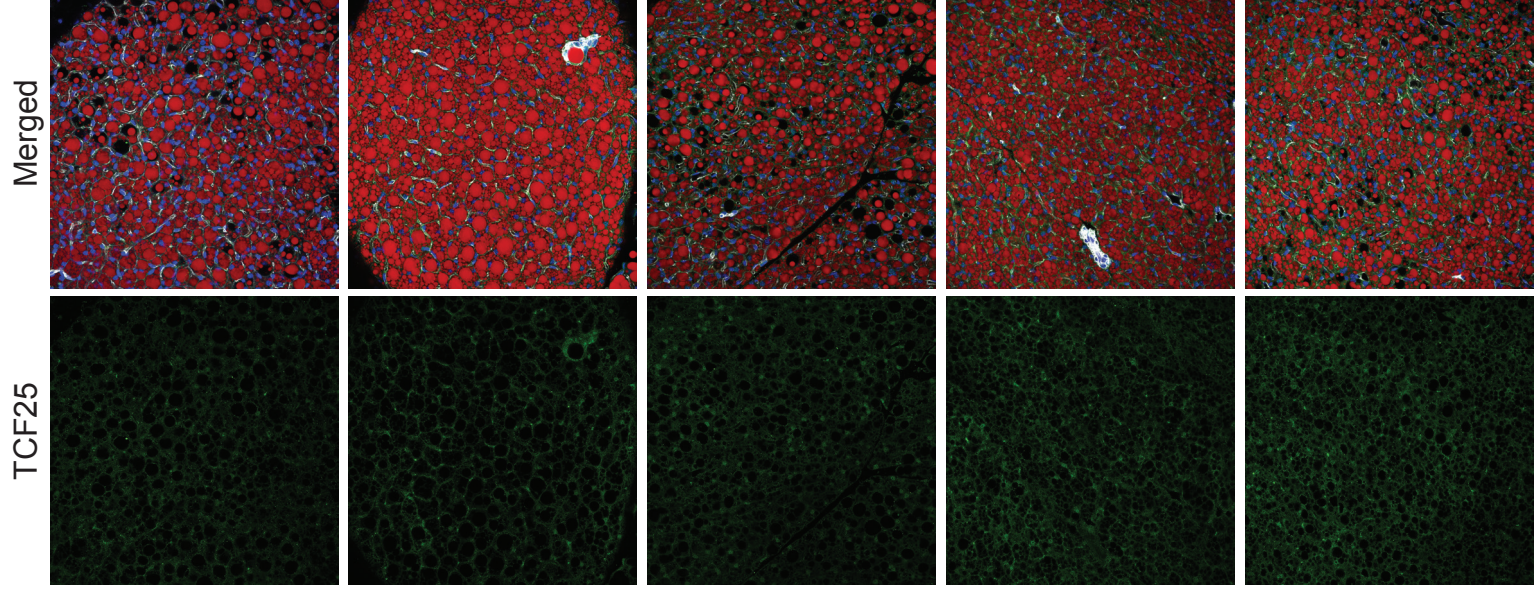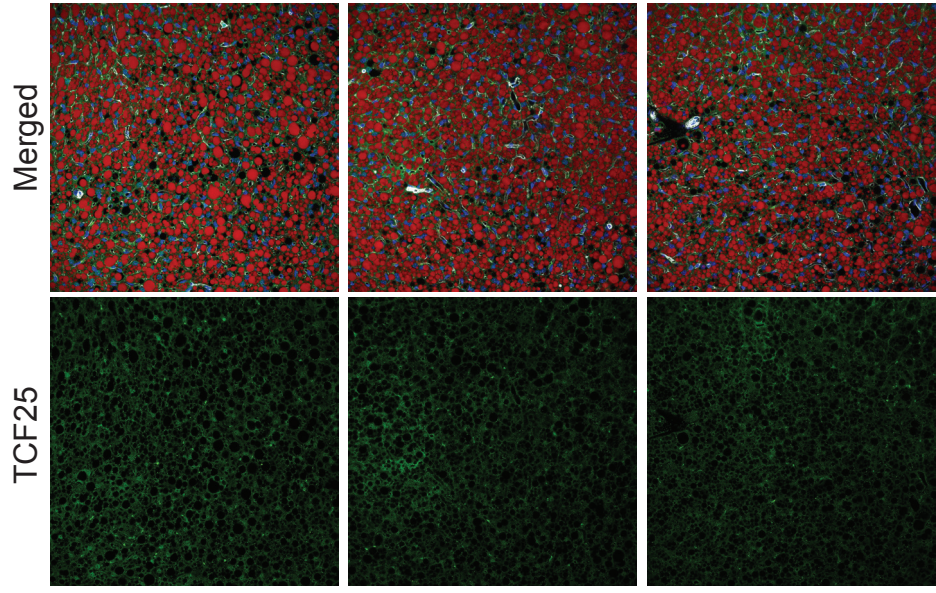

Merged

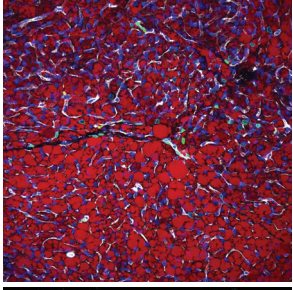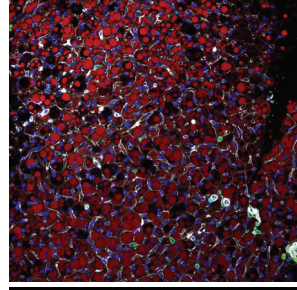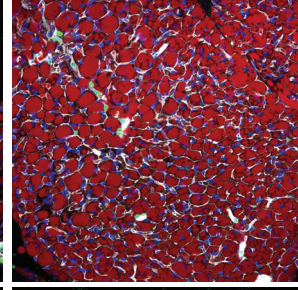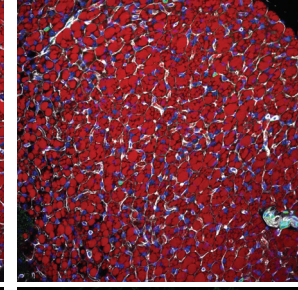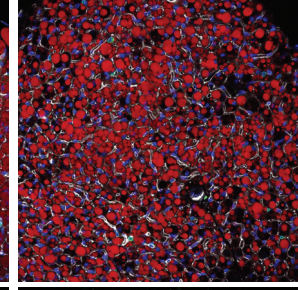

BIN1

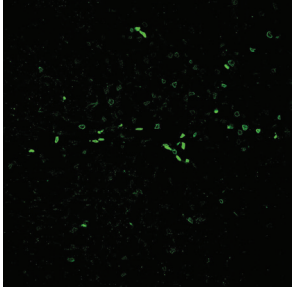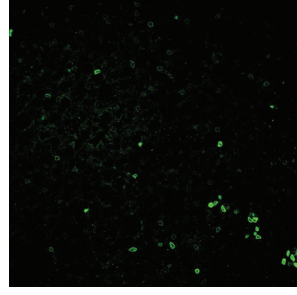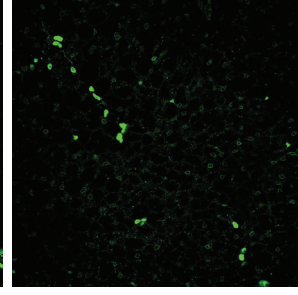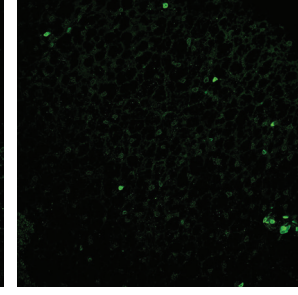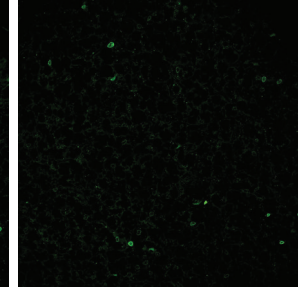

Merged

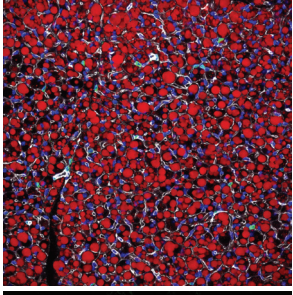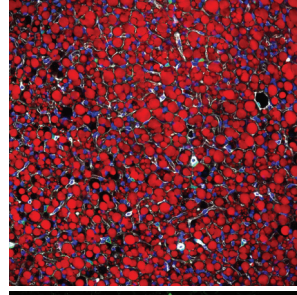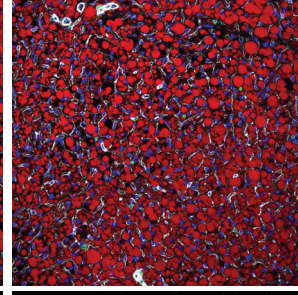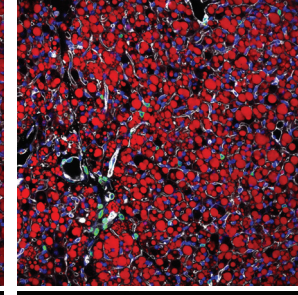

BIN1

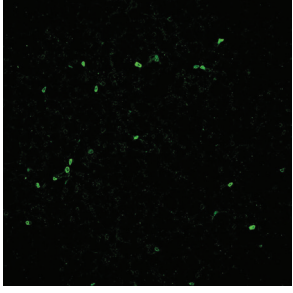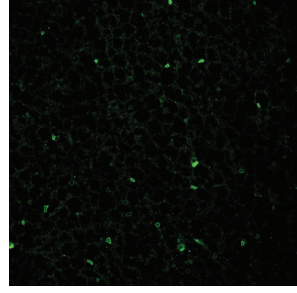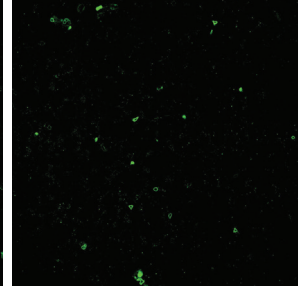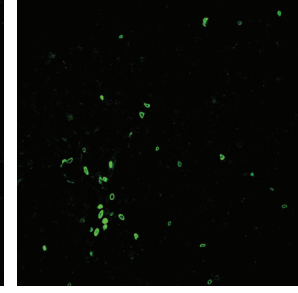

Supplement: Supplementary file 16 [file LSA-2020-00924_SdataF6.2.pdf]

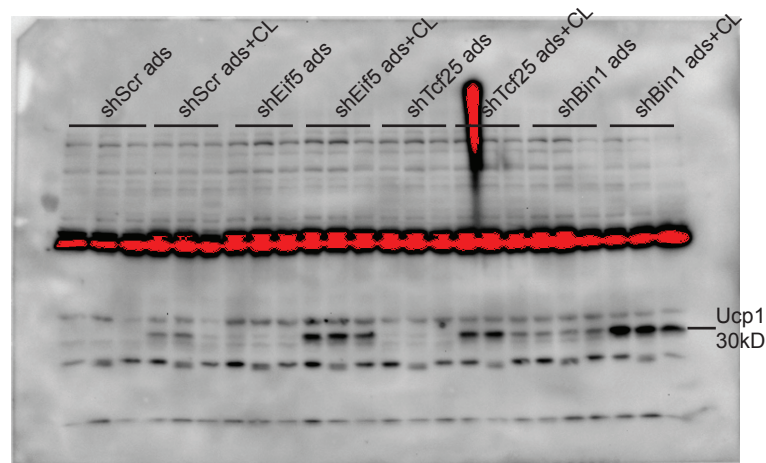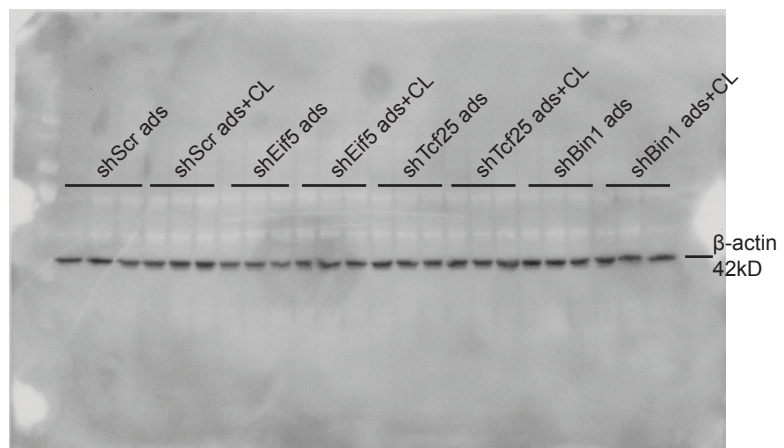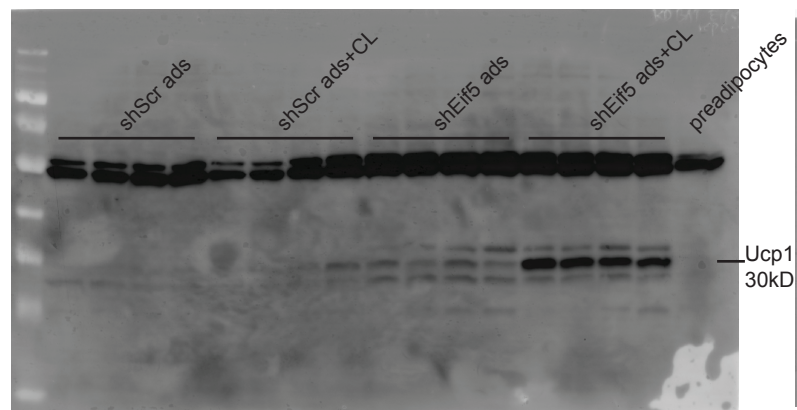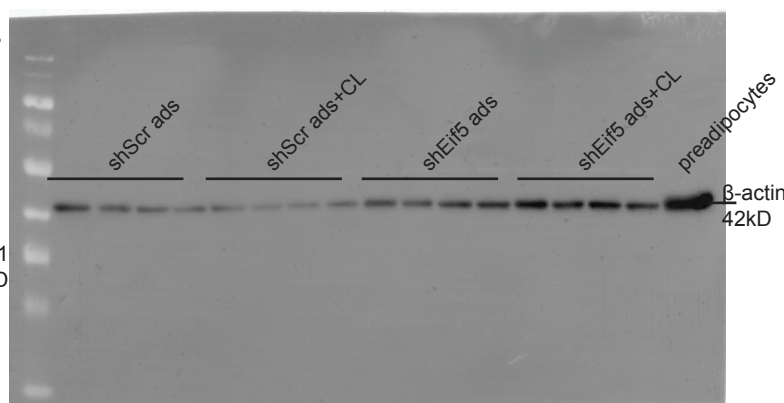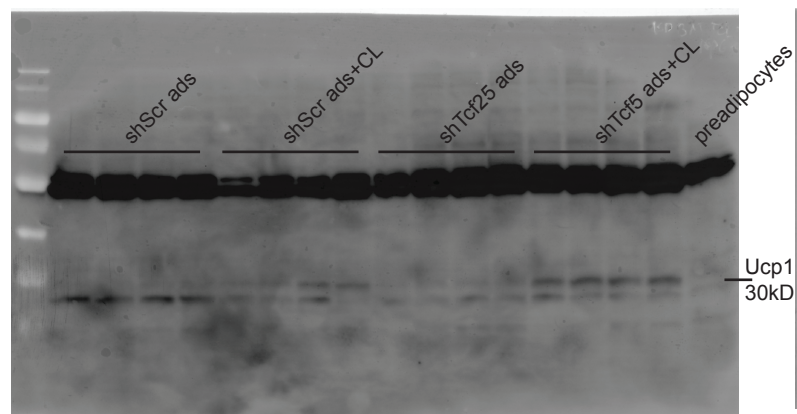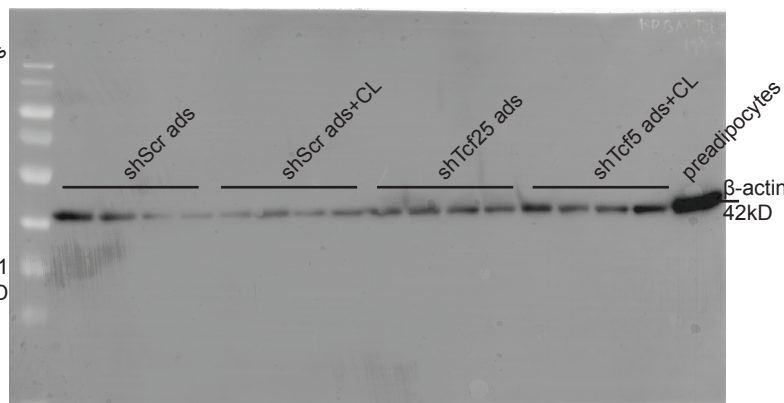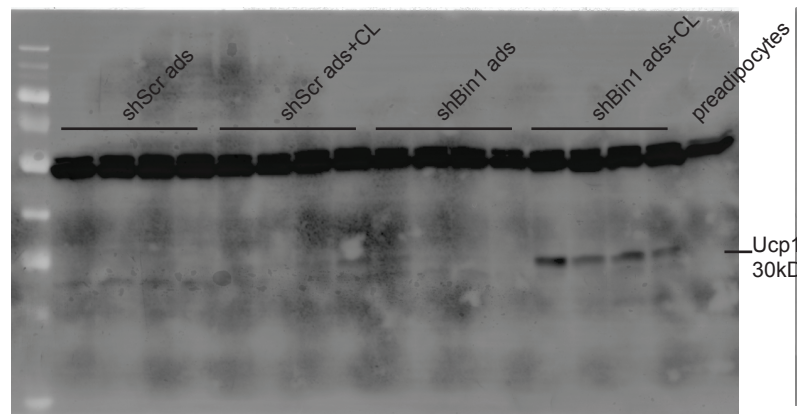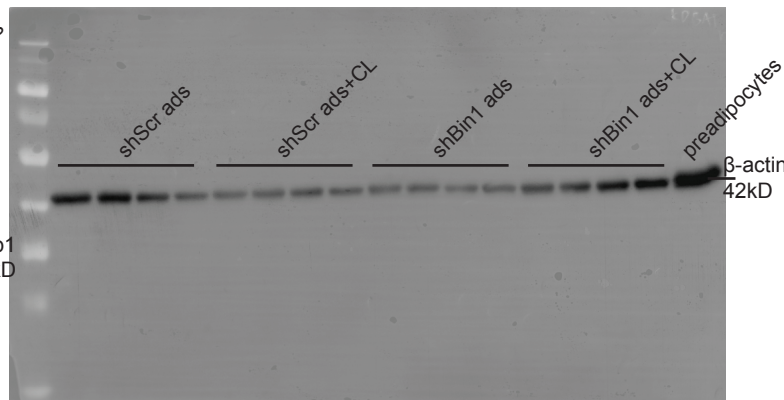

Supplement: Supplementary file 21 [file LSA-2020-00924_SdataF7.3.pdf]
