# Supplementary material for: Identification and characterization of distinct brown adipocyte subtypes in C57BL/6J mice
Source: Life Sci Alliance. 2020 Nov 30;4(1):e202000924. doi: 10.26508/lsa.202000924 (PMC7723269; doi:10.26508/lsa.202000924)

Analysis of Mouse Adipose Data Set

Table of contents:

- Load Packages and Set Global Variables
  - Imports and Settings
  - Global Variables
- Loading Data, Quality Control and Preprocessing
- Define Cell Types
- Adipocytes Only
  - Embeddings and Clustering
  - Adipocyte Marker Analysis
  - Top ranking DE Genes
  - Count distribution for Tcf25, Bin1 and Eif5
- Preadipocytes only

Load Packages and Set Global Variables

Imports and Settings

In [1]:
```
import numpy as np
import scanpy.api as sc
import sys
import pandas as pd
import seaborn as sb
import matplotlib.pyplot as plt
```

```
/Users/david.fischer/opt/miniconda3/lib/python3.7/site-packages/an
ndata/_core/anndata.py:21: FutureWarning: pandas.core.index is dep
recated and will be removed in a future version.  The public class
es are available in the top-level namespace.
  from pandas.core.index import RangeIndex
/Users/david.fischer/opt/miniconda3/lib/python3.7/site-packages/sc
anpy/api/__init__.py:6: FutureWarning:

In a future version of Scanpy, `scanpy.api` will be removed.
Simply use `import scanpy as sc` and `import scanpy.external as sc
e` instead.

  FutureWarning,
```

In [2]:
```
base_dir = '/Users/david.fischer/phd/datasets/2019_Ussar_adipocytes
/MUC8387/'
dir_out = '/Users/david.fischer/phd/data/PreadipocytesBrown/results
/'

sc.settings.verbosity = 3 # amount of output
dir_in = base_dir+'mm10_ensrel94/'
dir_tables = dir_out+'tables/'
sc.settings.figdir = dir_out+'panels/'
dir_adata = dir_out+'anndata/'
sc.logging.print_versions()
sc.settings.set_figure_params(dpi=80, scanpy=True)
print (sys.version)
```

```
scanpy==1.4.5.1 anndata==0.7.1 umap==0.3.10 numpy==1.18.1 scipy==1
.4.1 pandas==1.0.1 scikit-learn==0.22.2.post1 statsmodels==0.11.1
python-igraph==0.8.0 louvain==0.6.1
3.7.4 (default, Aug 13 2019, 15:17:50)
[Clang 4.0.1 (tags/RELEASE_401/final)]
```

Global Variables

All embeddings and clusterings can be saved and loaded into this script. Be carful with overwriting cluster caches as soon as cell type annotation has started as cluster labels may be shuffled.

Set whether anndata objects are recomputed or loaded from cache.

In [3]:
```
bool_recomp = False
```

Set whether clustering is recomputed or loaded from saved .obs file. Loading makes sense if the clustering changes due to a change in scanpy or one of its dependencies and the number of clusters or the cluster labels change accordingly.

In [4]:
```
bool_recluster = False
```

Set whether cluster cache is overwritten. Note that the cache exists for reproducibility of clustering, see above.

In [5]:
```
bool_write_cluster_cache = False
```

Set whether to produce plots, set to False for test runs.

In [6]:
```
bool_plot = True
```

Set whether observations should be calculated. If false, it is necessary to read cacheed file that contains the necssary information. It then shows the the distributions of counts and genes, as well as mt_frac after filtering. Set to true in order to see the data before filtering and follow the decisions for cutoffs.

In [7]:
```
bool_create_observations = True
```

Loading Data, Quality Control and Preprocessing

Read the data in:

In [8]:
```
adata_raw = sc.read(dir_in+'raw_gene_bc_matrices.h5ad')
```

Quality Control

Summary of steps performed here: Only cells with at least 500 UMIs are kept. Counts per cell are cell library depth normalized. The gene (feature) space is reduced with PCA to 50 PCs. A nearest neighbour graph and umap are computed based on the PC space. Cell are clustered with louvain clustering based on the nearest neighbour graph. Graph abstraction is computed based on the louvain clustering.

In [9]:
```
adata_raw.shape
```

Out[9]:
```
(737280, 31125)
```

The data contains 737280 observations with 31125 different genes. Due to dropouts, some of the observations might not show any counts and genes. In order to calculate the fraction of mitochondrial RNA in the next steps, each observations without counts must be filtered out to prevent NaN from emerging.

In [10]:
```
print('Total number of cells: {:d}'.format(adata_raw.n_obs))

sc.pp.filter_cells(adata_raw, min_counts = 1)
print('Number of cells after min count filter: {:d}'.format(adata_r
aw.n_obs))

adata_raw.shape
```

```
Total number of cells: 737280
filtered out 352632 cells that have less than 1 counts
Number of cells after min count filter: 384648
```

Out[10]:
```
(384648, 31125)
```

From the 737280 observations, only 384648 are left with counts greater than 0, which is ~52%.

Gene numbers and counts with and without mitochondrial RNA

Create necessary obs:

In [11]:
```
adata_qc = adata_raw.copy()
adata_qc.obs['n_genes'] = (adata_qc.X > 0).sum(1)
mt_gene_mask = [gene.startswith('mt-') for gene in adata_qc.var_nam
es]
temp_mt_sum = adata_qc[:,mt_gene_mask].X.sum(1)
temp_mt_sum = np.squeeze(np.asarray(temp_mt_sum))
temp_n_counts = adata_qc.obs['n_counts']
adata_qc.obs['mt_frac'] = temp_mt_sum/adata_qc.obs['n_counts']
```

Plot n_counts and mt_frac:

In [12]:
```
if bool_plot == True:
    t1 = sc.pl.violin(adata_qc, 'n_counts', size =2 ,log=True, cut
= 0)
    t2 = sc.pl.violin(adata_qc, 'mt_frac')
```

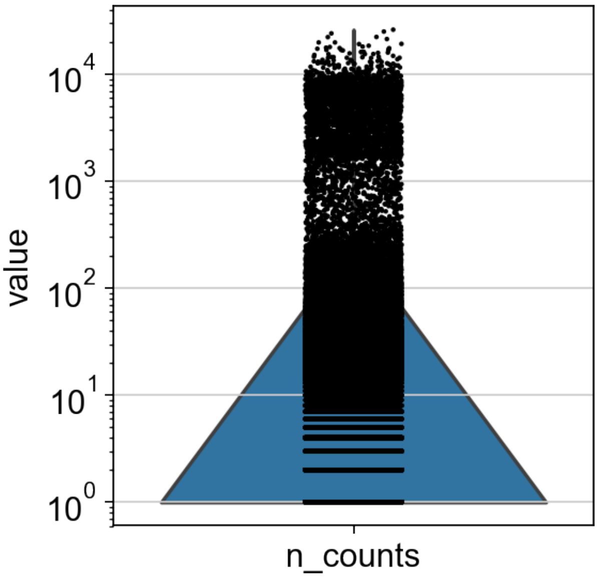

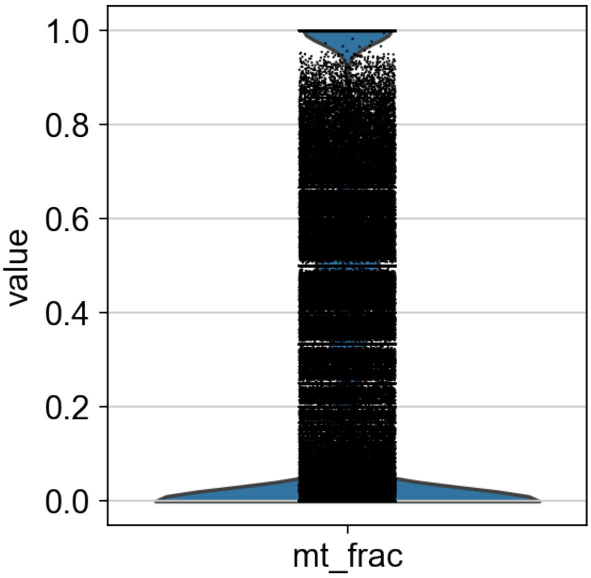

Overall, the data contains a lot of observations with high fractions of mitochondrial RNA. Additionally, most observations show counts below 100, suggesting poor data quality. To further investigate the distributions counts over genes per observations, scatterplots are created:

Number of Genes versus Number of Counts

In [13]:
```
if bool_plot == True:
    p1 = sc.pl.scatter(adata_qc, 'n_counts','n_genes',color='mt_frac', size = 5)
    p2 = sc.pl.scatter(adata_qc[adata_qc.obs['n_counts']<10000],'n_counts','n_genes',color = 'mt_frac', size = 5)
    p3 = sc.pl.scatter(adata_qc[adata_qc.obs['n_counts']<1000],'n_counts','n_genes',color = 'mt_frac', size = 5)
```

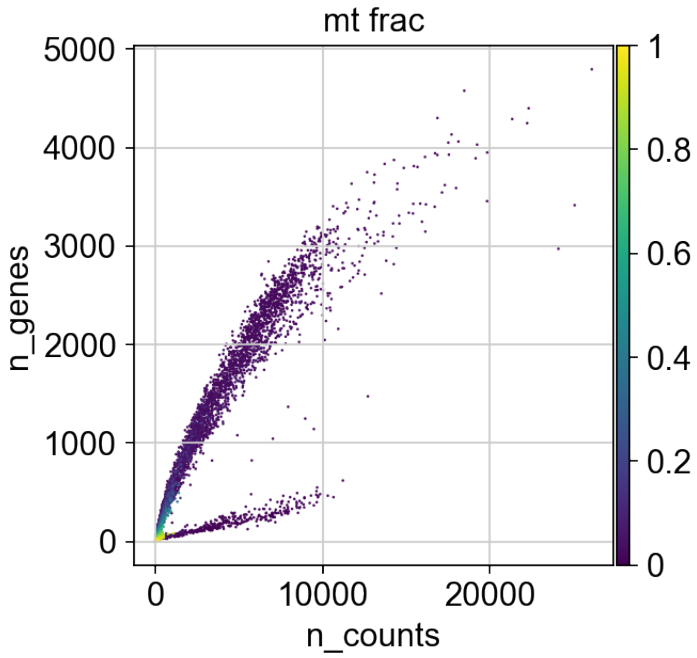

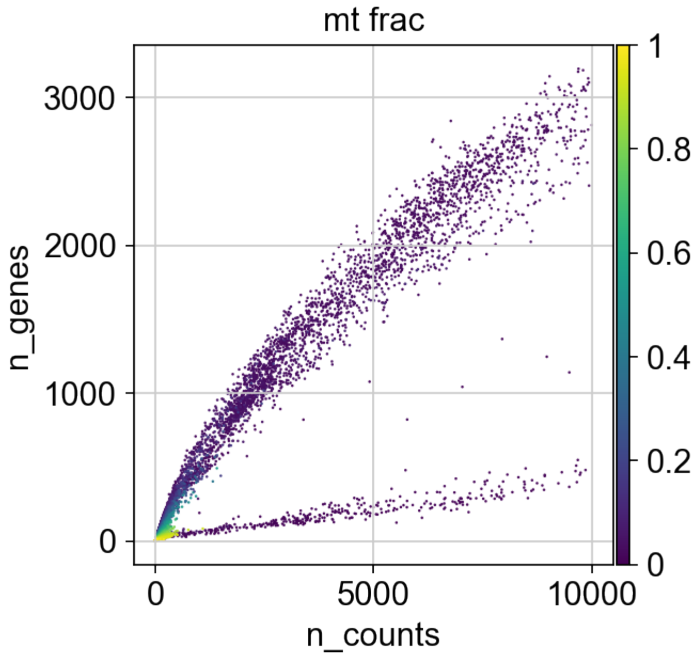

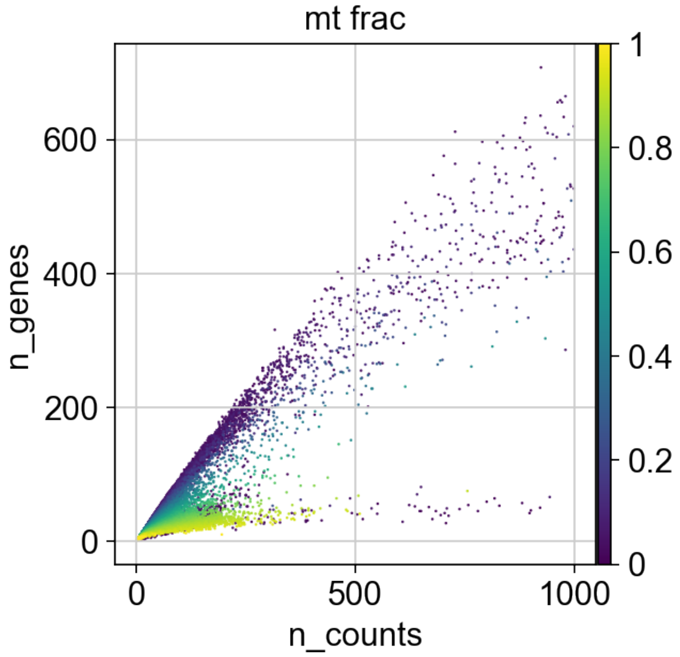

Distribution of Counts and Genes

In [14]:
```
if bool_plot == True:
    p6 = sb.distplot(adata_qc.obs['n_counts'], kde = False)
    plt.show()
    p7 = sb.distplot(adata_qc.obs['n_counts'][adata_qc.obs['n_count
s']<1000], kde = False)
    plt.show()
    p8 = sb.distplot(adata_qc.obs['n_counts'][adata_qc.obs['n_count
s']<100], kde = False)
    plt.show()
```

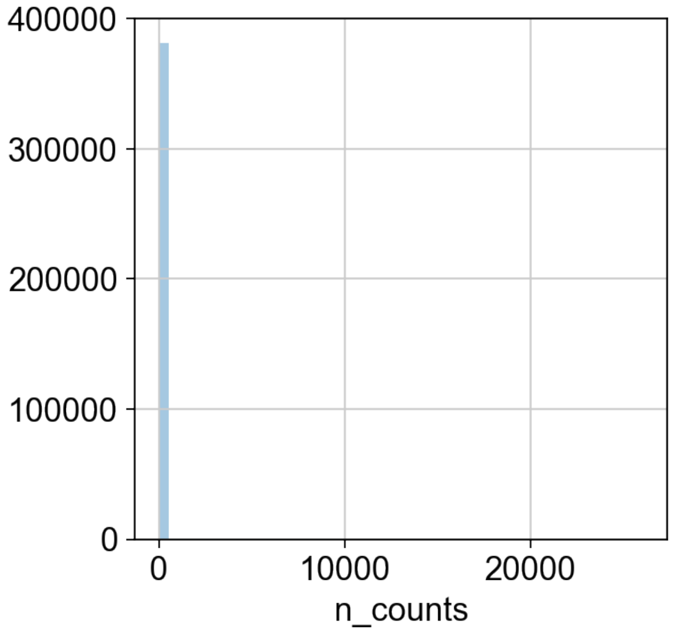
400000
300000
200000
100000
0
0
10000
20000
n_counts

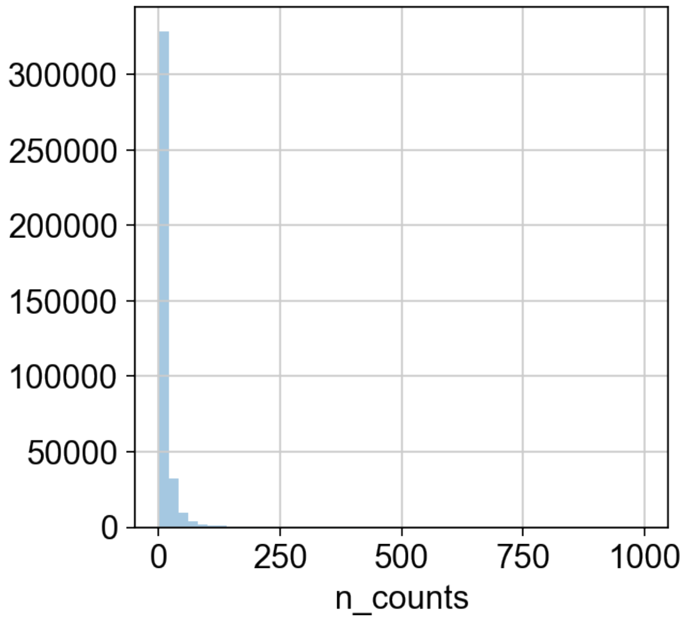
300000
250000
200000
150000
100000
50000
0
0
250
500
750
1000
n_counts

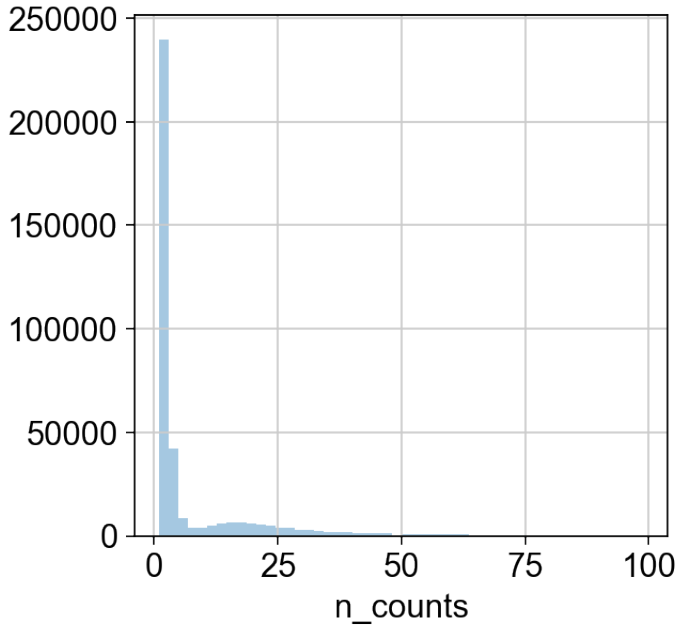

In [15]:
```
if bool_plot == True:
    p9 = sb.distplot(adata_qc.obs['n_genes'],kde = False, bins=60)
    plt.show()
    p10 = sb.distplot(adata_qc.obs['n_genes'][adata_qc.obs['n_genes
']<100],kde = False, bins=60)
    plt.show()
```

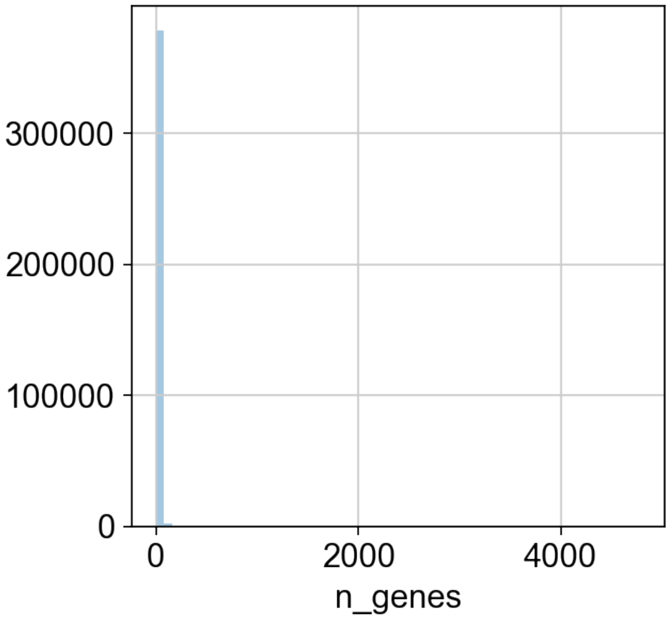
300000
200000
100000
0
0
2000
4000
n_genes

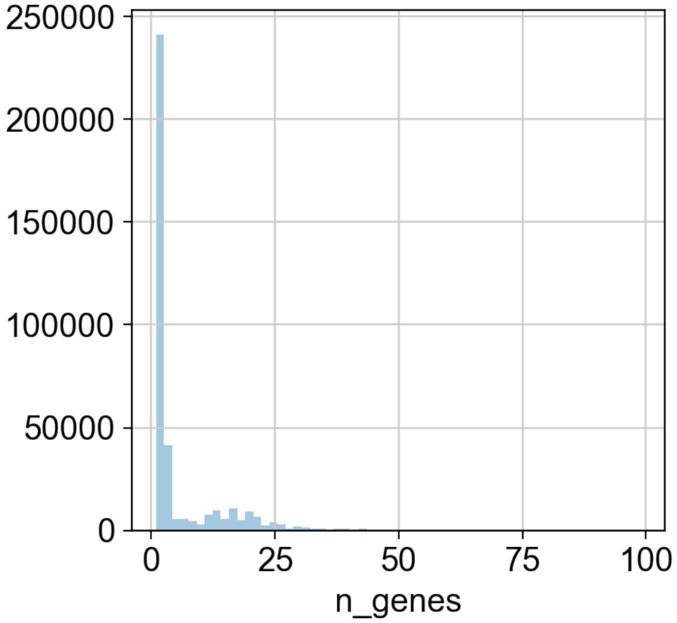
250000
200000
150000
100000
50000
0
0
25
50
75
100
n_genes

Filtering

In [16]:
```
# Filter cells according to identified QC thresholds:
print('Total number of cells: {:d}'.format(adata_qc.n_obs))

sc.pp.filter_cells(adata_qc, min_counts = 200)
print('Number of cells after min count filter: {:d}'.format(adata_q
c.n_obs))

sc.pp.filter_cells(adata_qc, max_counts = 15000)
print('Number of cells after max count filter: {:d}'.format(adata_q
c.n_obs))

adata_qc = adata_qc[adata_qc.obs['mt_frac'] < 0.2]
print('Number of cells after MT filter: {:d}'.format(adata_qc.n_obs
))

sc.pp.filter_cells(adata_qc, min_genes = 100)
print('Number of cells after gene filter: {:d}'.format(adata_qc.n_o
bs))
```

```
Total number of cells: 384648
filtered out 380040 cells that have less than 200 counts
Number of cells after min count filter: 4608
filtered out 32 cells that have more than 15000 counts
Number of cells after max count filter: 4576
Number of cells after MT filter: 4163
filtered out 150 cells that have less than 100 genes expressed

Trying to set attribute `.obs` of view, copying.

Number of cells after gene filter: 4013
```

Only 4013 observations are left that pass the filters.

In [17]:
```
if bool_plot == True:
    p12 = sc.pl.scatter(adata_qc, 'n_counts','n_genes',color='mt_fr
ac', size = 5)
    p22 = sc.pl.scatter(adata_qc[adata_qc.obs['n_counts']<10000],'n
_counts','n_genes',color = 'mt_frac', size = 5)
    p52 = sc.pl.scatter(adata_qc[adata_qc.obs['n_counts']<1000],'n_
counts','n_genes',color = 'mt_frac', size = 5)
```

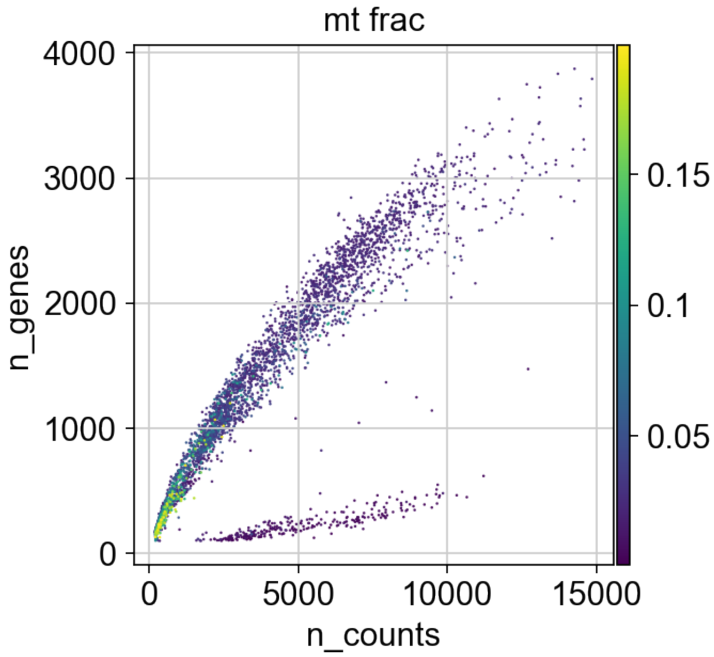
mt frac
4000
3000
2000
1000
0
n_genes
0
5000
10000
15000
n_counts
0.15
0.1
0.05

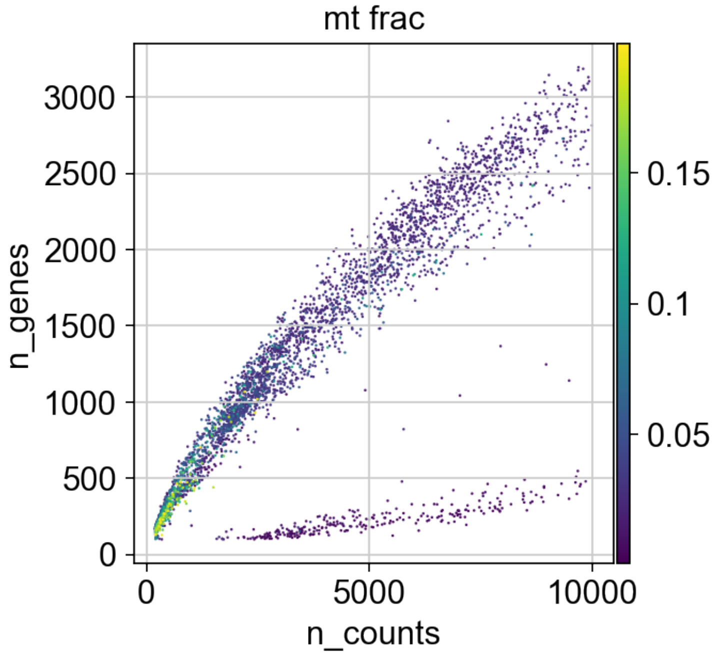
mt frac
3000
2500
2000
1500
1000
500
0
n_genes
0
5000
10000
n_counts
0.15
0.1
0.05

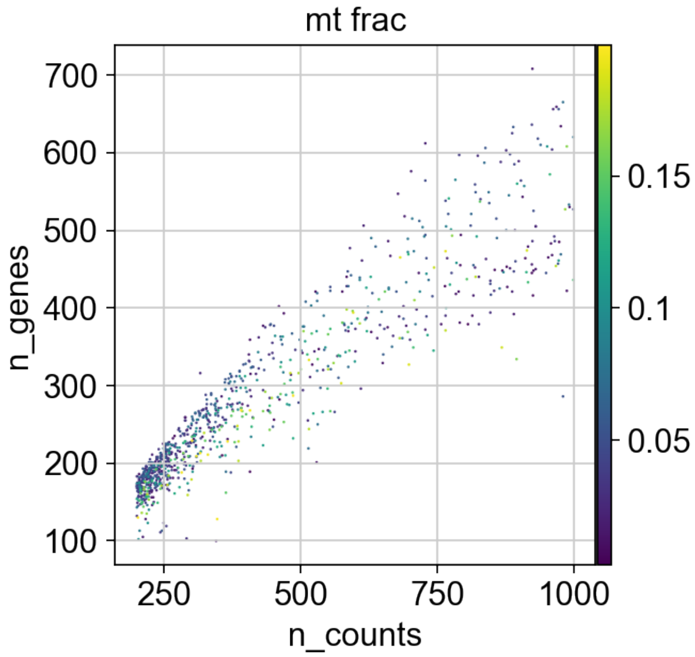

In [18]:
```
if bool_plot == True:
    sc.pl.violin(adata_qc, 'n_counts', size=2, log=True, cut=0)
    sc.pl.violin(adata_qc,'mt_frac')
```

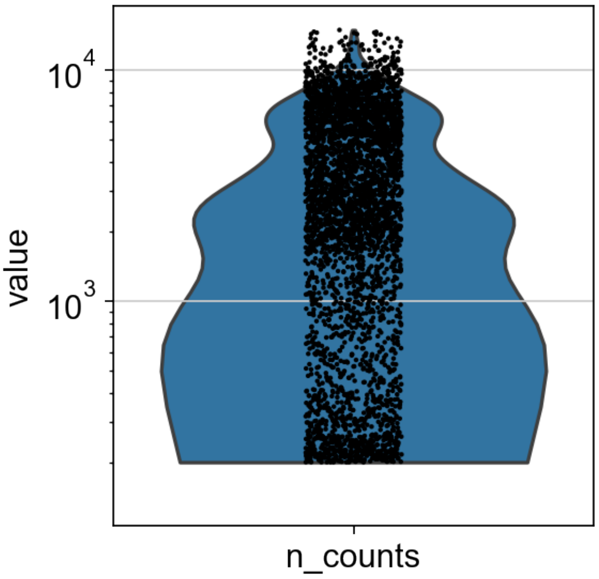

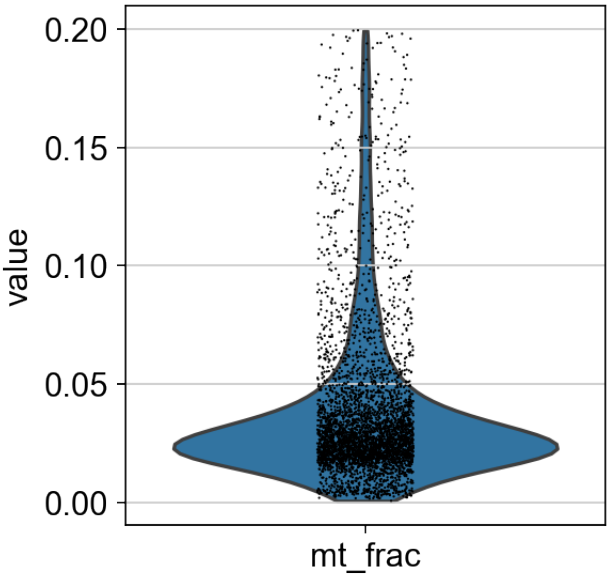

For the remaining observations, the fraction of mitochondrial RNA is generally very low and at most 20%

In [19]:
```
if bool_plot == True:
    p62 = sb.distplot(adata_qc.obs['n_counts'], kde = False)
    plt.show()
    p72 = sb.distplot(adata_qc.obs['n_counts'][adata_qc.obs['n_coun
ts']<1000], kde = False)
    plt.show()
```

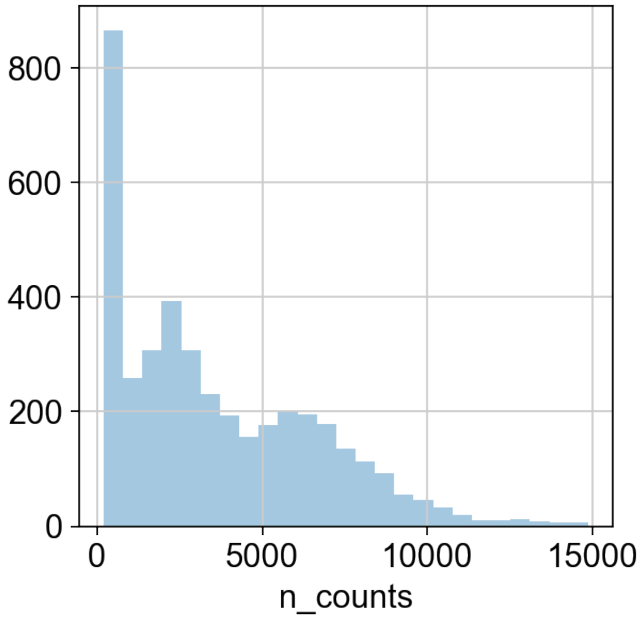

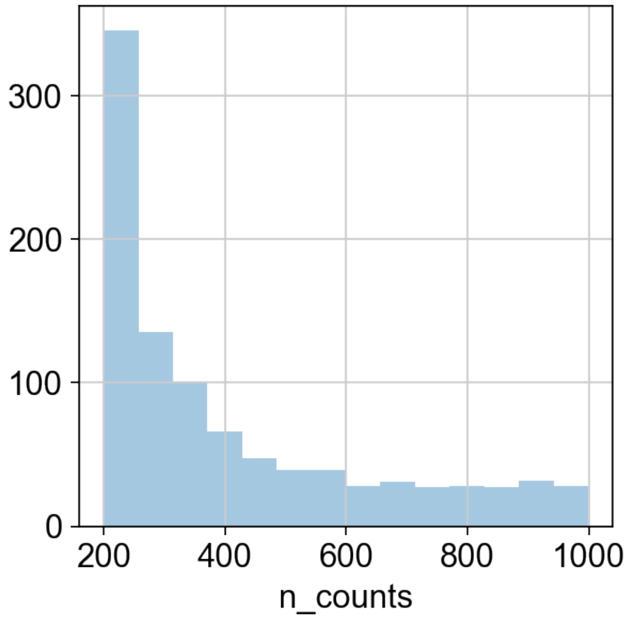

In [20]:
```
if bool_plot == True:
    p92= sb.distplot(adata_qc.obs['n_genes'],kde = False, bins=60)
    plt.show()
    p100 = sb.distplot(adata_qc.obs['n_genes'][adata_qc.obs['n_gene
s']<1000],kde = False, bins=60)
    plt.show()
```

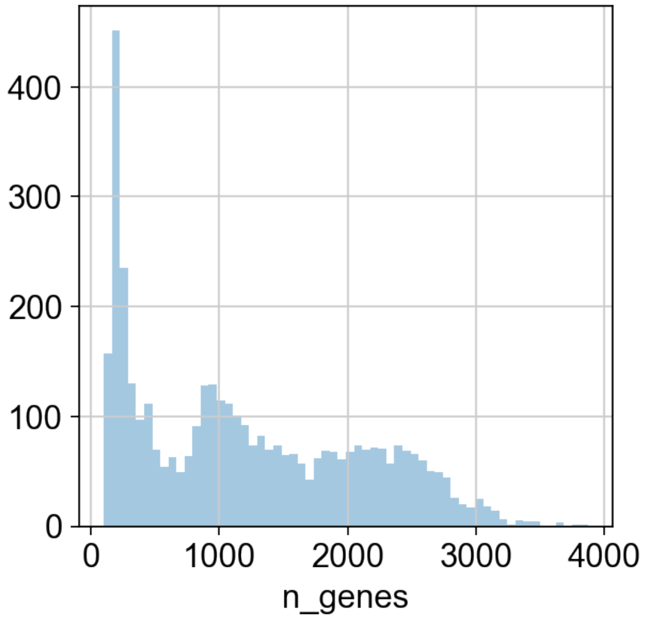

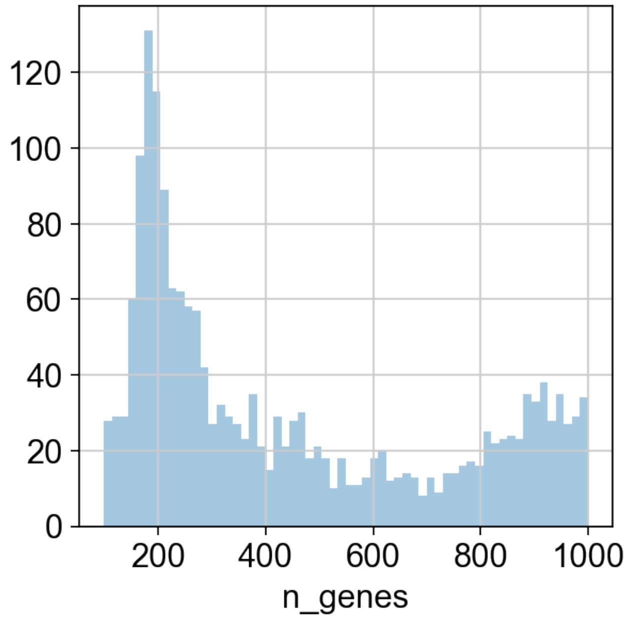

In [21]:
```
#Filter genes:
print('Total number of genes: {:d}'.format(adata_qc.n_vars))

# Min 20 cells - filters out 0 count genes
sc.pp.filter_genes(adata_qc, min_cells=20)
print('Number of genes after cell filter: {:d}'.format(adata_qc.n_v
ars))
```

```
Total number of genes: 31125
filtered out 18501 genes that are detected in less than 20 cells
Number of genes after cell filter: 12624
```

Normalization and Clustering with highly variable genes

In [22]:
```
if bool_recomp == True:

    adata_proc = adata_qc.copy()
    adata_proc.raw = adata_proc
    sc.pp.normalize_per_cell(adata_proc)
    sc.pp.log1p(adata_proc)
    sc.pp.highly_variable_genes(adata_proc, flavor='cell_ranger',n_
top_genes=4000)
    sc.pl.highly_variable_genes(adata_proc)

    sc.pp.pca(adata_proc, n_comps=50, random_state=0, use_highly_va
riable=True, svd_solver='arpack')
    sc.pp.neighbors(adata_proc, n_neighbors=100, knn=True, method='
umap', n_pcs=50, random_state=0)
    #sc.tl.tsne(adata_proc, n_jobs=3)
    sc.tl.umap(adata_proc)
    if bool_recluster == True:
        sc.tl.louvain(adata_proc, resolution=1, flavor='vtraag', ra
ndom_state=0)
        pd.DataFrame(adata_proc.obs).to_csv(path_or_buf =dir_adata+
"obs_adata_proc.csv")
    else:
        obs = pd.read_csv(dir_adata+'obs_adata_proc.csv')
        adata_proc.obs['louvain']=pd.Series(obs['louvain'].values,
dtype = 'category')
    sc.write(dir_adata+'adata_proc.h5ad',adata_proc)
else:
    adata_proc = sc.read(dir_adata+'adata_proc.h5ad')
sc.tl.paga(adata_proc)
```

```
running PAGA
    finished: added
    'paga/connectivities', connectivities adjacency (adata.uns)
    'paga/connectivities_tree', connectivities subtree (adata.uns)
(0:00:00)
```

Produce some summarizing plots that show the global characteristics of the data.

In [23]:
```
if bool_plot == True:
    sc.pl.umap(adata_proc, color=['louvain','mt_frac'], size=5, sav
e="_all_louvain.pdf", use_raw = False)
```

```
WARNING: saving figure to file /Users/david.fischer/phd/data/Pread
ipocytesBrown/results/panels/umap_all_louvain.pdf
```

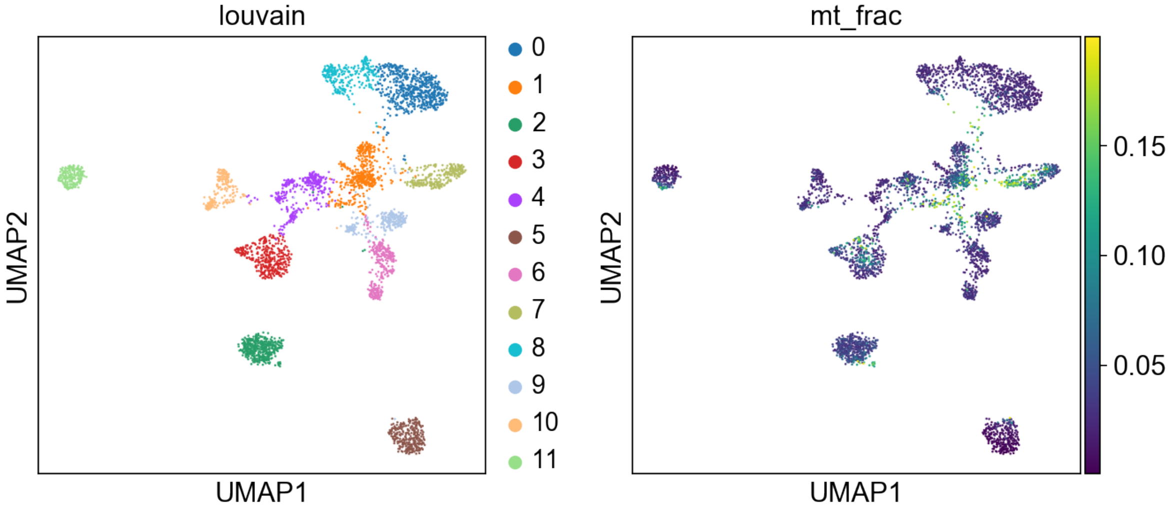

A high fraction of mitochondrial RNA is in cluster 7 and around the central cluster 2.

In [24]:
```
if bool_plot == True:
    sc.pl.umap(adata_proc, color=['n_genes','n_counts'], size=5, sa
ve="_all_n_counts.pdf", use_raw = False)
```

```
WARNING: saving figure to file /Users/david.fischer/phd/data/Pread
ipocytesBrown/results/panels/umap_all_n_counts.pdf
```

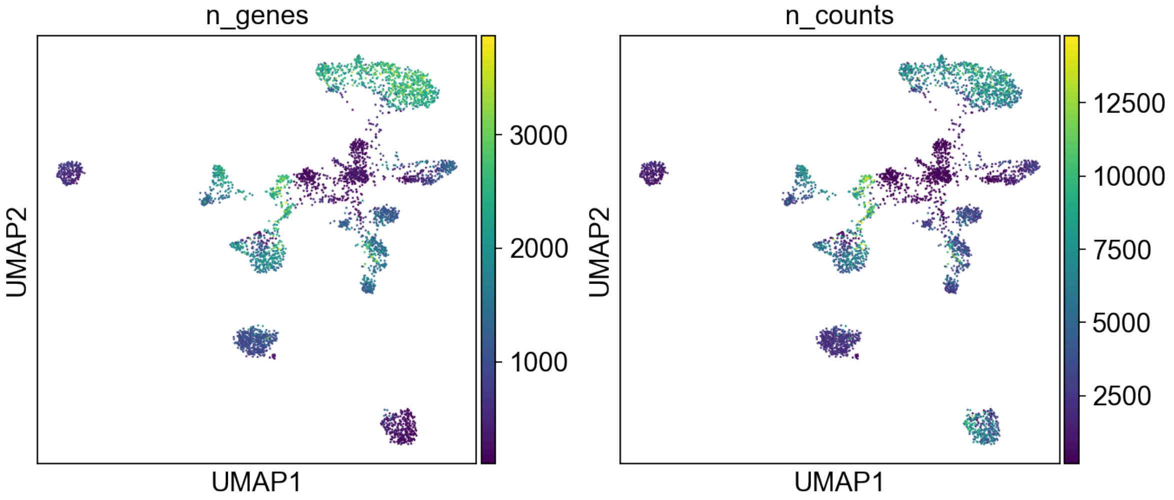

Given the number of genes and number of counts, cluster 0 and 8 could contain preadipocytes.

In [25]:
```
if bool_plot == True:
    sc.pl.paga(adata_proc, save="_all.pdf")
```

```
--> added 'pos', the PAGA positions (adata.uns['paga'])
WARNING: saving figure to file /Users/david.fischer/phd/data/Pread
ipocytesBrown/results/panels/paga_all.pdf
```

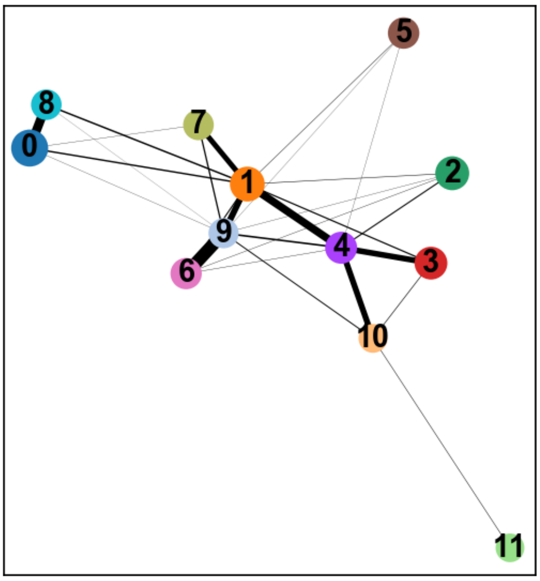

Number of cells in each cluster:

In [26]:
```
adata_proc.obs["louvain"].value_counts()
```

Out[26]:
```
0     614
1     473
2     422
3     366
4     328
5     304
6     299
7     272
8     270
9     254
10    207
11    204
Name: louvain, dtype: int64
```

Define Cell Types

DE Genes

In [27]:
```
sc.tl.rank_genes_groups(adata_proc, groupby='louvain', key_added='r
ank_genes')
sc.pl.rank_genes_groups(adata_proc, key='rank_genes', groups=['0','
1','2'], fontsize=12, save="_adata_proc_genes_1")
sc.pl.rank_genes_groups(adata_proc, key='rank_genes', groups=['3','
4','5'], fontsize=12, save="_adata_proc_genes_2")
sc.pl.rank_genes_groups(adata_proc, key='rank_genes', groups=['6','
7','8'], fontsize=12, save="_adata_proc_genes_2")
sc.pl.rank_genes_groups(adata_proc, key='rank_genes', groups=['9','
10'], fontsize=12, save="_adata_proc_genes_2")
```

```
ranking genes

/Users/david.fischer/opt/miniconda3/lib/python3.7/site-packages/sc
anpy/tools/_rank_genes_groups.py:237: RuntimeWarning: overflow enc
ountered in expm1
  foldchanges = (expm1_func(mean_group) + 1e-9) / (expm1_func(mean
_rest) + 1e-9)  # add small value to remove 0's

    finished: added to `.uns['rank_genes']`
    'names', sorted np.recarray to be indexed by group ids
    'scores', sorted np.recarray to be indexed by group ids
    'logfoldchanges', sorted np.recarray to be indexed by group id
s
    'pvals', sorted np.recarray to be indexed by group ids
    'pvals_adj', sorted np.recarray to be indexed by group ids (0:
00:00)
WARNING: saving figure to file /Users/david.fischer/phd/data/Pread
ipocytesBrown/results/panels/rank_genes_groups_louvain_adata_proc_
genes_1.pdf
```

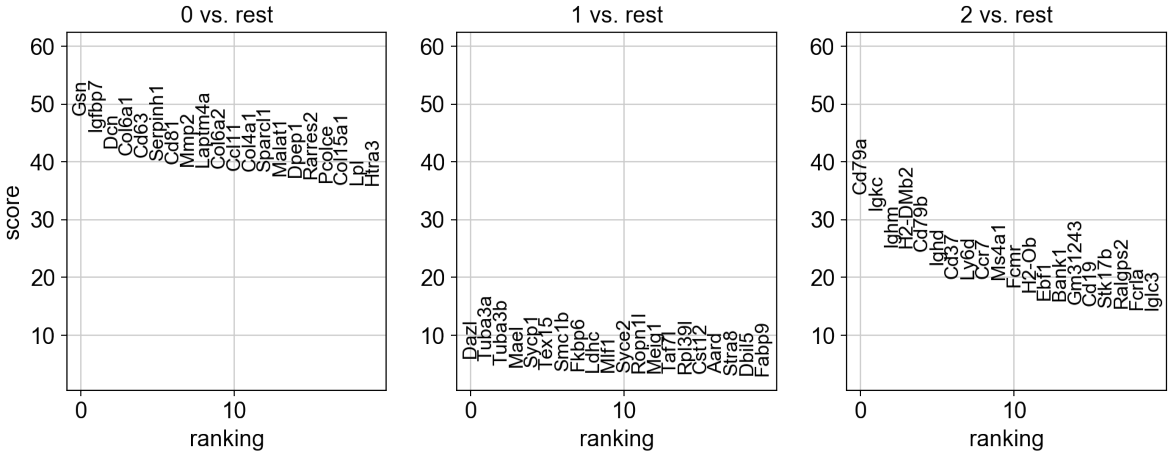


```
WARNING: saving figure to file /Users/david.fischer/phd/data/Pread
ipocytesBrown/results/panels/rank_genes_groups_louvain_adata_proc_
genes_2.pdf
```

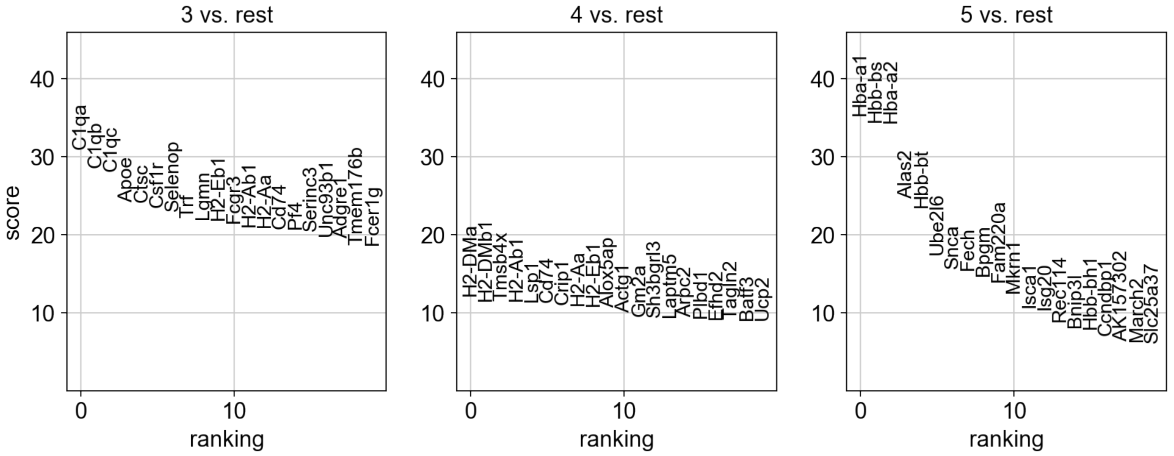

WARNING: saving figure to file /Users/david.fischer/phd/data/PreadipocytesBrown/results/panels/rank_genes_groups_louvain_adata_proc_genes_2.pdf

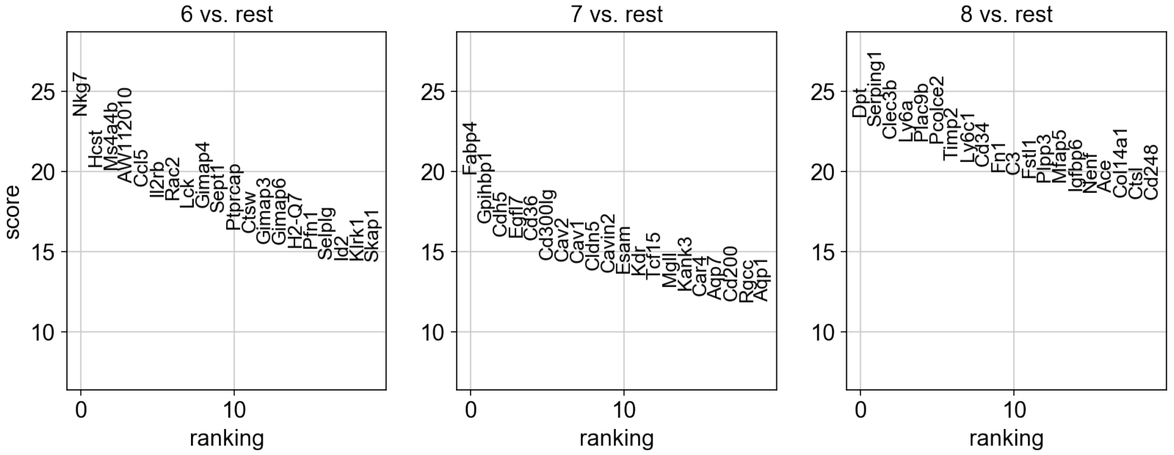

WARNING: saving figure to file /Users/david.fischer/phd/data/PreadipocytesBrown/results/panels/rank_genes_groups_louvain_adata_proc_genes_2.pdf

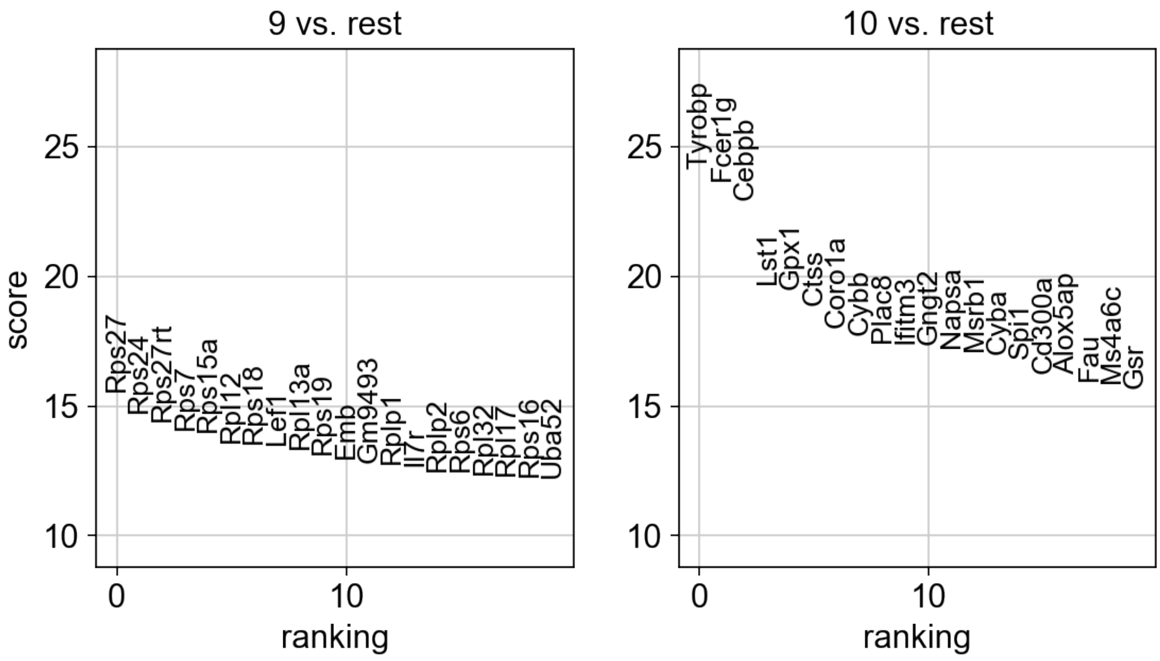

Define Marker Sets

Define marker sets for some of the expected cell types and add DE genes.

In [28]:
```
# Leukocyte markers:
leukocyte_markers = ['Ptprc','Tcf25']
tc_markers = ['Cd3d','Cd3e','Cd3g','Cd4','Cd8a1','Cd8b','Ms4a4b','T
rbc2']
nk_markers = ['Nkg7','Il2rb', 'Ncr1', 'Klrd1', 'Klrb1b', 'Klrb1f']
myeloid_markers = ['Cd79a', 'Itgax', 'Itgam', 'Fcgr3','S100a8', 'S1
00a9']
mp_markers = ['Adgre1', 'Lyz2','C1qa','Pf4']
dc_markers = ['Cd74', 'Anpep' , 'Cd33', 'Cd80', 'Cd83', 'Cd86']
bc_markers = ['Cd19','Igkc','Ighm','Cd79a']
adipocyte_markers = ['Fgf10','Bmp4','Zfp423','Psmb8','Pdgfra', 'Slc
7a10', 'Pparg', 'Cd34', 'Dlk1', 'Dpp4', 'Fabp4', 'Ucp1','P2rd5', 'S
lc36a2', 'Tmem26','Adipoq','Prdm16','Ppargc1a','Tcf25','Bin1','Eif5
','Slc2a4','Cebpa','Lpl','Pnpla2','Plin1'] # 'Slc7a10' is 'Asc1'; D
lk1 is Pref1; Cd29 is Itgb1
megakaryocyte_markers = ['Ppbp']
erythrocyte_markers = ['Gypa','Hba-a2','Hbb-bt','Alas2']
go_adip_dev = ['Aacs', 'Acat1', 'Arid5b','Arrdc3', 'Atf2', 'Bbs4',
'Bdh1',
 'Csf1', 'Dgat2','Dyrk1b', 'Ebf2', 'Fam123b', 'Fto','Hgmcs2', 'Id2'
,
 'Lep', 'Lrp5', 'Nampt', 'Oxct1', 'Paxip1', 'Pik3ca', 'Ppard', 'Ppa
rgc1a',
 'Rorc', 'Sh3pxd2b', 'Slc25a25',  'Sox8', 'Spg20', 'Tbl1xr1', 'Umol
d1', 'Xbp1', 'Znf516']
```

Only keep markers occurring in data set.

In [29]:
```
leukocyte_markers = np.array([x for x in leukocyte_markers if x in adata_proc.var_names])
tc_markers = np.array([x for x in tc_markers if x in adata_proc.var_names])
nk_markers = np.array([x for x in nk_markers if x in adata_proc.var_names])
myeloid_markers = np.array([x for x in myeloid_markers if x in adata_proc.var_names])
mp_markers = np.array([x for x in mp_markers if x in adata_proc.var_names])
dc_markers = np.array([x for x in dc_markers if x in adata_proc.var_names])
bc_markers = np.array([x for x in bc_markers if x in adata_proc.var_names])
megakaryocyte_markers = np.array([x for x in megakaryocyte_markers if x in adata_proc.var_names])
erythrocyte_markers = np.array([x for x in erythrocyte_markers if x in adata_proc.var_names])
adipocyte_markers = np.array([x for x in adipocyte_markers if x in adata_proc.var_names])
go_adip_dev = np.array([x for x in go_adip_dev if x in adata_proc.var_names])
```

Plotting Routines for Markers Gene Sets:

In [30]:
```
def plot_violin_marker(adata, markers, save=None, use_raw=True):
    for i in range(len(markers) // 2 + len(markers) % 2):
        if save is not None:
            sc.pl.violin(
                adata,
                groupby='louvain',
                keys=markers[(2*i):np.min([2*(i+1), len(markers)])]
,
                use_raw=use_raw,
                rotation=90, size=5,
                save=save+"_"+str(i)+".pdf"
            )
        else:
            sc.pl.violin(
                adata,
                groupby='louvain',
                keys=markers[(2*i):np.min([2*(i+1), len(markers)])]
,
                use_raw=use_raw,
                rotation=90, size=5,
            )
def plot_umap_marker(adata, markers, size=3, save=None, use_raw=Tru
e):
    for i in range(len(markers) // 2 + len(markers) % 2):
        print(markers[(2*i):np.min([2*(i+1), len(markers)])])
        if save is not None:
            sc.pl.umap(
                adata,
                color=markers[(2*i):np.min([2*(i+1), len(markers)])
],
                size=size,
                use_raw=use_raw,
                save=save+"_"+str(i)+".pdf"
            )
        else:
            sc.pl.umap(
                adata,
                color=markers[(2*i):np.min([2*(i+1), len(markers)])
],
                size=size,
                use_raw=use_raw
            )
```

Leukocyte Markers

In [31]:
```
if bool_plot == True:
    plot_violin_marker(adata_proc, leukocyte_markers.tolist(), save
="_all_markers_leukocyte")
```

```
WARNING: saving figure to file /Users/david.fischer/phd/data/Pread
ipocytesBrown/results/panels/violin_all_markers_leukocyte_0.pdf
```

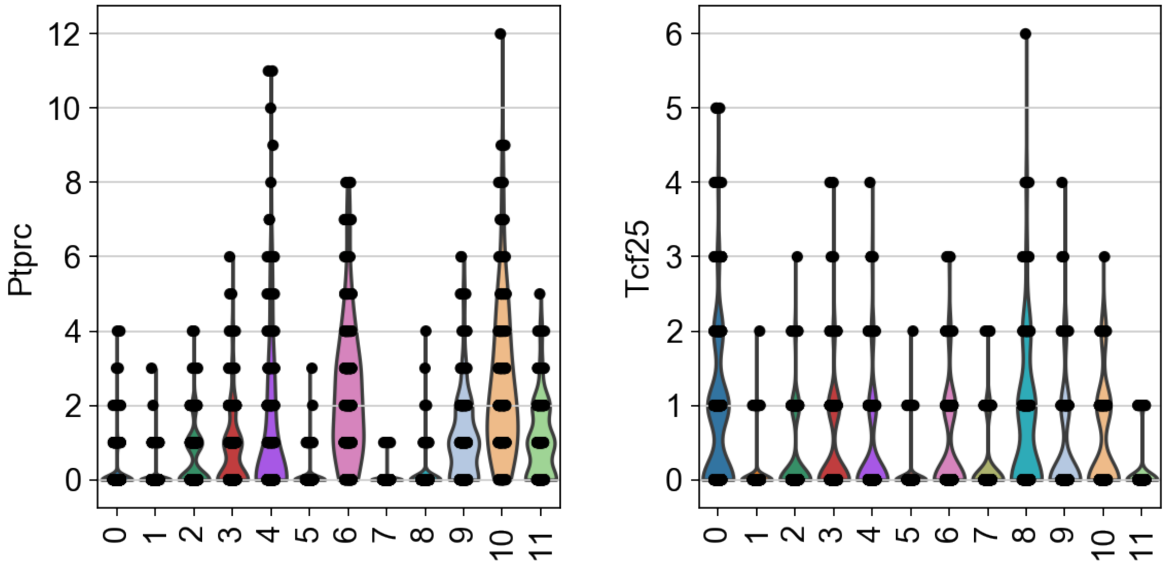

Cluster 1,9 and 10 show expression for the leukocyte marker Ptprc. Tcf25 found in many clusters.

Erythrocyte Markers

In [32]:
```
if bool_plot == True:
    plot_violin_marker(adata_proc, erythrocyte_markers.tolist(), sa
ve="_all_markers_erythrcytes")
```

```
WARNING: saving figure to file /Users/david.fischer/phd/data/Pread
ipocytesBrown/results/panels/violin_all_markers_erythrcytes_0.pdf
```

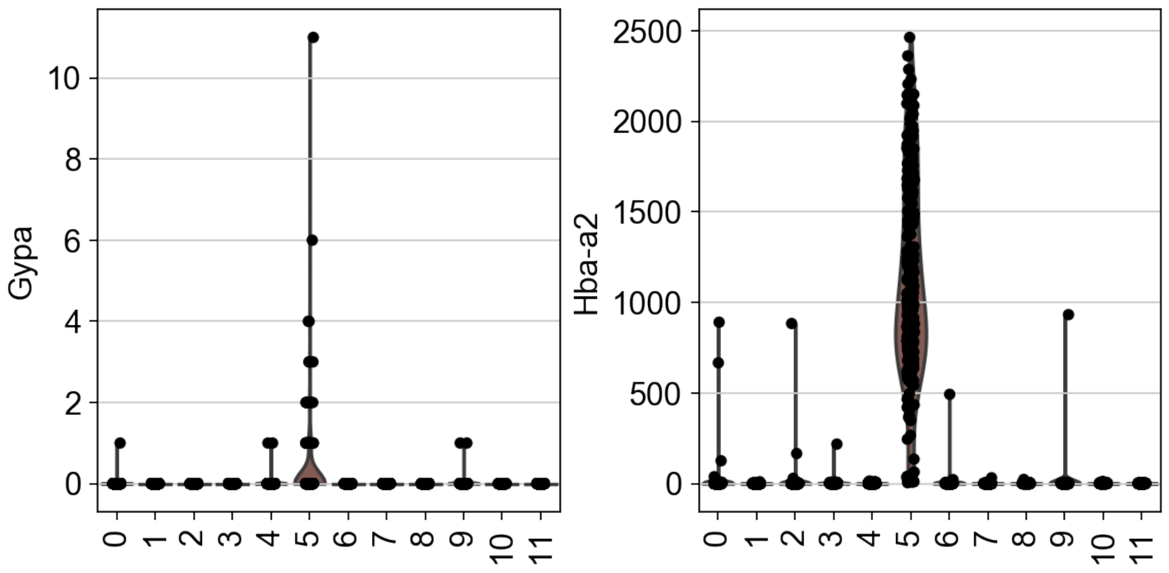


```
WARNING: saving figure to file /Users/david.fischer/phd/data/Pread
ipocytesBrown/results/panels/violin_all_markers_erythrcytes_1.pdf
```

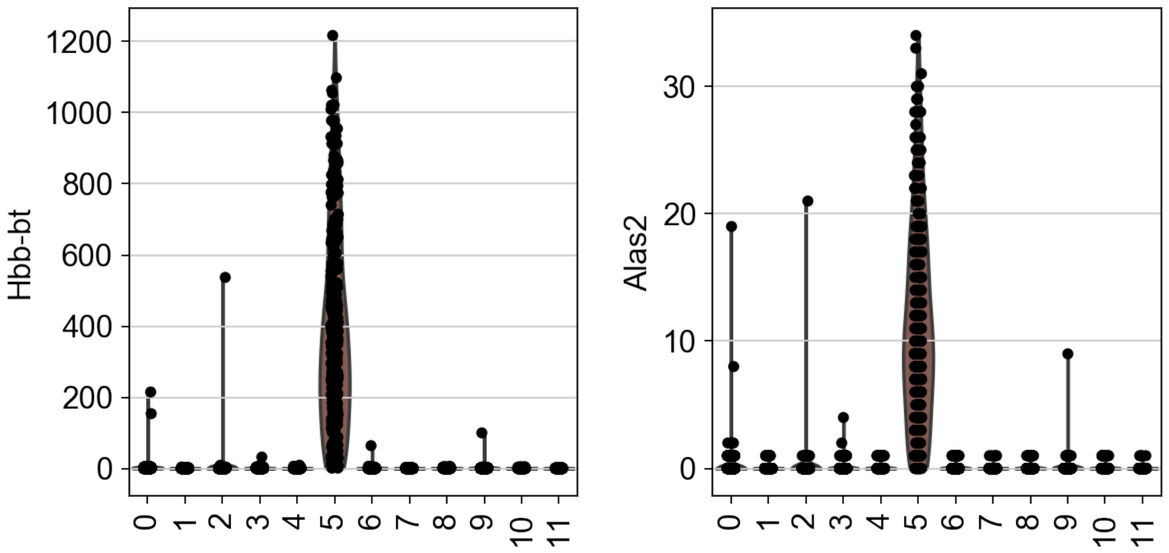

The haemoglobin markers and Alas2 show high counts in cluster 5 indicating that cluster 5 corresponds to erythrocytes.

Preadipocyte Markers

In [33]:
```
if bool_plot == True:
    plot_violin_marker(adata_proc, adipocyte_markers.tolist(), save
="_all_markers_preadipocytes")
```

```
WARNING: saving figure to file /Users/david.fischer/phd/data/Pread
ipocytesBrown/results/panels/violin_all_markers_preadipocytes_0.pd
f
```

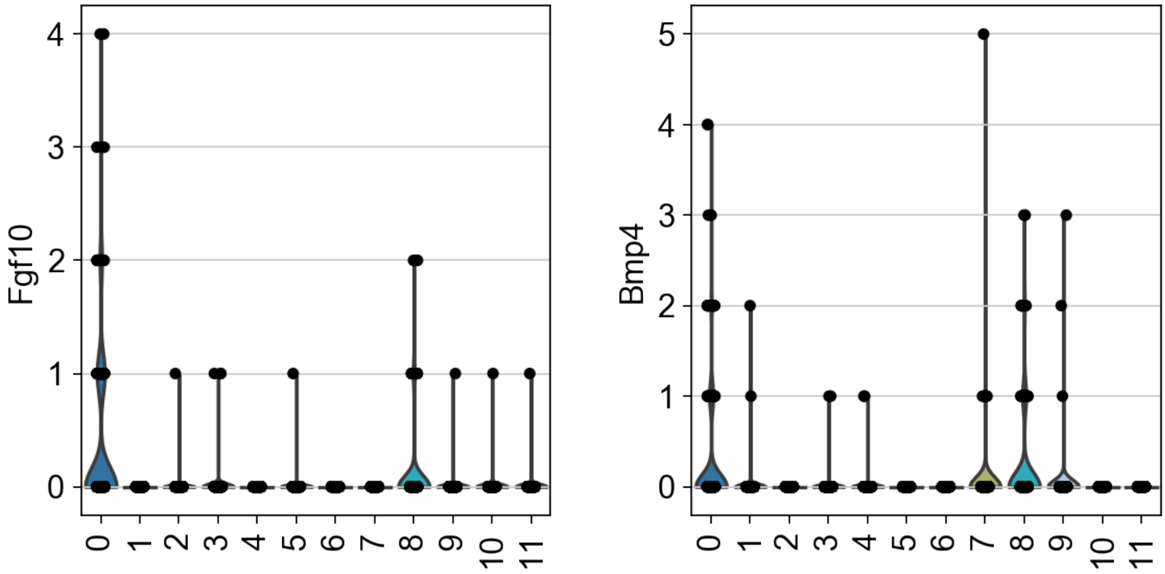


```
WARNING: saving figure to file /Users/david.fischer/phd/data/Pread
ipocytesBrown/results/panels/violin_all_markers_preadipocytes_1.pd
f
```

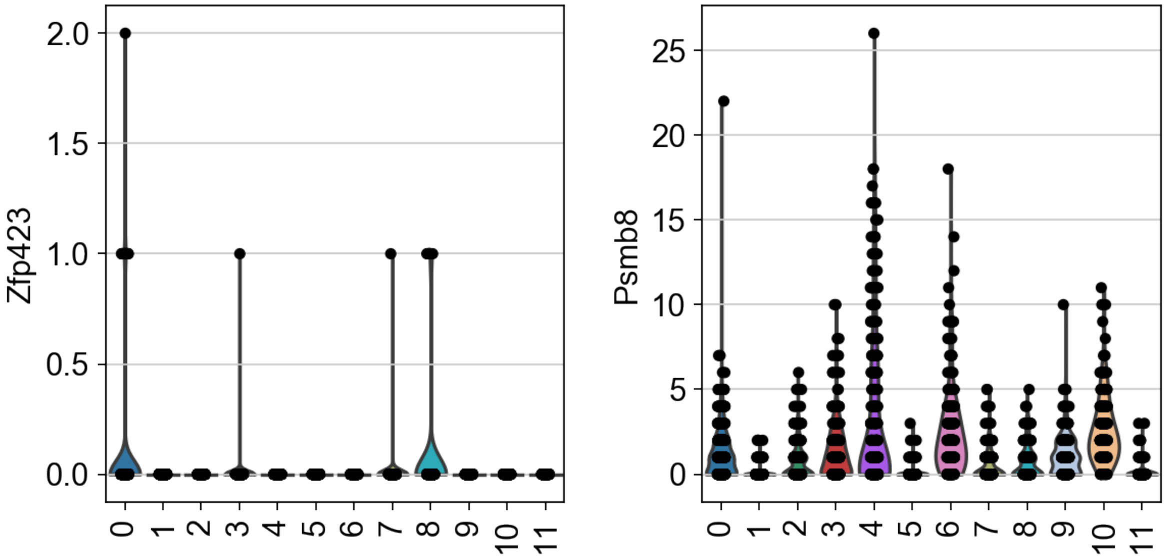


```
WARNING: saving figure to file /Users/david.fischer/phd/data/Pread
ipocytesBrown/results/panels/violin_all_markers_preadipocytes_2.pd
f
```

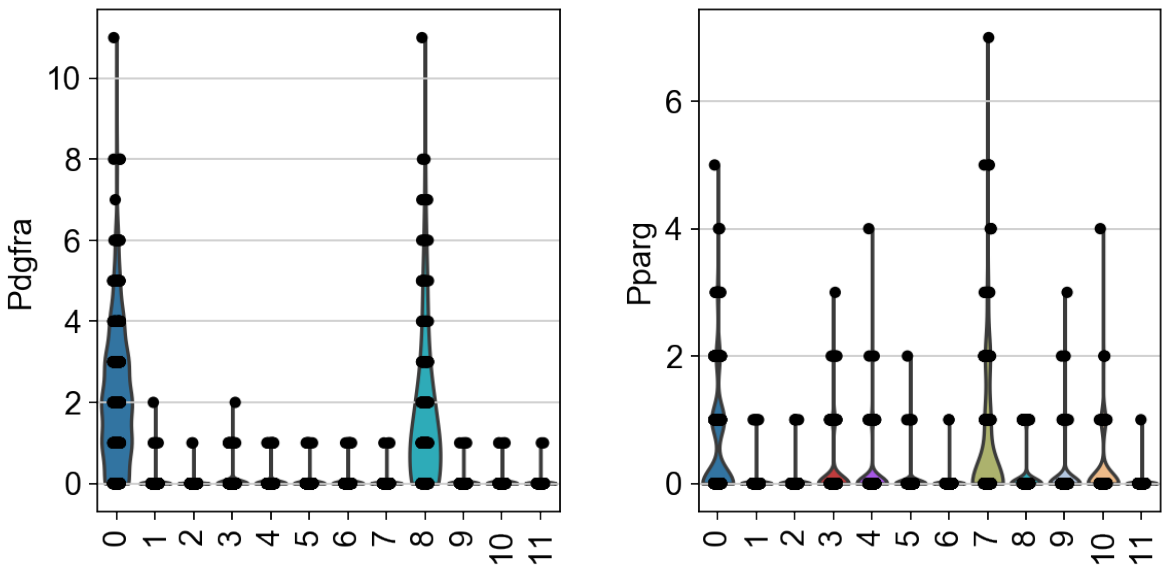


```
WARNING: saving figure to file /Users/david.fischer/phd/data/Pread
ipocytesBrown/results/panels/violin_all_markers_preadipocytes_3.pd
f
```

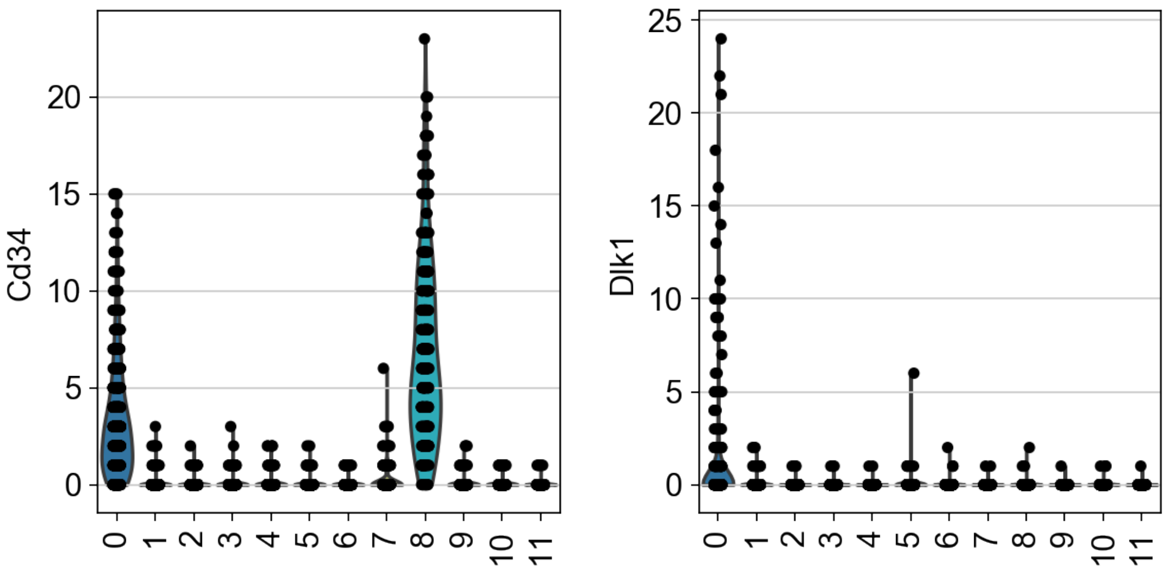


```
WARNING: saving figure to file /Users/david.fischer/phd/data/Pread
ipocytesBrown/results/panels/violin_all_markers_preadipocytes_4.pd
f
```

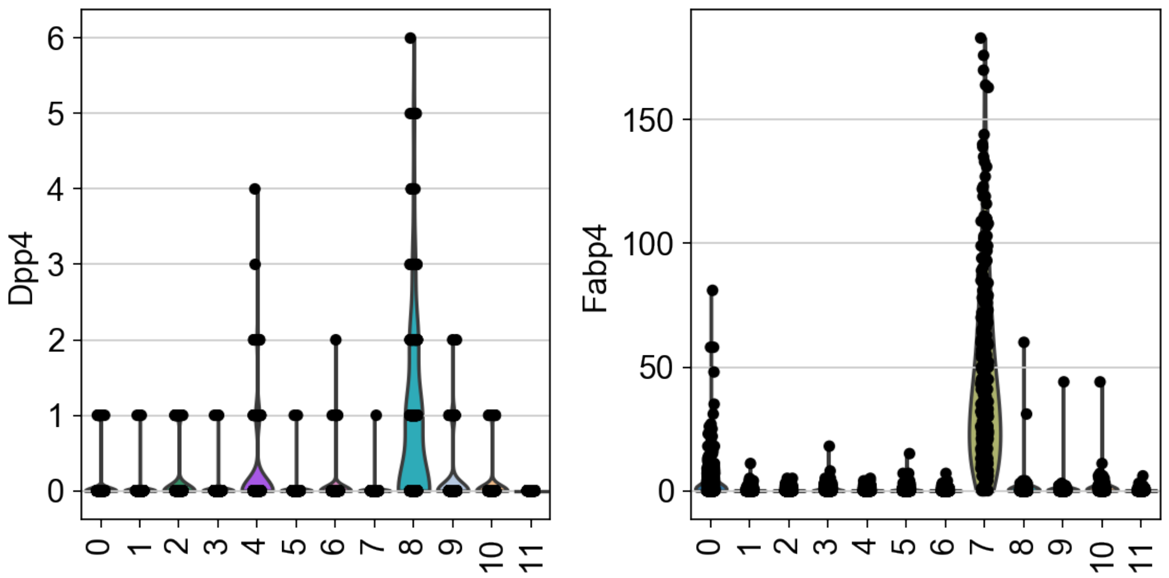


```
WARNING: saving figure to file /Users/david.fischer/phd/data/Pread
ipocytesBrown/results/panels/violin_all_markers_preadipocytes_5.pd
f
```

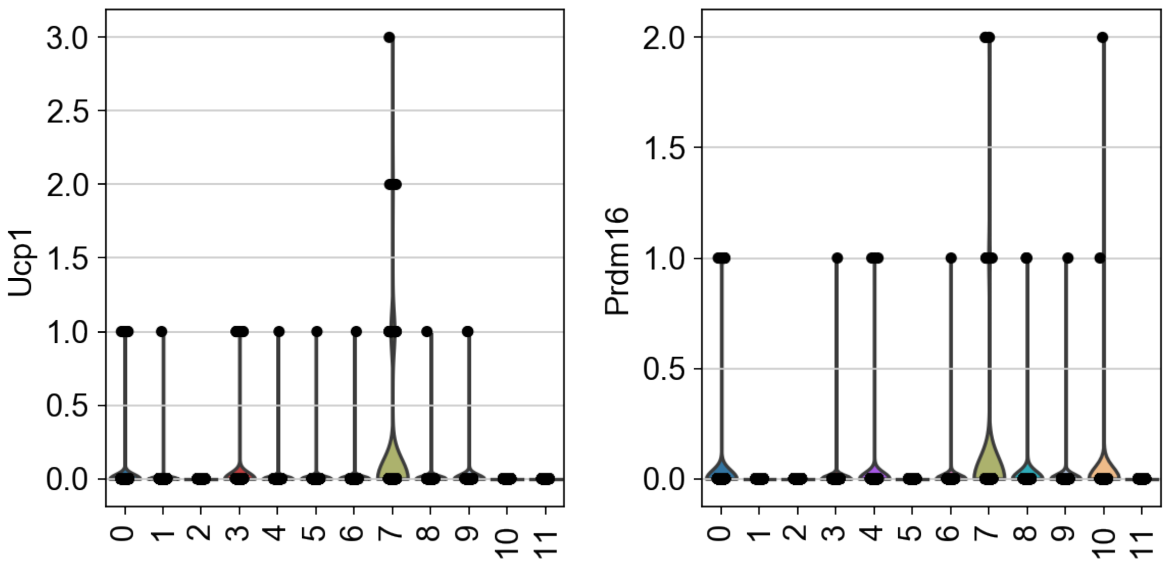


```
WARNING: saving figure to file /Users/david.fischer/phd/data/Pread
ipocytesBrown/results/panels/violin_all_markers_preadipocytes_6.pd
f
```

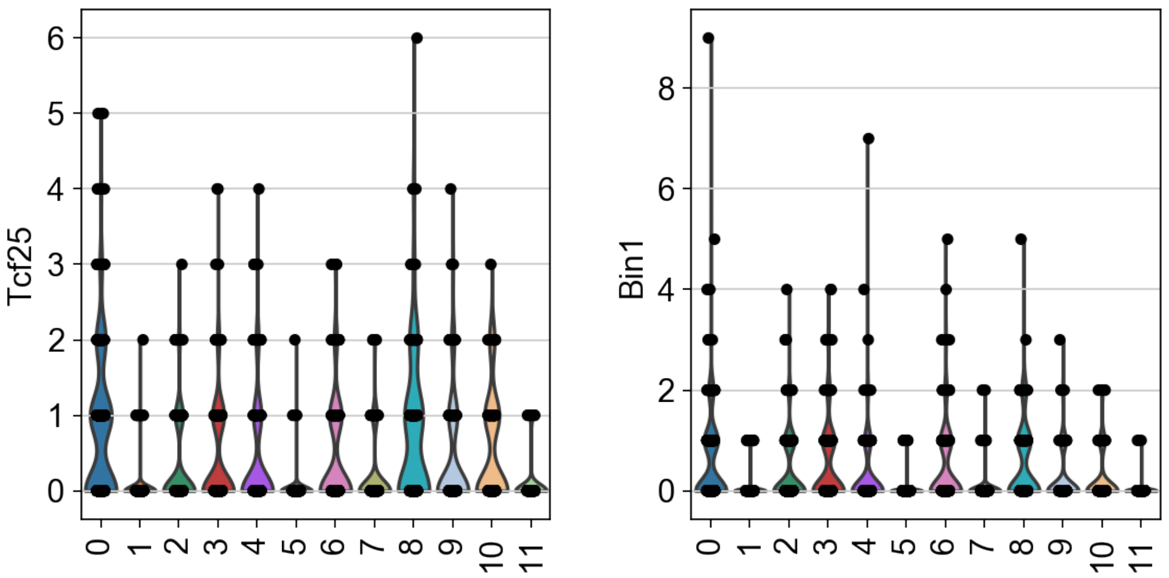

WARNING: saving figure to file /Users/david.fischer/phd/data/PreadipocytesBrown/results/panels/violin_all_markers_preadipocytes_7.pdf

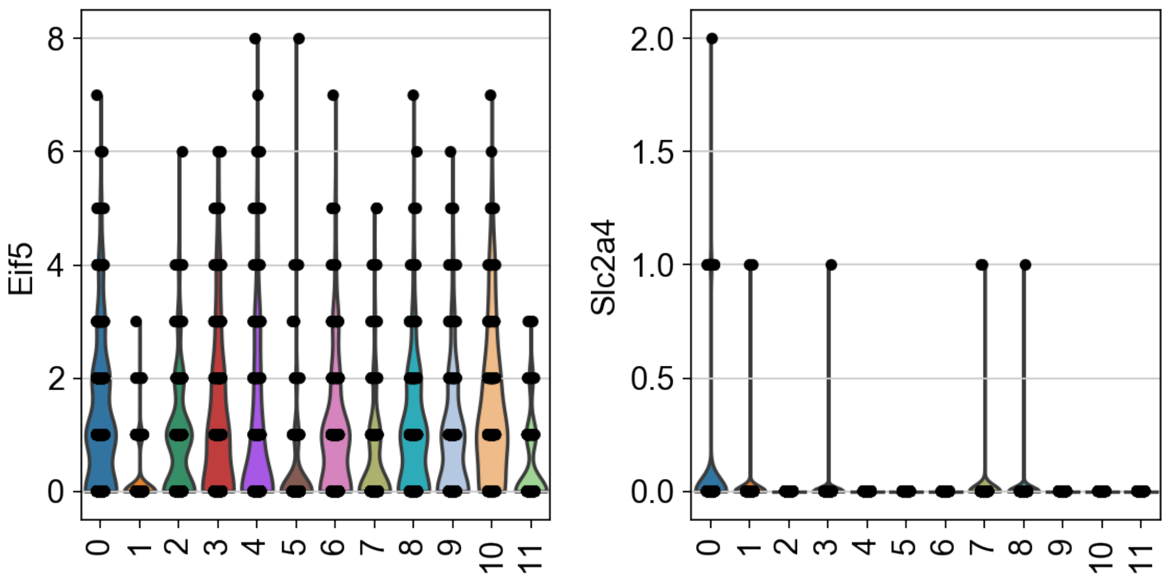

WARNING: saving figure to file /Users/david.fischer/phd/data/PreadipocytesBrown/results/panels/violin_all_markers_preadipocytes_8.pdf

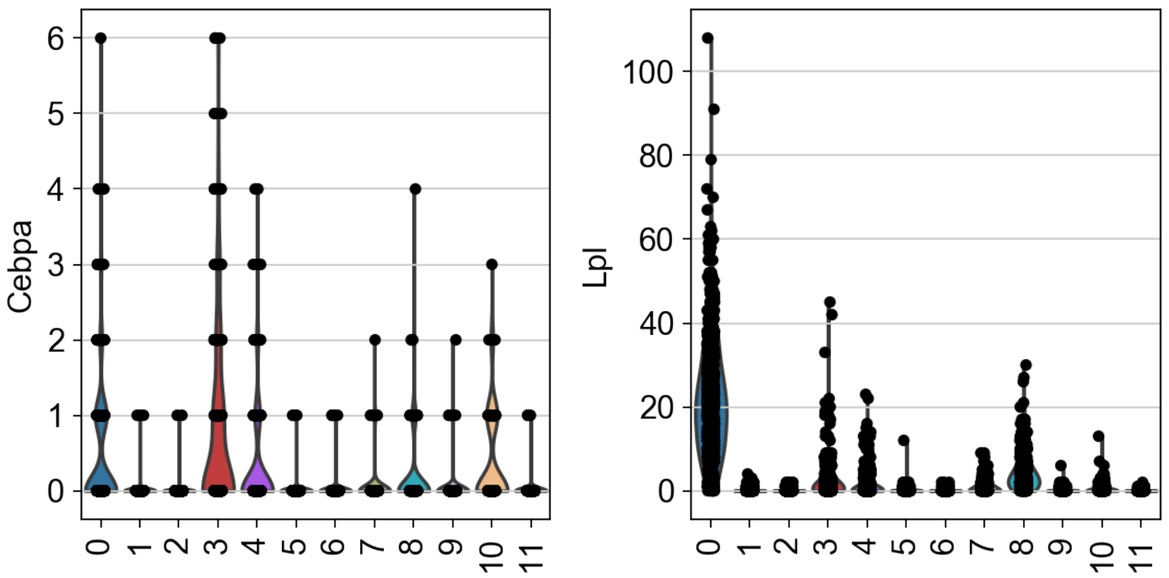

WARNING: saving figure to file /Users/david.fischer/phd/data/PreadipocytesBrown/results/panels/violin_all_markers_preadipocytes_9.pdf

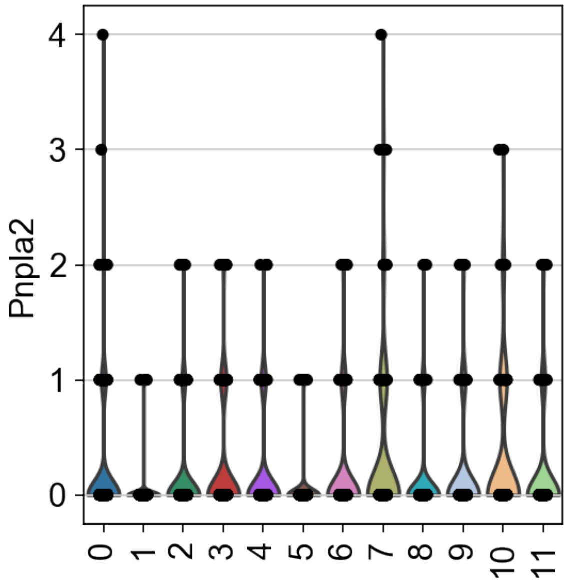

T-Cell Markers

In [34]:
```
if bool_plot == True:
    plot_violin_marker(adata_proc, tc_markers.tolist(), save="_all_markers_tcells")
```

WARNING: saving figure to file /Users/david.fischer/phd/data/PreadipocytesBrown/results/panels/violin_all_markers_tcells_0.pdf

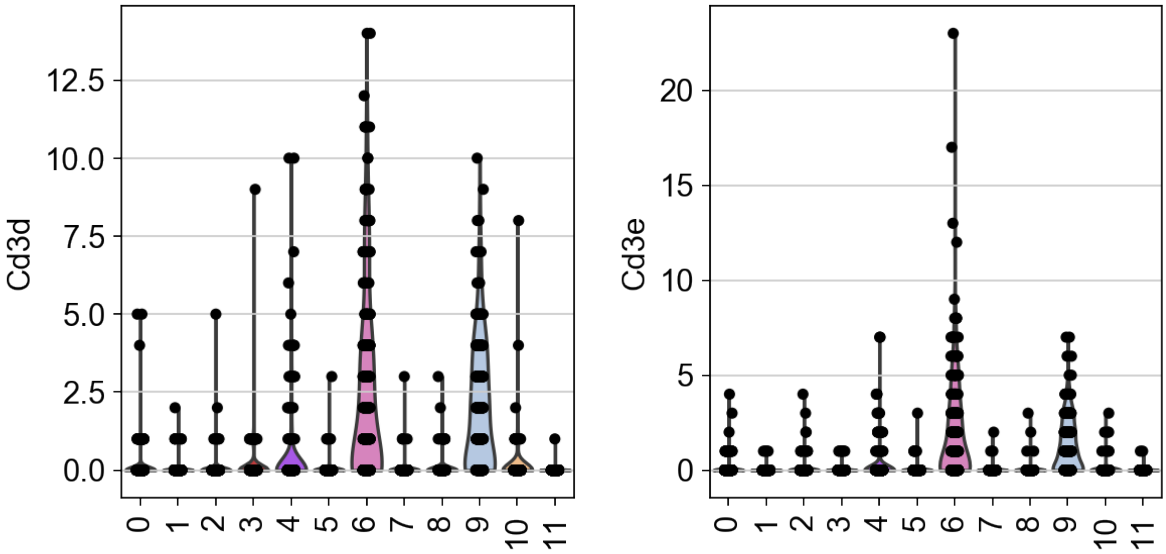


```
WARNING: saving figure to file /Users/david.fischer/phd/data/Pread
ipocytesBrown/results/panels/violin_all_markers_tcells_1.pdf
```

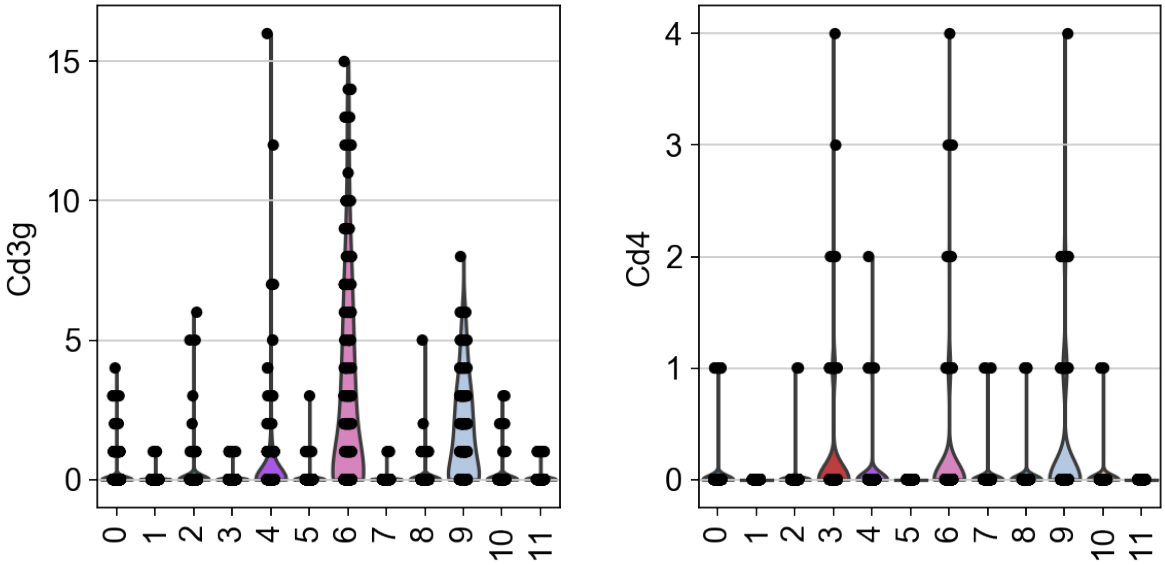


```
WARNING: saving figure to file /Users/david.fischer/phd/data/Pread
ipocytesBrown/results/panels/violin_all_markers_tcells_2.pdf
```

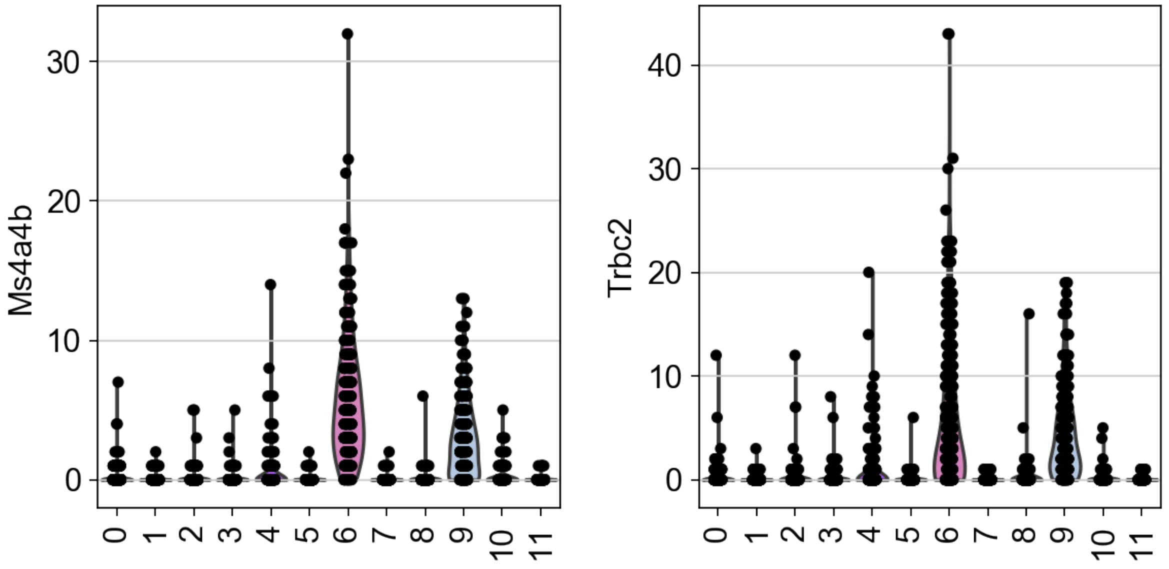

T-cell markers are strongly expressed in cluster 6.

Natural Killer Cells Markers

In [35]:
```
if bool_plot == True:
    plot_violin_marker(adata_proc, nk_markers.tolist(), save="_all_markers_nk")
```

```
WARNING: saving figure to file /Users/david.fischer/phd/data/Preadipocytes Brown/results/panels/violin_all_markers_nk_0.pdf
```

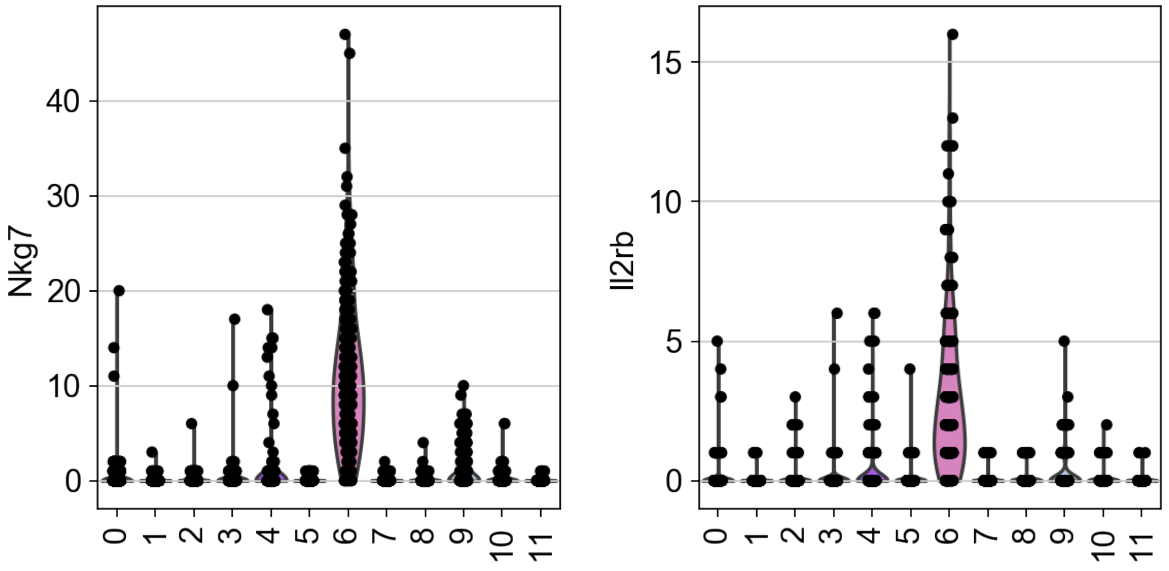


```
WARNING: saving figure to file /Users/david.fischer/phd/data/Preadipocytes Brown/results/panels/violin_all_markers_nk_1.pdf
```

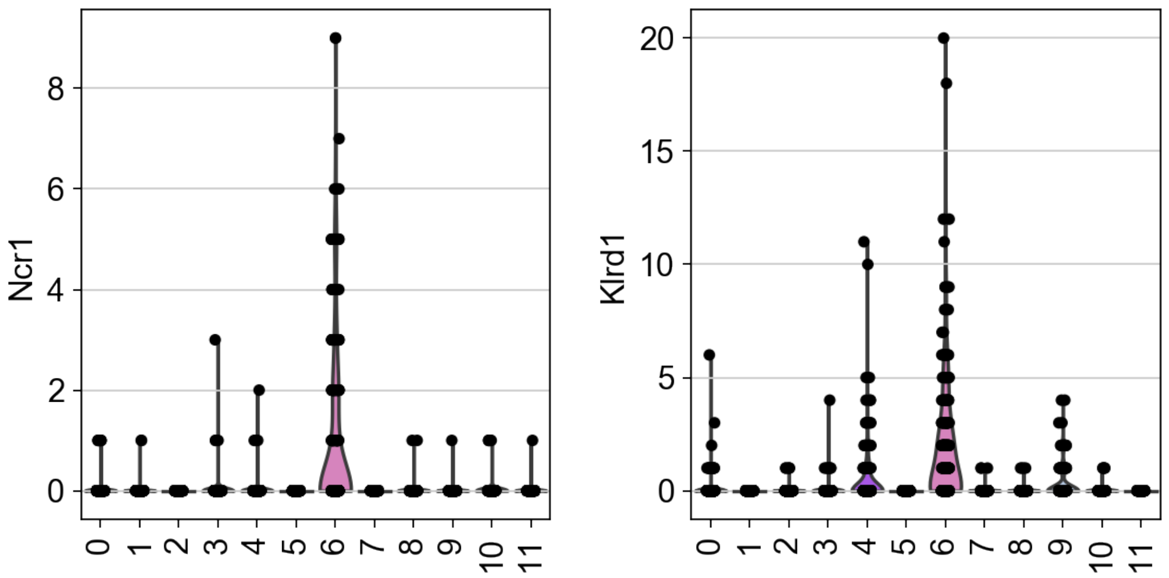

WARNING: saving figure to file /Users/david.fischer/phd/data/PreadipocytesBrown/results/panels/violin_all_markers_nk_2.pdf

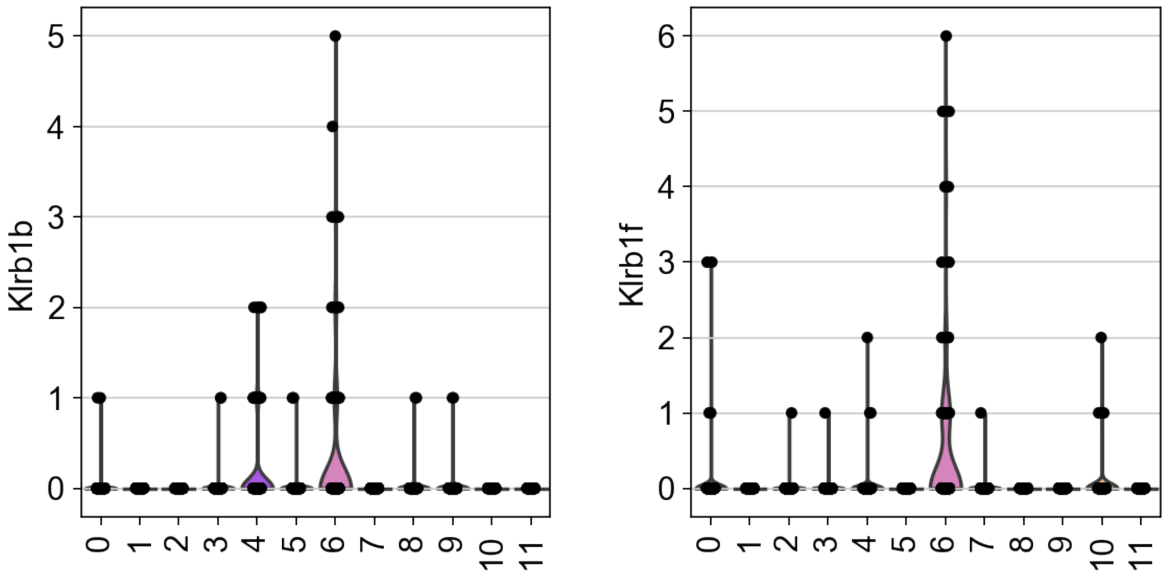

NK cell markers are expressed in cluster 6, which is inline with their transcriptomic similarity to T cells which are also found in cluster 6.

Myeloid Cell Markers

In [36]:
```
if bool_plot == True:
    plot_violin_marker(adata_proc, myeloid_markers.tolist(), save="_all_markers_myeloid")
```

WARNING: saving figure to file /Users/david.fischer/phd/data/PreadipocytesBrown/results/panels/violin_all_markers_myeloid_0.pdf

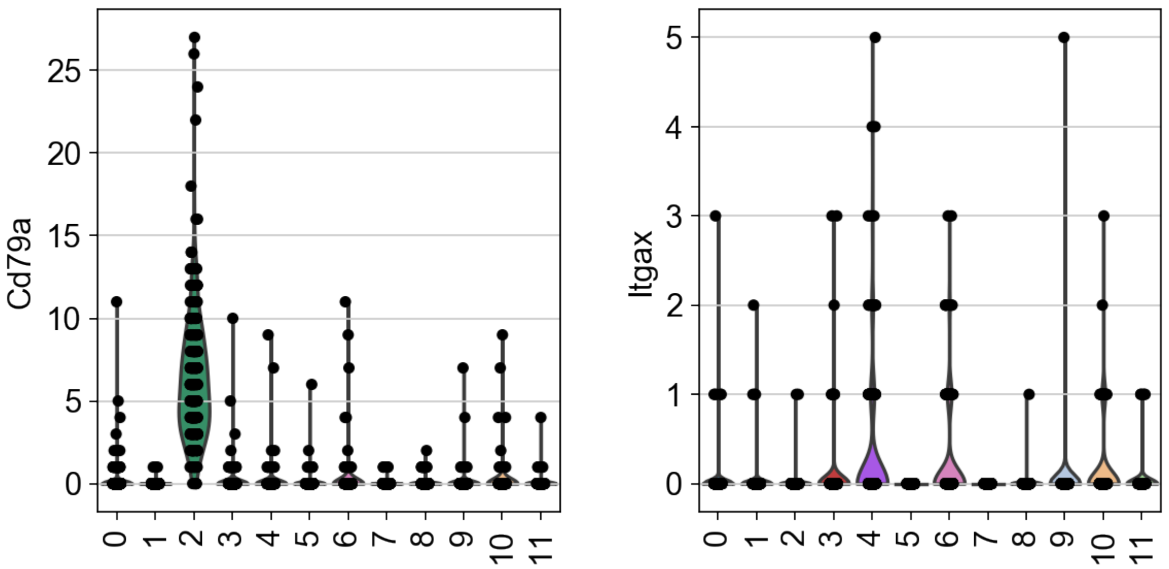

WARNING: saving figure to file /Users/david.fischer/phd/data/Preadipocytes Brown/results/panels/violin_all_markers_myeloid_1.pdf

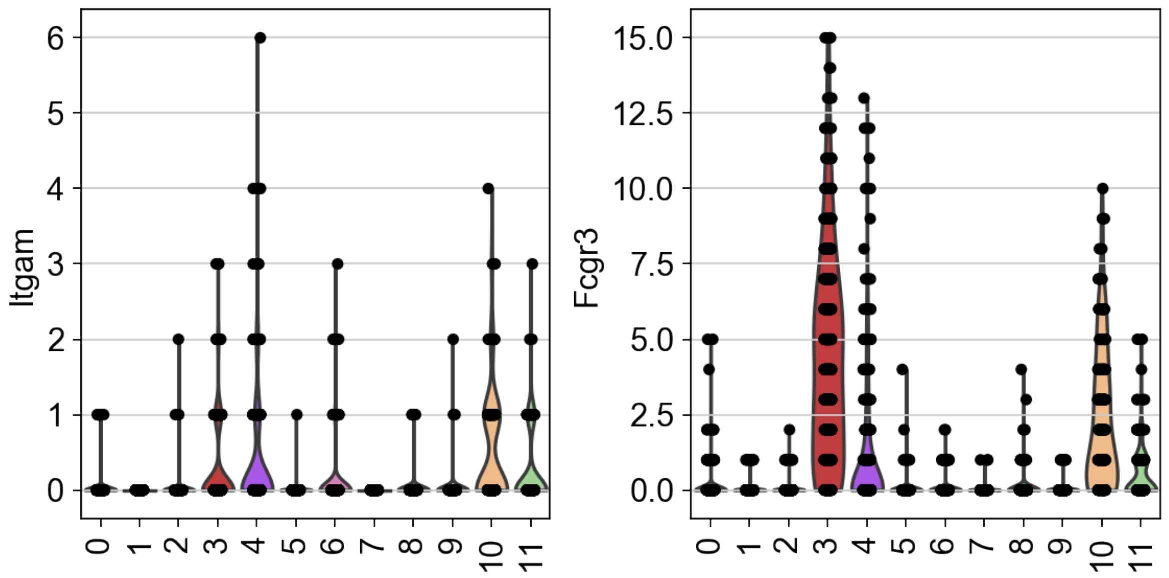

WARNING: saving figure to file /Users/david.fischer/phd/data/Preadipocytes Brown/results/panels/violin_all_markers_myeloid_2.pdf

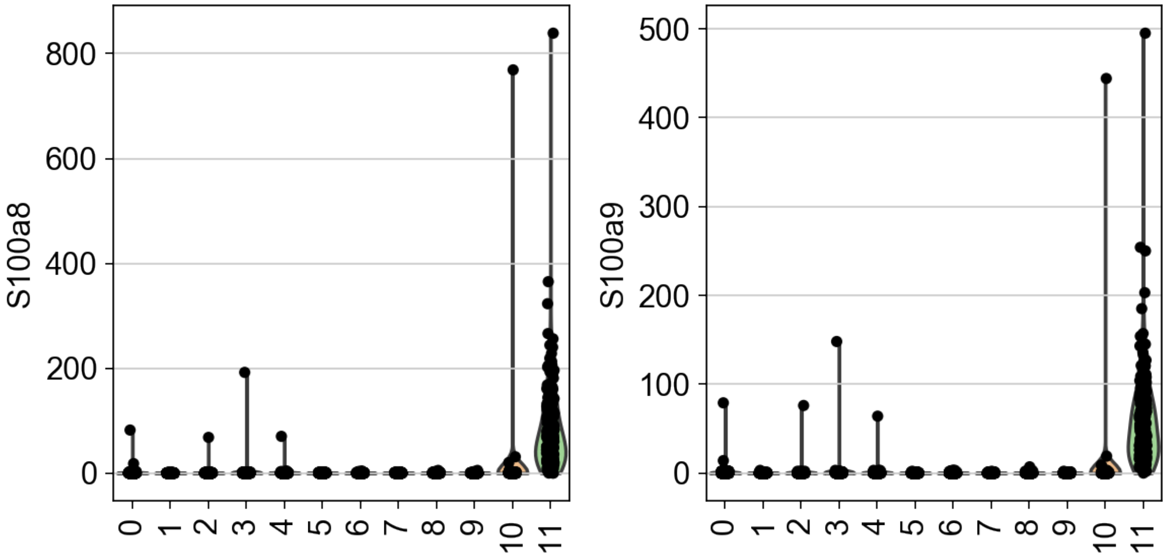

Macrophage Markers

In [37]:
```
if bool_plot == True:
    plot_violin_marker(adata_proc, mp_markers.tolist(), save="_all_markers_macrophages")
```

```
WARNING: saving figure to file /Users/david.fischer/phd/data/Pread
ipocytesBrown/results/panels/violin_all_markers_macrophages_0.pdf
```

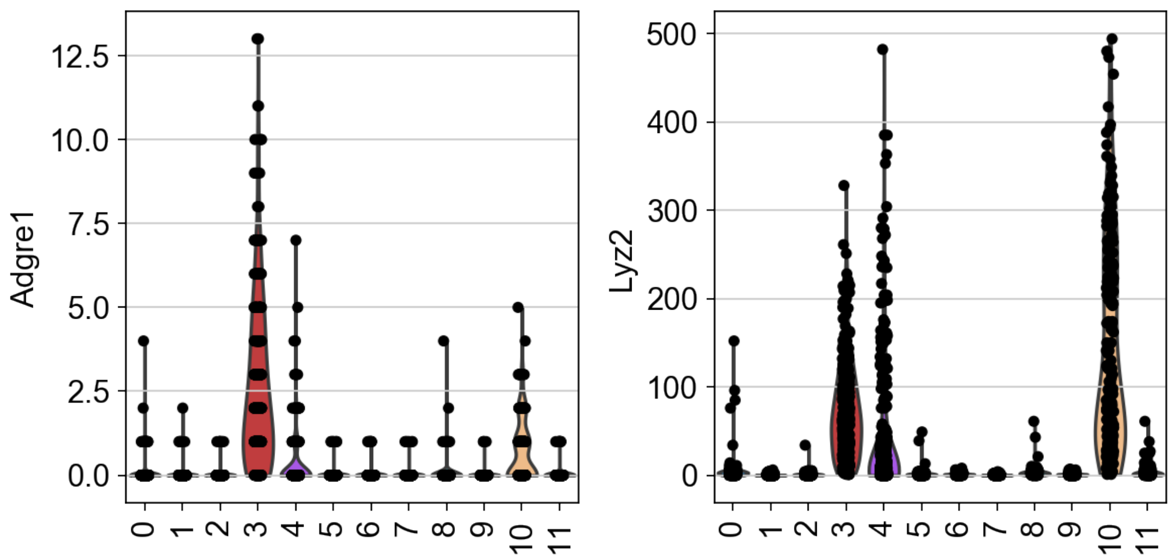


```
WARNING: saving figure to file /Users/david.fischer/phd/data/Pread
ipocytesBrown/results/panels/violin_all_markers_macrophages_1.pdf
```

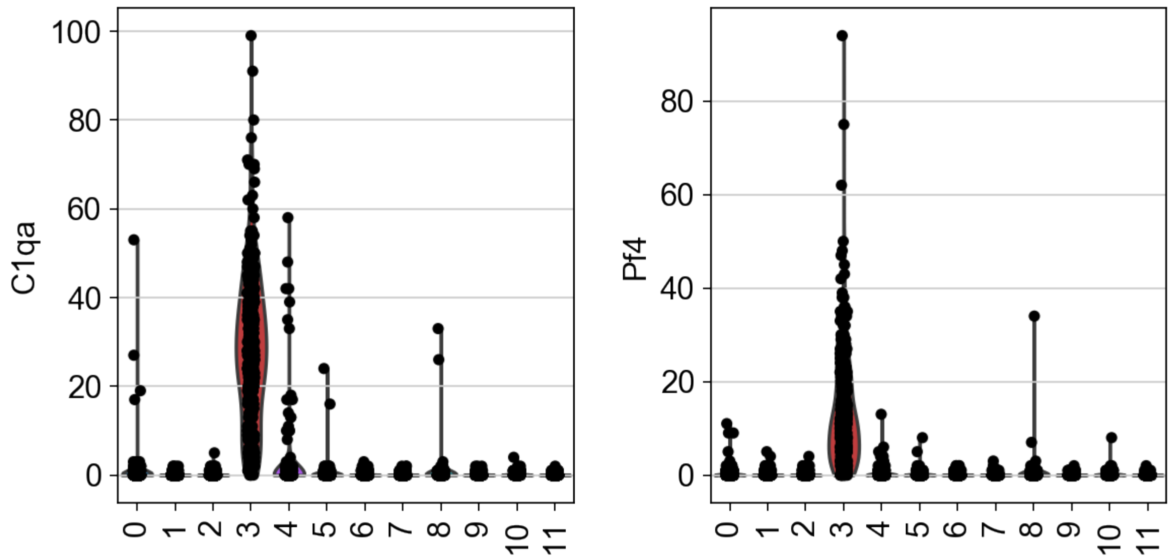

The macrophage markers are strongly expressed in cluster 3.

Dendritic cell Markers

In [38]:
```
if bool_plot == True:
    plot_violin_marker(adata_proc, dc_markers.tolist(), save="_all_
markers_dendritic")
```

```
WARNING: saving figure to file /Users/david.fischer/phd/data/Pread
ipocytesBrown/results/panels/violin_all_markers_dendritic_0.pdf
```

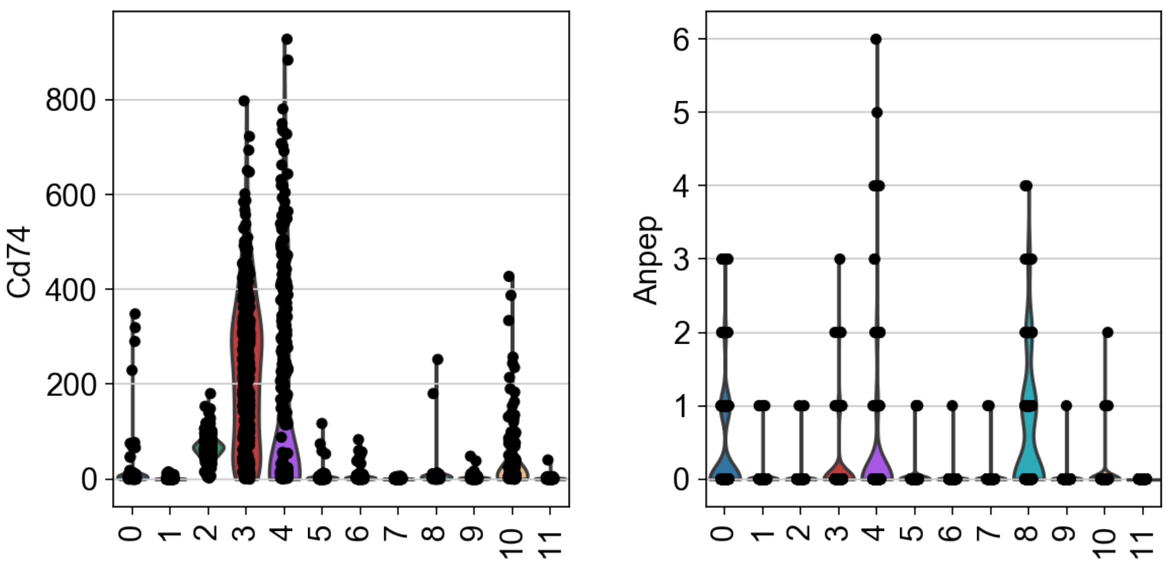


```
WARNING: saving figure to file /Users/david.fischer/phd/data/Pread
ipocytesBrown/results/panels/violin_all_markers_dendritic_1.pdf
```

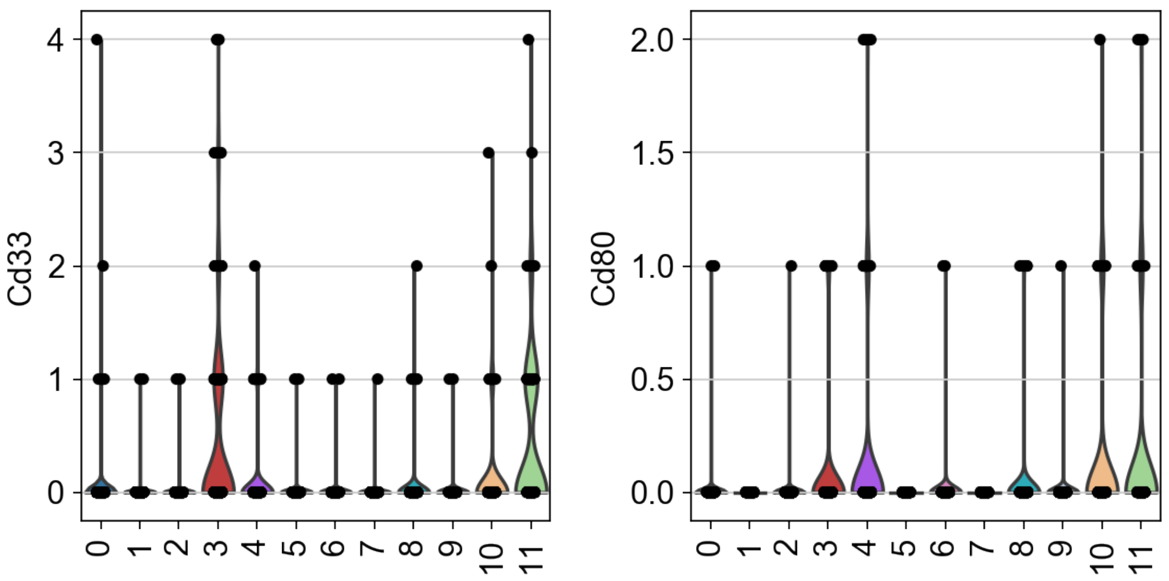


```
WARNING: saving figure to file /Users/david.fischer/phd/data/Pread
ipocytesBrown/results/panels/violin_all_markers_dendritic_2.pdf
```

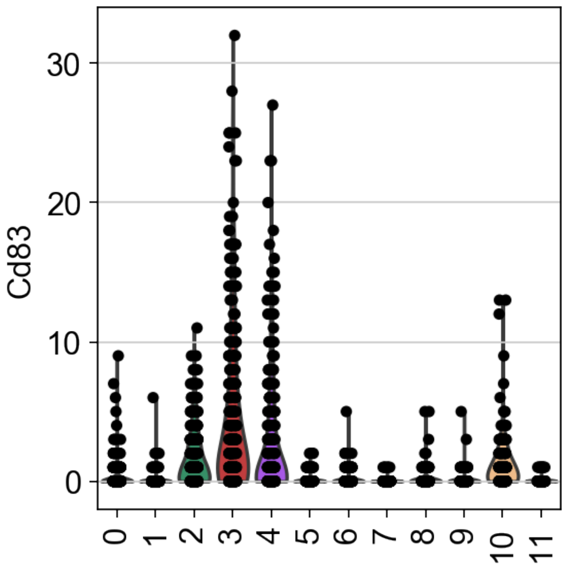

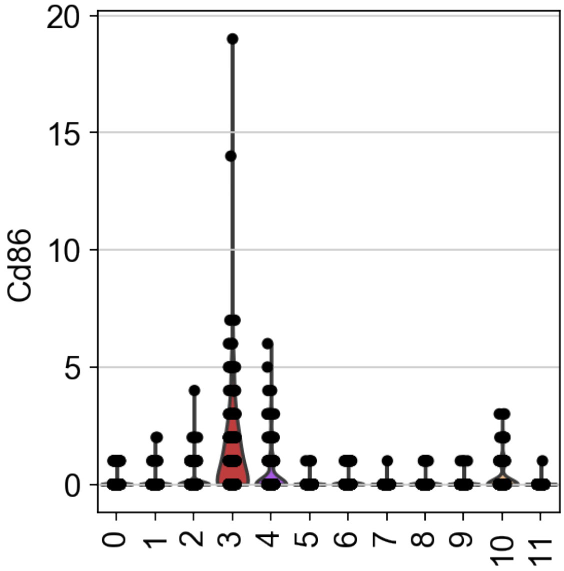

Dendritic cells markers are stongly expressed in cluster 3 and 4.

B-Cell Markers

In [39]:
```
if bool_plot == True:
    plot_violin_marker(adata_proc, bc_markers.tolist(), save="_all_markers_bcells")
```

```
WARNING: saving figure to file /Users/david.fischer/phd/data/Pread
ipocytesBrown/results/panels/violin_all_markers_bcells_0.pdf
```

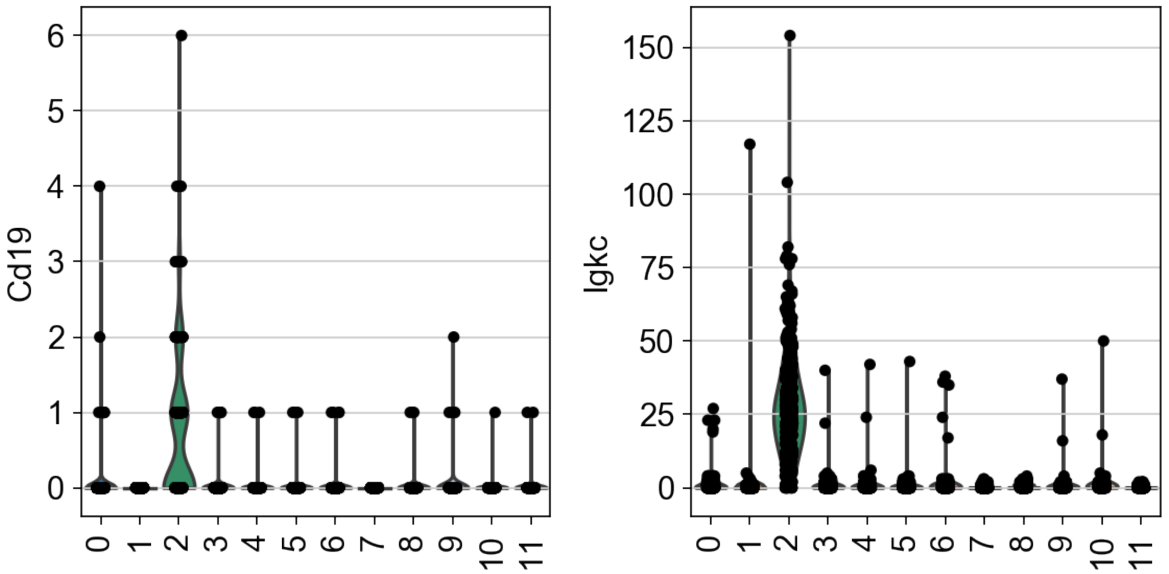


```
WARNING: saving figure to file /Users/david.fischer/phd/data/Pread
ipocytesBrown/results/panels/violin_all_markers_bcells_1.pdf
```

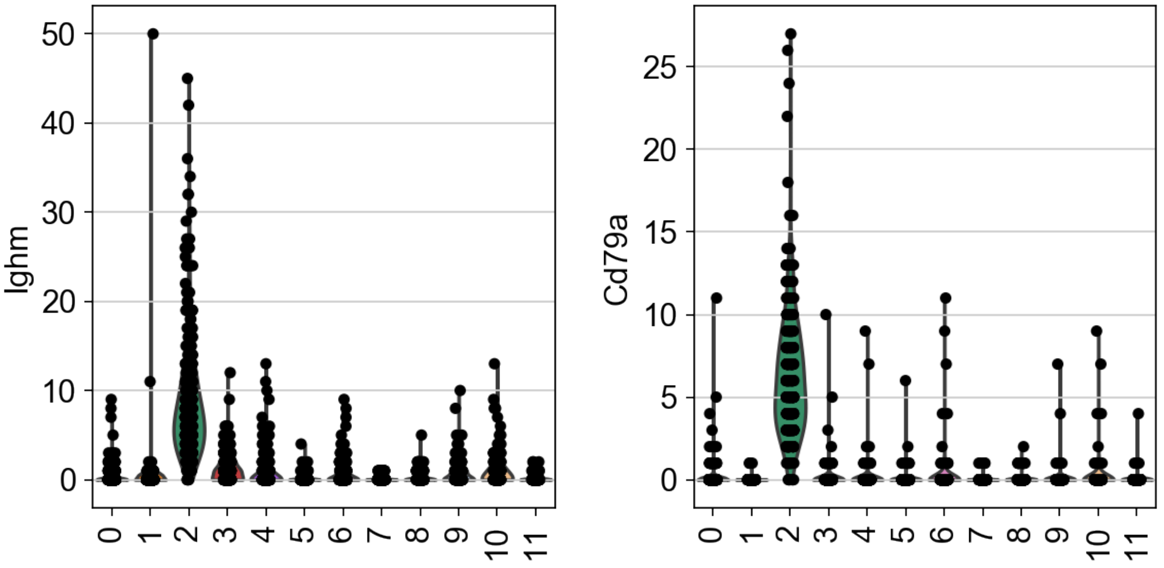

B cell markers are highly expressed in cluster 2.

Summary Heatmap for cluster assignments

In [40]:
```
selected_leukocyte_markers = ['Ptprc']
selected_tc_markers = ['Cd3d', 'Cd3g','Cd3e']
selected_nk_markers = ['Nkg7','Klrd1','Ncr1','Il2rb']
selected_myeloid_markers = ['Fcgr3','S100a8']
selected_mp_markers = ['Adgre1', 'C1qa','Pf4']
selected_dc_markers = ['Cd74', 'Cd83','Cd86']
selected_bc_markers = ['Cd19','Igkc','Ighm','Cd79a']
selected_adipocyte_markers = ['Pdgfra','Cd34', 'Dlk1','Fabp4','Fgf1
0','Bmp4','Pparg','Ucp1','Prdm16','Lpl'] # 'Slc7a10' is 'Asc1'; Dlk
1 is Pref1; Cd29 is Itgb1
#selected_megakaryocyte_markers = ['Ppbp']
selected_erythrocyte_markers = ['Gypa','Hba-a2','Hbb-bt','Alas2']
```

In [41]:
```
selected_cell_markers = selected_leukocyte_markers + \
selected_erythrocyte_markers + \
selected_myeloid_markers + \
selected_mp_markers + \
selected_dc_markers + \
selected_bc_markers + \
selected_tc_markers + \
selected_nk_markers + \
selected_adipocyte_markers
```

In [42]:
```
if bool_plot==True:
    sc.pl.heatmap(
        adata=adata_proc,
        var_names=selected_cell_markers,
        groupby="louvain",
        use_raw=False,
        log=False,
        dendrogram=True,
        var_group_rotation=90,
        show_gene_labels=True,
        show=True,
        save="_all_markers_celltypes.pdf"
    )
```

```
WARNING: dendrogram data not found (using key=dendrogram_louvain).
Running `sc.tl.dendrogram` with default parameters. For fine tunin
g it is recommended to run `sc.tl.dendrogram` independently.
    using 'X_pca' with n_pcs = 50
Storing dendrogram info using `.uns['dendrogram_louvain']`
WARNING: saving figure to file /Users/david.fischer/phd/data/Pread
ipocytesBrown/results/panels/heatmap_all_markers_celltypes.pdf
```

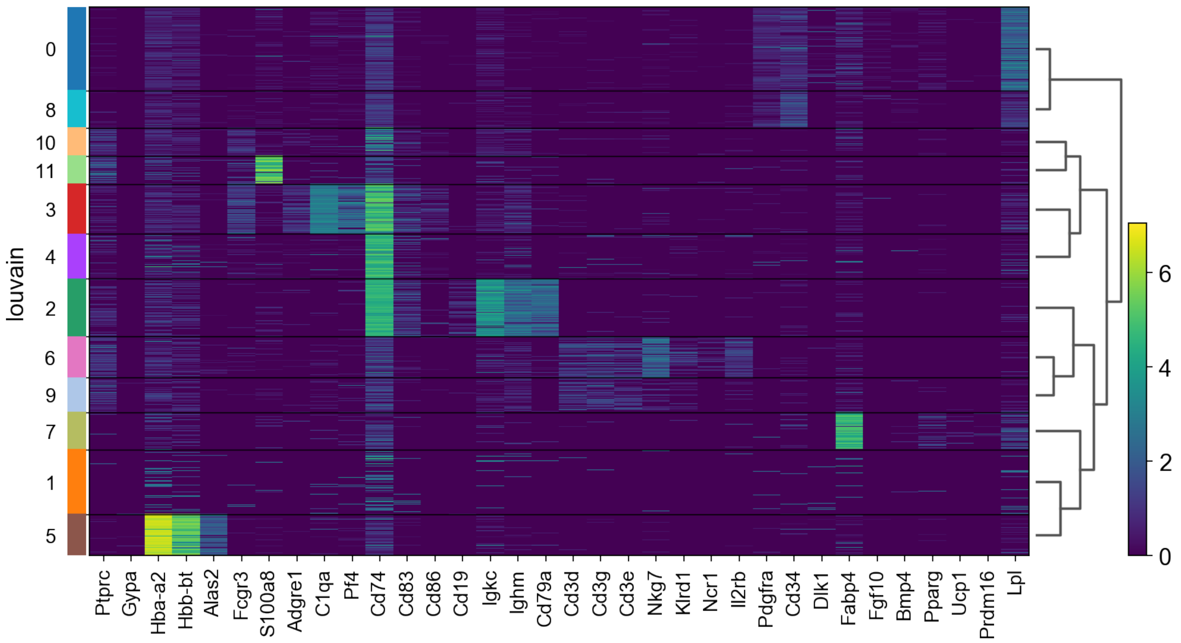

UMAP with assigned cell types

In [43]:
```
new_cluster_names = {
    '0': "preadipocytes",
    '1': "low expression",
    '2': "lymphoid (B cell)",
    '3': "myeloid (DC, macrophage)",
    '4': "myeloid (DC)",
    '5': "erythrocytes",
    '6': "lymphoid (TC, NK)",
    '7': "adipocytes",
    '8': "preadipocytes",
    '9': "lymphoid (TC)",
    '10': "myeloid (macrophage)",
    '11': "myeloid (s100a8 high)"
}
adata_proc.obs['celltypes'] = [new_cluster_names[x] for x in  adata_proc.obs['louvain']]
if bool_plot == True:
    sc.pl.umap(adata_proc, size=5, color=['celltypes'], save="_all_louvain_named.pdf")
    sc.pl.paga(adata_proc, color=['celltypes'], save="_all_named.pdf")
    #sc.pl.umap(adata_proc, size=5, color=adipocyte_markers)
```

```
... storing 'celltypes' as categorical

WARNING: saving figure to file /Users/david.fischer/phd/data/Pread
ipocytesBrown/results/panels/umap_all_louvain_named.pdf
```

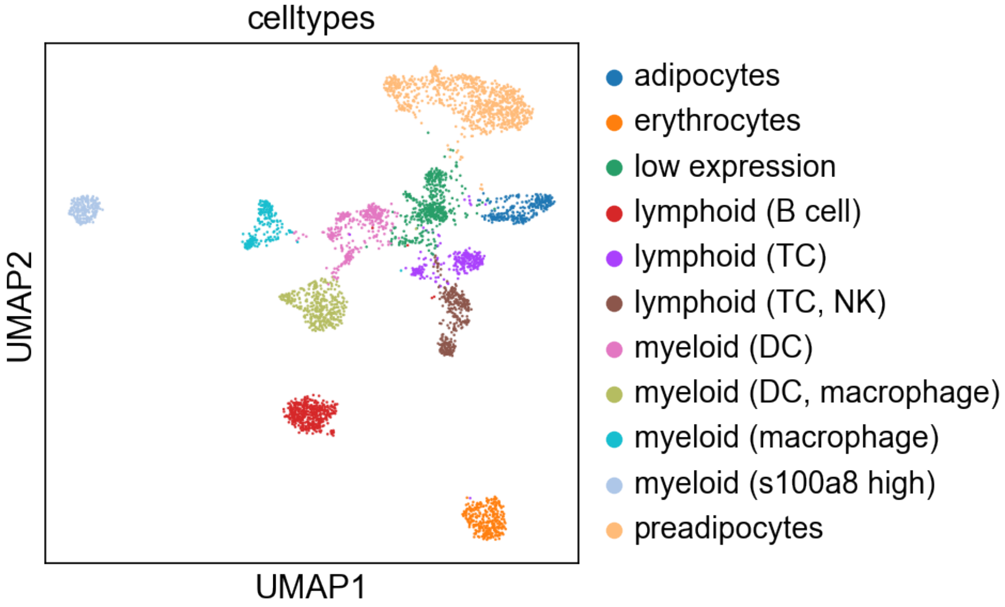


```
--> added 'pos', the PAGA positions (adata.uns['paga'])
WARNING: saving figure to file /Users/david.fischer/phd/data/Pread
ipocytesBrown/results/panels/paga_all_named.pdf
```

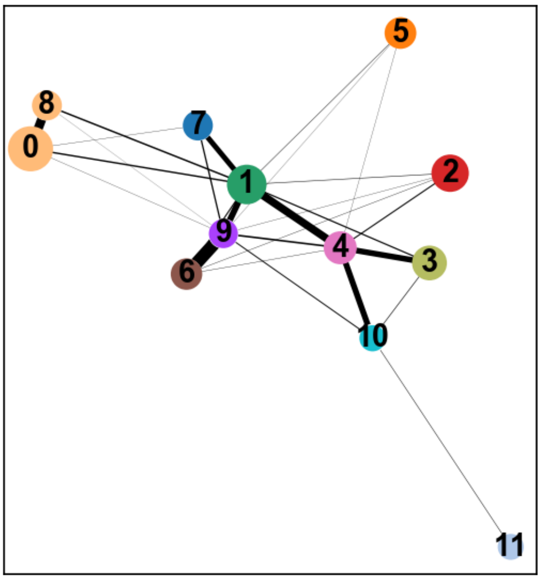

Adipocytes only

Embedding and Clustering

In [44]:
```
if bool_recomp == True:
    cell_ids_adip = np.asarray(adata_proc.obs_names)[
        [x in ['preadipocytes','adipocytes']
         for x in np.asarray(adata_proc.obs['celltypes'].values)]
    ]
    adata_adip = adata_raw[cell_ids_adip,:].copy()
    adata_adip.obs['n_genes'] = (adata_adip.X > 0).sum(1)
    mt_gene_mask = [gene.startswith('mt-') for gene in adata_adip.v
ar_names]
    temp_mt_sum = adata_adip[:,mt_gene_mask].X.sum(1)
    temp_mt_sum = np.squeeze(np.asarray(temp_mt_sum))
    temp_n_counts = adata_adip.obs['n_counts']
    adata_adip.obs['mt_frac'] = temp_mt_sum/adata_adip.obs['n_count
s']
    adata_adip.raw = adata_adip
    sc.pp.normalize_per_cell(adata_adip)
    sc.pp.log1p(adata_adip)
    sc.pp.highly_variable_genes(adata_adip,n_top_genes=4000)
    sc.pl.highly_variable_genes(adata_adip)
    sc.pp.pca(adata_adip, n_comps=50, use_highly_variable = True, r
andom_state=0, svd_solver='arpack')
    sc.pp.neighbors(adata_adip, n_neighbors=100, knn=True, method='
umap', n_pcs=50, random_state=0)
    sc.tl.umap(adata_adip)
    if bool_recluster == True:
        sc.tl.louvain(adata_adip, resolution=1, flavor='vtraag', ra
ndom_state=0)
        pd.DataFrame(adata_adip.obs).to_csv(path_or_buf=dir_adata+'
obs_adata_adip.csv')
    else:
        obs = pd.read_csv(dir_adata+'obs_adata_adip.csv')
        adata_adip.obs['louvain']=pd.Series(obs['louvain'].values,
dtype = 'category')
    sc.write(dir_adata+'adata_adip.h5ad',adata_adip)
else:
    adata_adip = sc.read(dir_adata+'adata_adip.h5ad')
sc.tl.paga(adata_adip)
```

```
running PAGA
    finished: added
    'paga/connectivities', connectivities adjacency (adata.uns)
    'paga/connectivities_tree', connectivities subtree (adata.uns)
(0:00:00)
```

In [45]:
```
if bool_plot == True:
    sc.pl.umap(adata_adip, color=['louvain','mt_frac'], size=30, save="_preadip_louvain_0.pdf")
    sc.pl.umap(adata_adip, color=['n_genes','n_counts'], size=30, save="_preadip_louvain_1.pdf")
    sc.pl.paga(adata_adip, color=['louvain','n_counts'], save="_preadip_louvain.pdf")
```

```
WARNING: saving figure to file /Users/david.fischer/phd/data/PreadipocytesBrown/results/panels/umap_preadip_louvain_0.pdf
```

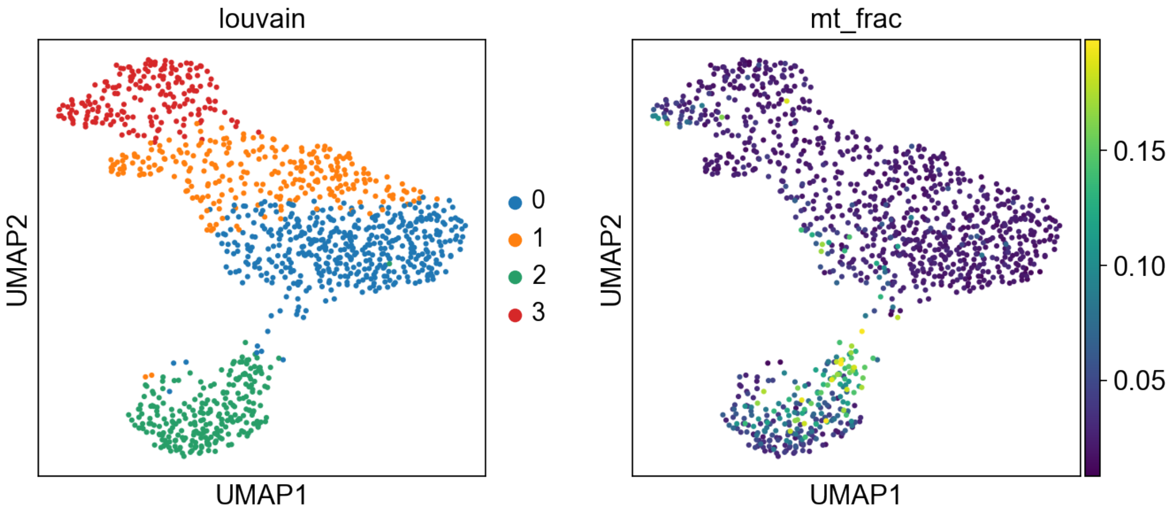


```
WARNING: saving figure to file /Users/david.fischer/phd/data/PreadipocytesBrown/results/panels/umap_preadip_louvain_1.pdf
```

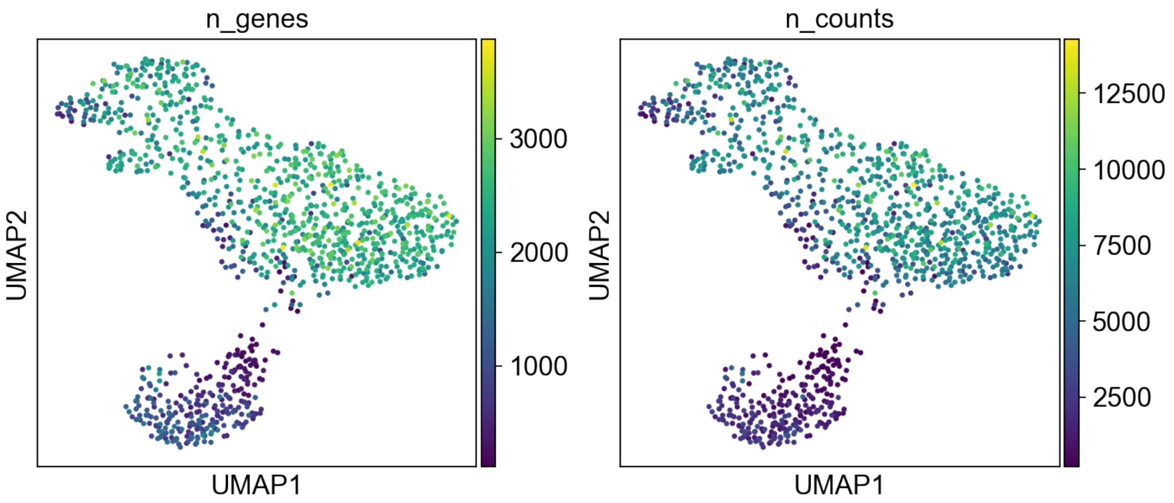


```
--> added 'pos', the PAGA positions (adata.uns['paga'])
WARNING: saving figure to file /Users/david.fischer/phd/data/PreadipocytesBrown/results/panels/paga_preadip_louvain.pdf
```

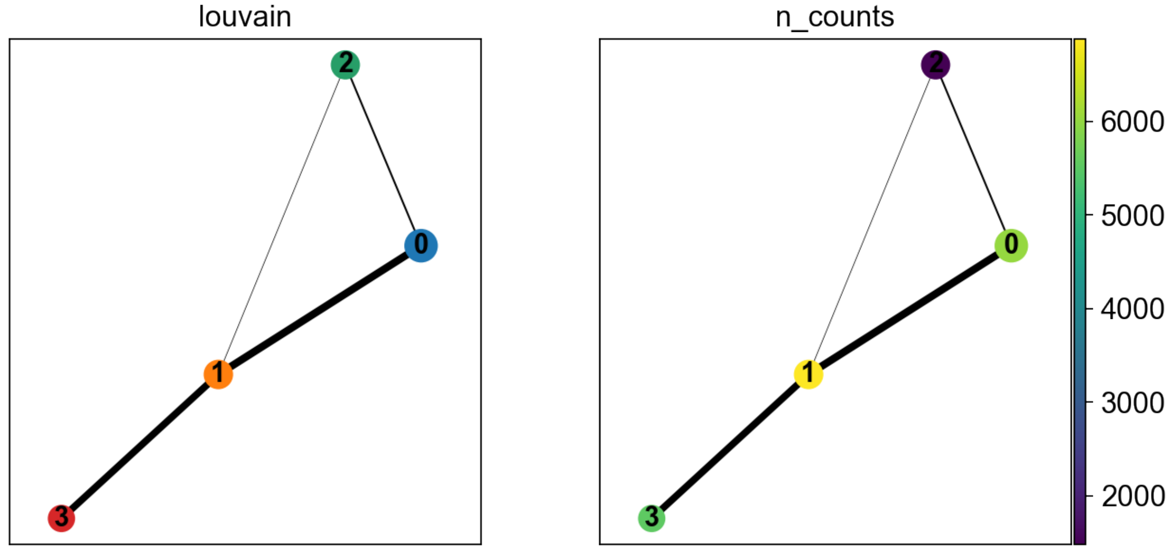

Cluster 0,1,3 (top) shows many more counts than cluster 2 (bottom).

Adipocyte Marker Analysis

In [46]:
```
if bool_plot == True:
    plot_violin_marker(adata_adip, adipocyte_markers.tolist(), save
="_preadip_markers_preadipcytes", use_raw=False)
```

```
WARNING: saving figure to file /Users/david.fischer/phd/data/Pread
ipocytesBrown/results/panels/violin_preadip_markers_preadipcytes_0
.pdf
```

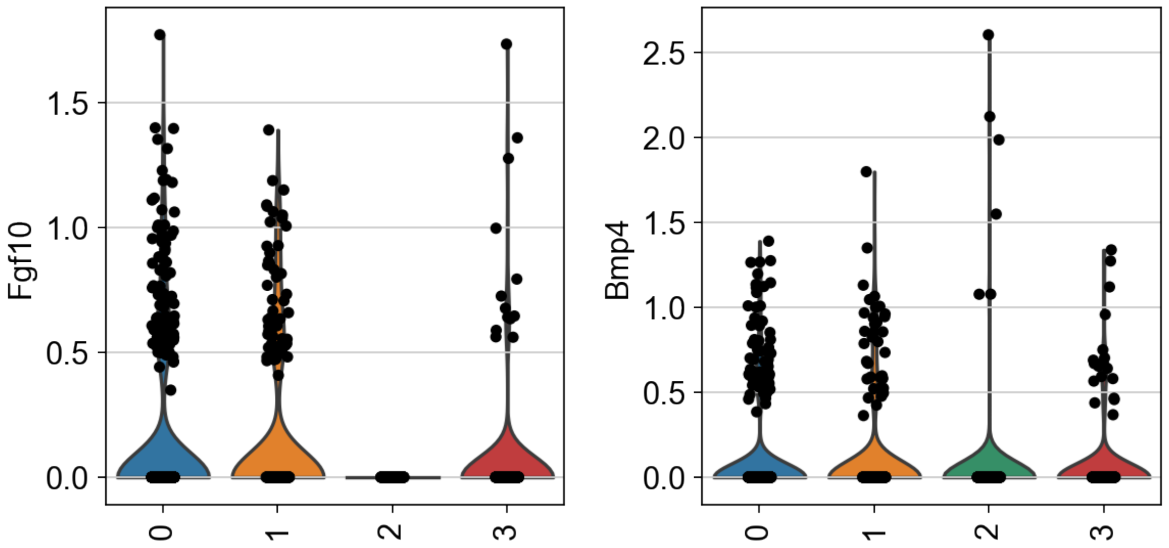

```
WARNING: saving figure to file /Users/david.fischer/phd/data/Pread
ipocytesBrown/results/panels/violin_preadip_markers_preadipcytes_1
.pdf
```

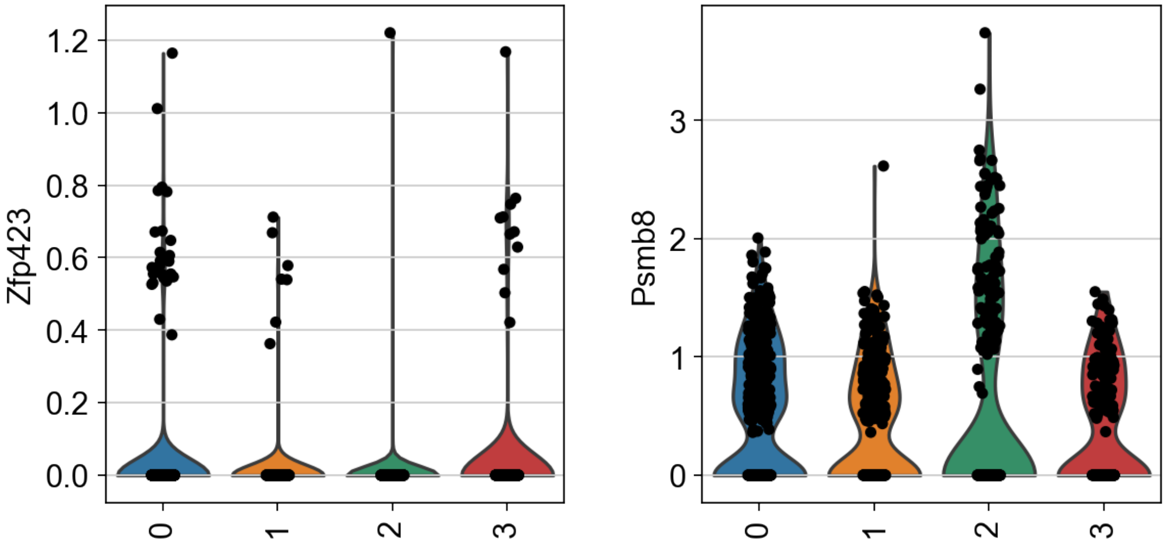


```
WARNING: saving figure to file /Users/david.fischer/phd/data/Pread
ipocytesBrown/results/panels/violin_preadip_markers_preadipcytes_2
.pdf
```

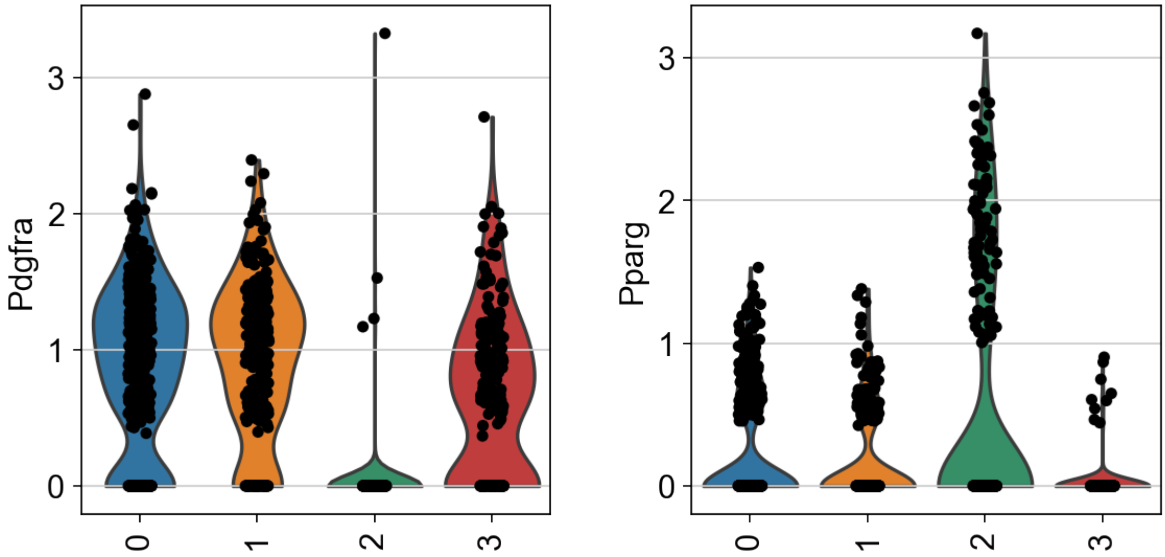


```
WARNING: saving figure to file /Users/david.fischer/phd/data/Pread
ipocytesBrown/results/panels/violin_preadip_markers_preadipcytes_3
.pdf
```

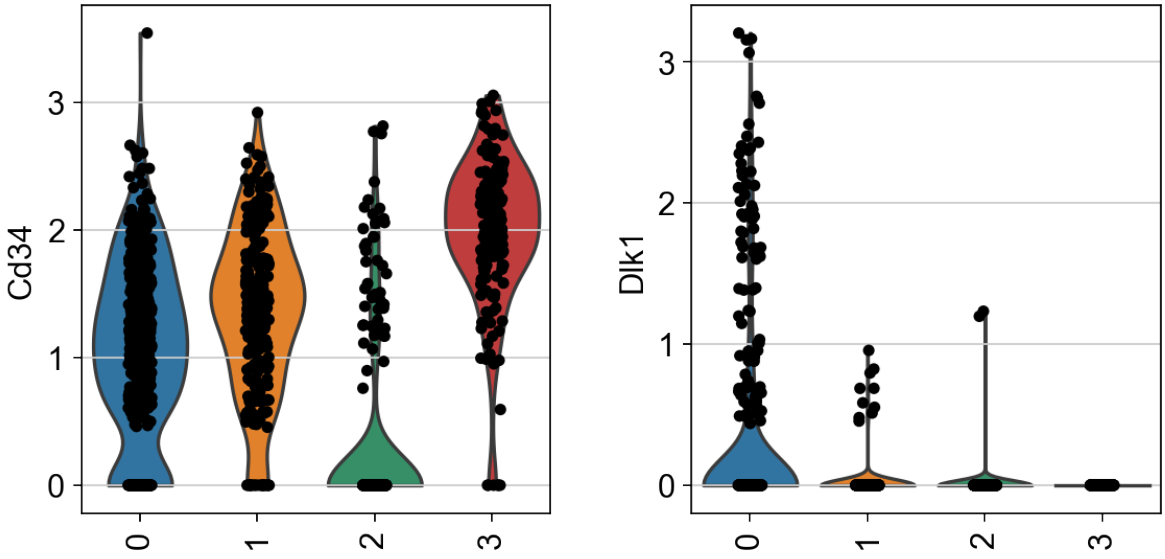


```
WARNING: saving figure to file /Users/david.fischer/phd/data/Pread
ipocytesBrown/results/panels/violin_preadip_markers_preadipcytes_4
.pdf
```

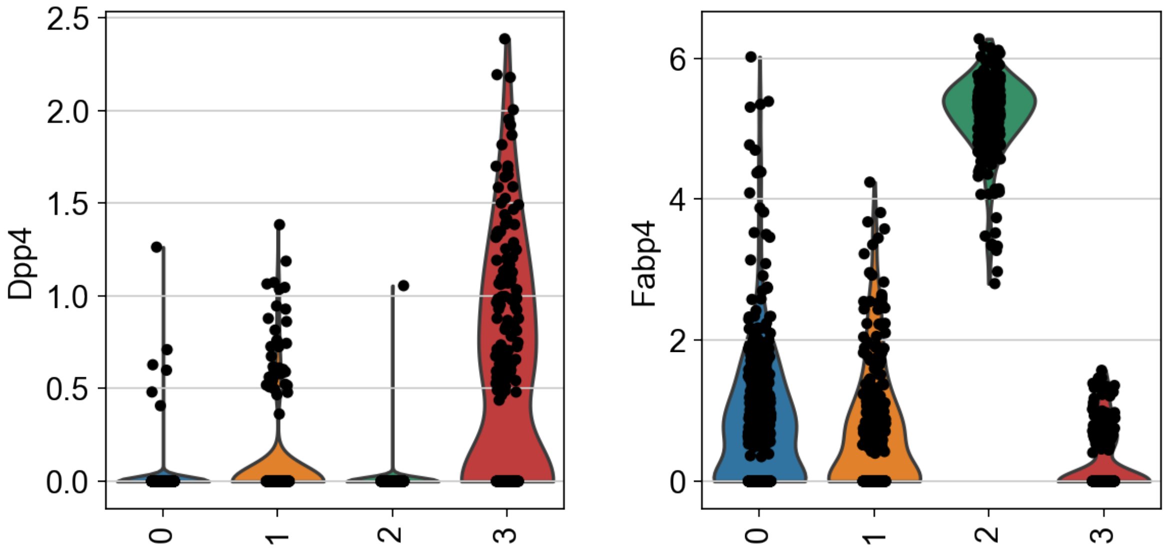


```
WARNING: saving figure to file /Users/david.fischer/phd/data/Pread
ipocytesBrown/results/panels/violin_preadip_markers_preadipcytes_5
.pdf
```

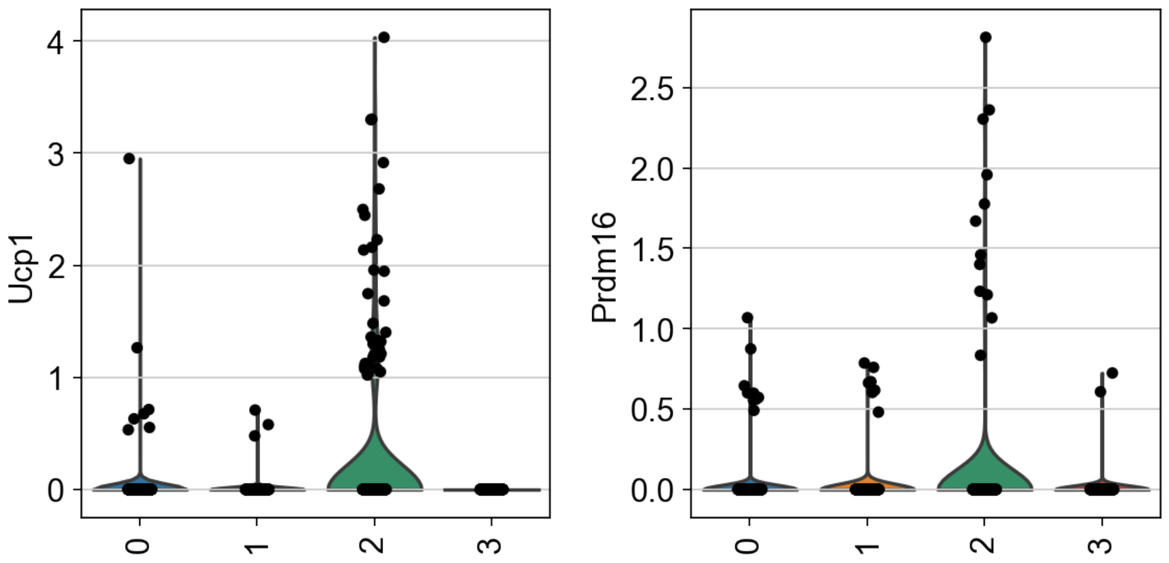

WARNING: saving figure to file /Users/david.fischer/phd/data/PreadipocytesBrown/results/panels/violin_preadip_markers_preadipcytes_6.pdf

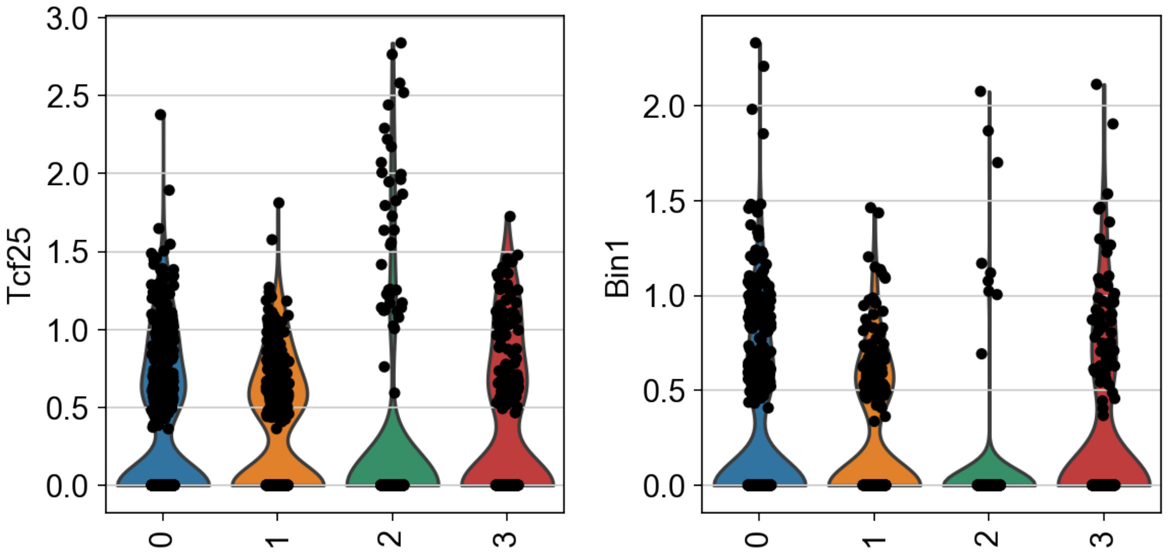

WARNING: saving figure to file /Users/david.fischer/phd/data/PreadipocytesBrown/results/panels/violin_preadip_markers_preadipcytes_7.pdf

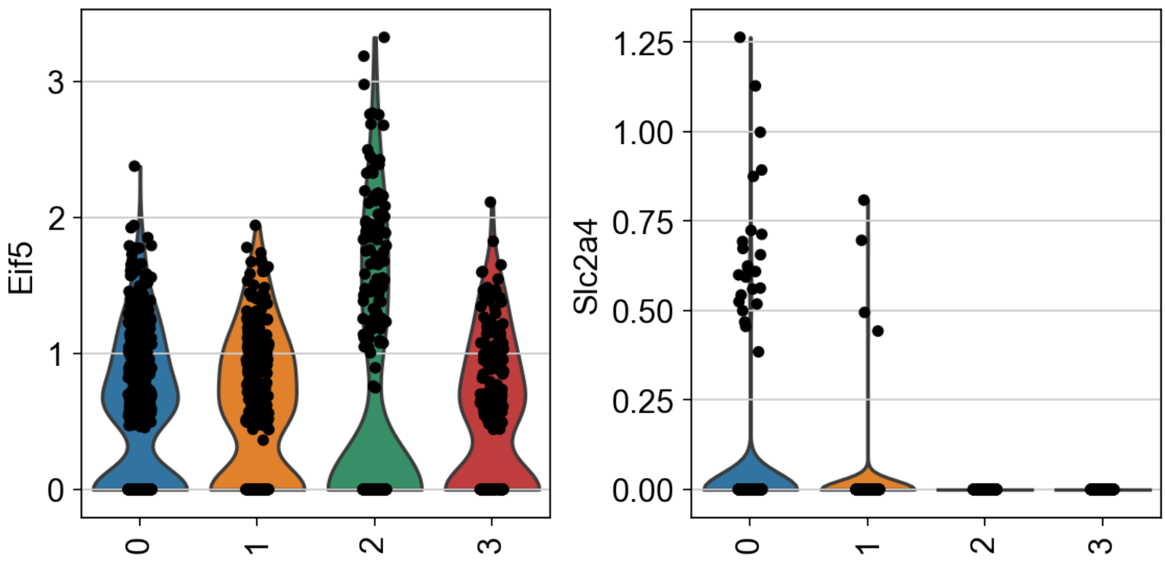

WARNING: saving figure to file /Users/david.fischer/phd/data/PreadipocytesBrown/results/panels/violin_preadip_markers_preadipcytes_8.pdf

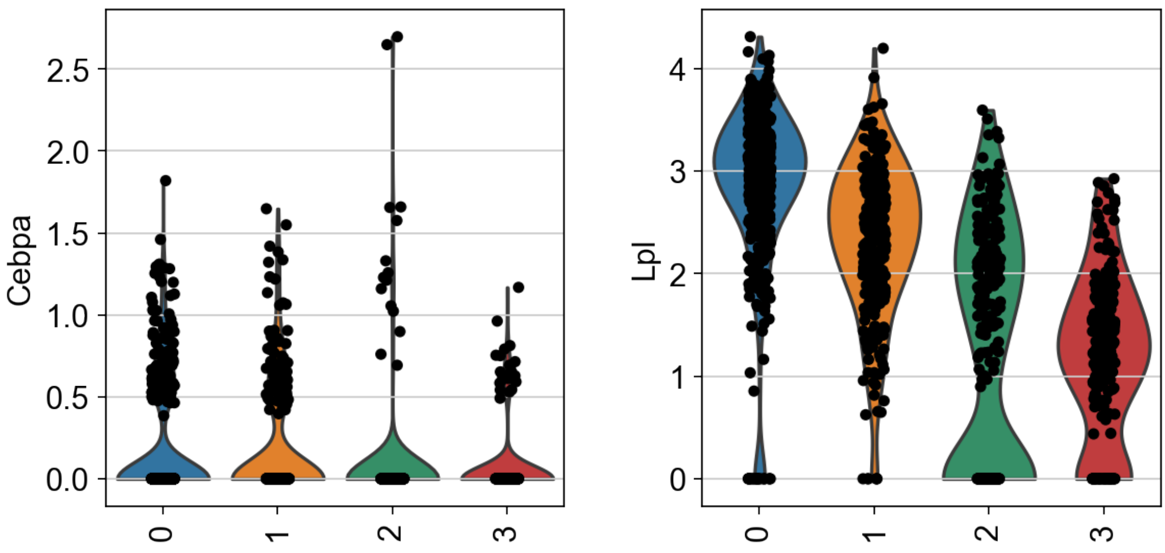

WARNING: saving figure to file /Users/david.fischer/phd/data/PreadipocytesBrown/results/panels/violin_preadip_markers_preadipcytes_9.pdf

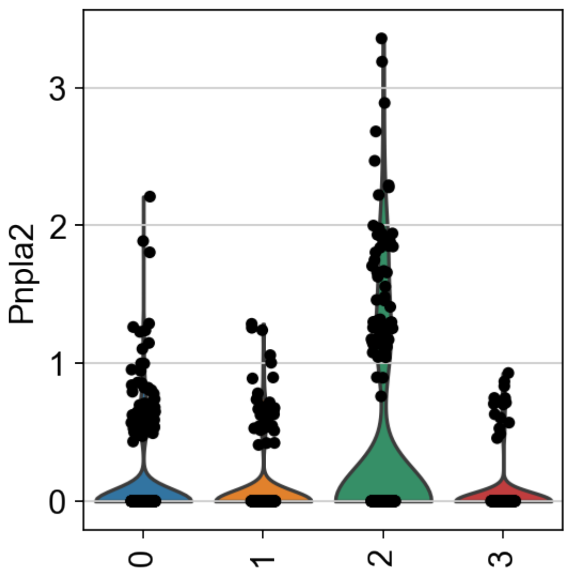

FABP4 is present only in cluster 2. Together with Pparg expression in cluster 2, it can be concluded that cluster 2 represents mature adipocytes instead of preadipocytes. Clusters 0,1 and 3 express multiple preadpocyte markers.

In [47]:
```
if bool_plot==True:
    sc.pl.heatmap(
        adata=adata_adip,
        var_names=adipocyte_markers,
        groupby="louvain",
        use_raw=False,
        log=False,
        dendrogram=True,
        var_group_rotation=90,
        show_gene_labels=True,
        show=True,
        save="_adipocyte_markers_celltypes.pdf"
    )
```

```
WARNING: dendrogram data not found (using key=dendrogram_louvain).
Running `sc.tl.dendrogram` with default parameters. For fine tunin
g it is recommended to run `sc.tl.dendrogram` independently.
    using 'X_pca' with n_pcs = 50
Storing dendrogram info using `.uns['dendrogram_louvain']`
WARNING: saving figure to file /Users/david.fischer/phd/data/Pread
ipocytesBrown/results/panels/heatmap_adipocyte_markers_celltypes.p
df
```

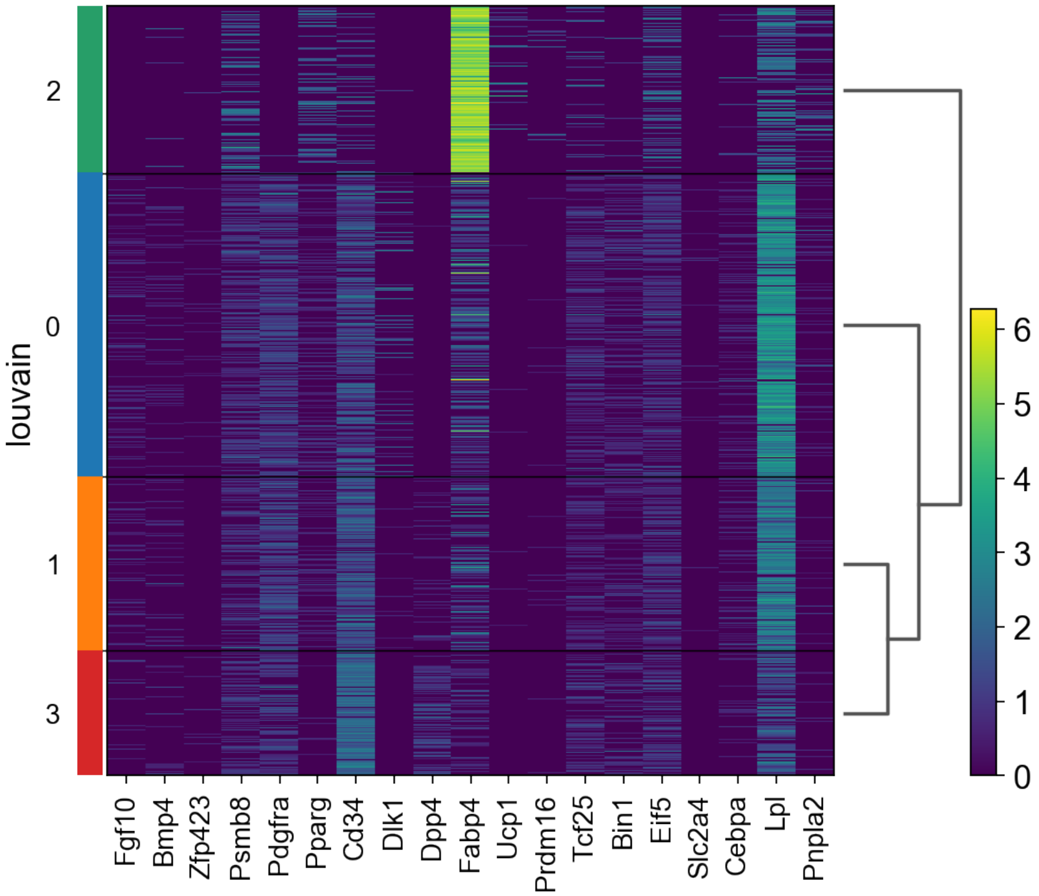

Top ranking DE Genes

In [48]:
```
sc.tl.rank_genes_groups(adata_adip, groupby='louvain', key_added='r
ank_genes')
if bool_plot == True:
    sc.pl.rank_genes_groups(adata_adip, key='rank_genes', groups=['
0','1','2','3'], fontsize=12, save="_adip_genes")
```

```
ranking genes
    finished: added to `.uns['rank_genes']`
    'names', sorted np.recarray to be indexed by group ids
    'scores', sorted np.recarray to be indexed by group ids
    'logfoldchanges', sorted np.recarray to be indexed by group id
s
    'pvals', sorted np.recarray to be indexed by group ids
    'pvals_adj', sorted np.recarray to be indexed by group ids (0:
00:00)
WARNING: saving figure to file /Users/david.fischer/phd/data/Pread
ipocytesBrown/results/panels/rank_genes_groups_louvain_adip_genes.
pdf
```

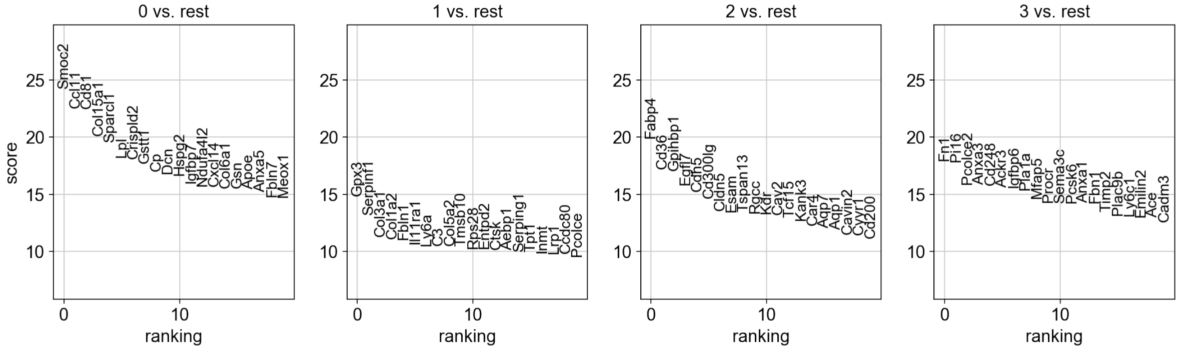

Marker Counts Raw and Normalized

In [49]:
```
if bool_plot == True:
    plot_umap_marker(adata_adip, adipocyte_markers.tolist(), size=5
0, save="_preadip_markers_preadipcytes_raw", use_raw=True)
```

```
['Fgf10', 'Bmp4']
WARNING: saving figure to file /Users/david.fischer/phd/data/Pread
ipocytesBrown/results/panels/umap_preadip_markers_preadipcytes_raw
_0.pdf
```

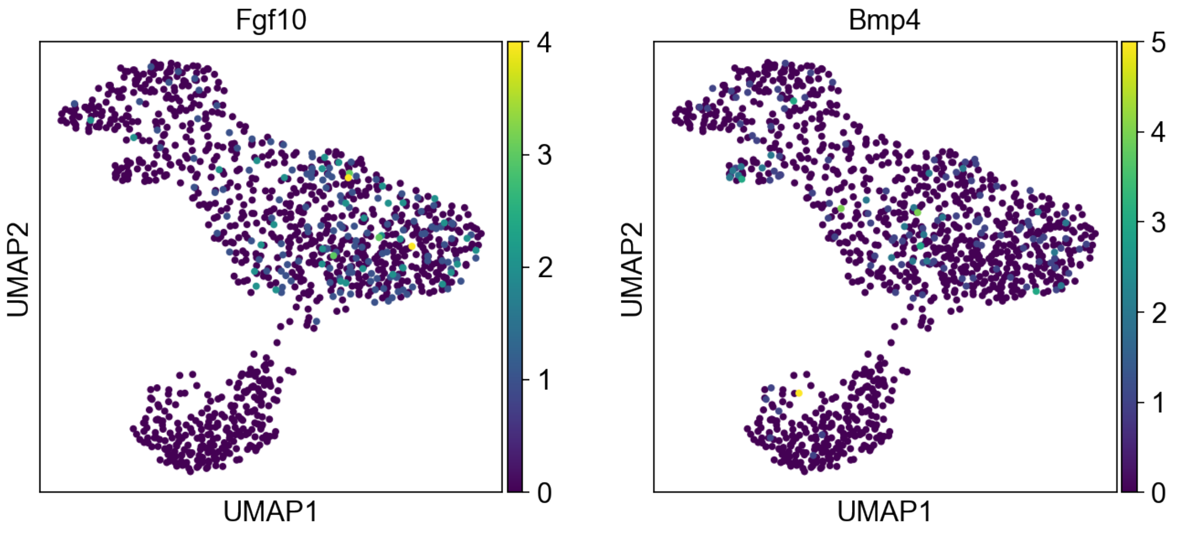


```
['Zfp423', 'Psmb8']
WARNING: saving figure to file /Users/david.fischer/phd/data/Pread
ipocytesBrown/results/panels/umap_preadip_markers_preadipcytes_raw
_1.pdf
```

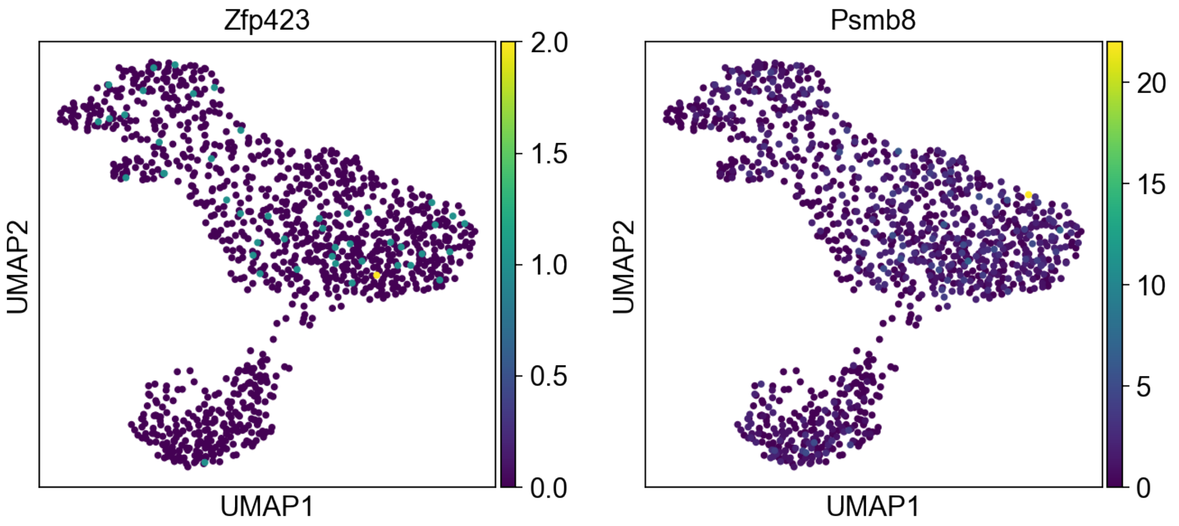


```
['Pdgfra', 'Pparg']
WARNING: saving figure to file /Users/david.fischer/phd/data/Pread
ipocytesBrown/results/panels/umap_preadip_markers_preadipcytes_raw
_2.pdf
```

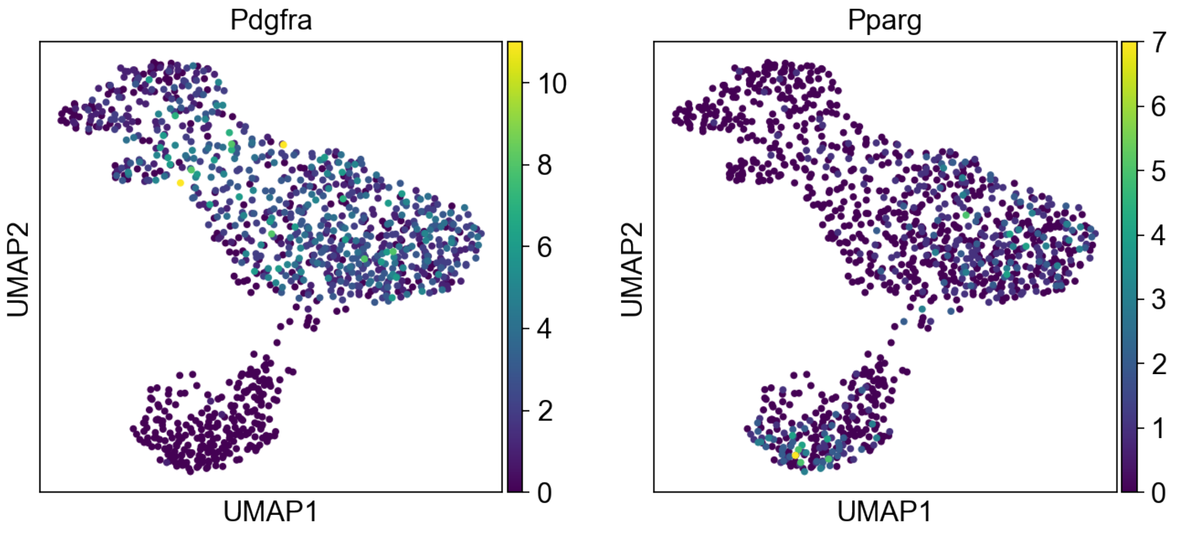


```
['Cd34', 'Dlk1']
WARNING: saving figure to file /Users/david.fischer/phd/data/Pread
ipocytesBrown/results/panels/umap_preadip_markers_preadipcytes_raw
_3.pdf
```

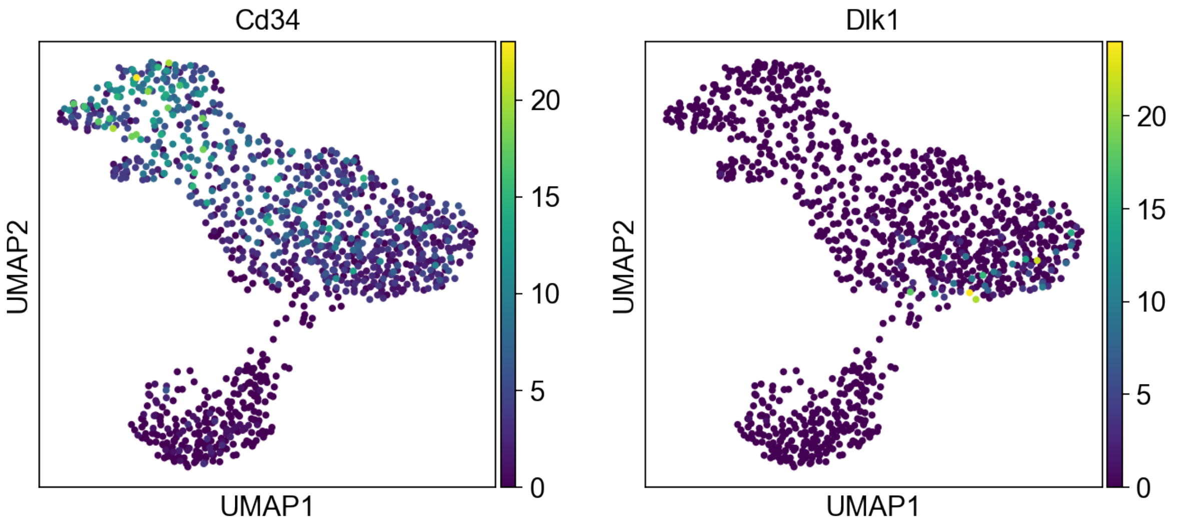


```
['Dpp4', 'Fabp4']
WARNING: saving figure to file /Users/david.fischer/phd/data/Pread
ipocytesBrown/results/panels/umap_preadip_markers_preadipcytes_raw
_4.pdf
```

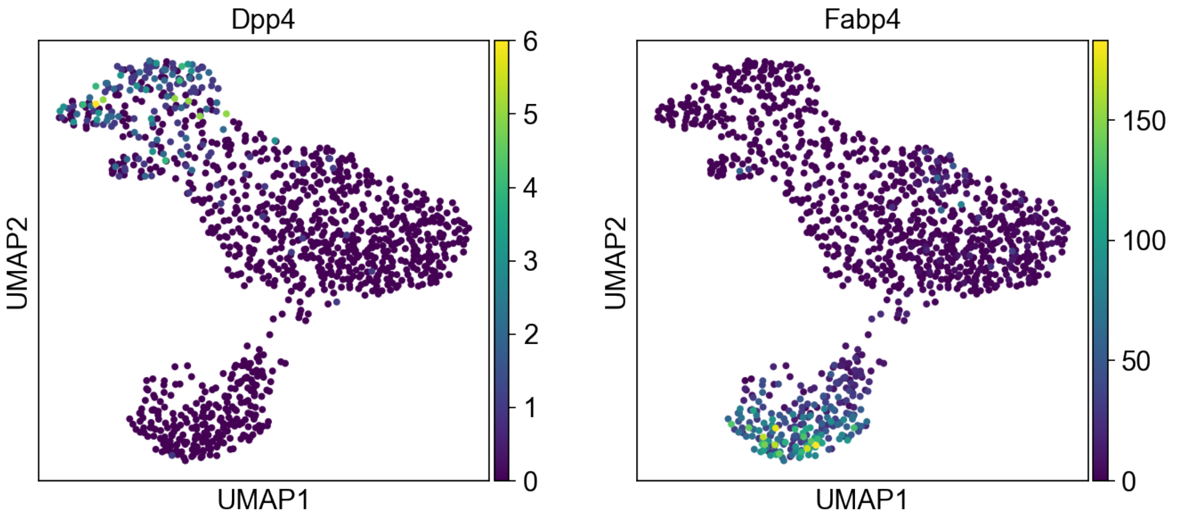


```
['Ucp1', 'Prdm16']
WARNING: saving figure to file /Users/david.fischer/phd/data/Preadipoc
ipocytesBrown/results/panels/umap_preadip_markers_preadipcytes_raw
_5.pdf
```

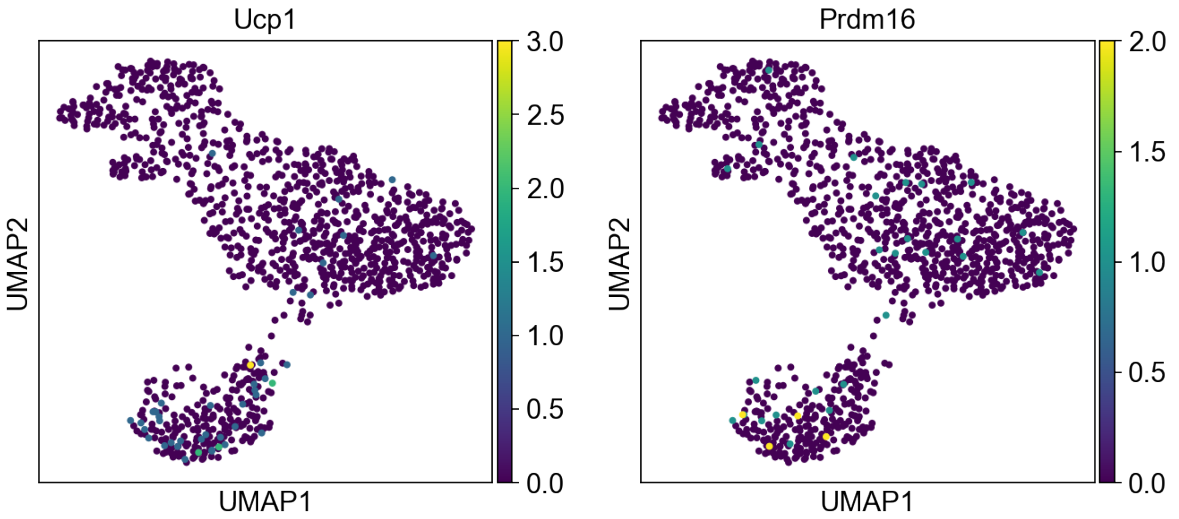


```
['Tcf25', 'Bin1']
WARNING: saving figure to file /Users/david.fischer/phd/data/Pread
ipocytesBrown/results/panels/umap_preadip_markers_preadipcytes_raw
_6.pdf
```

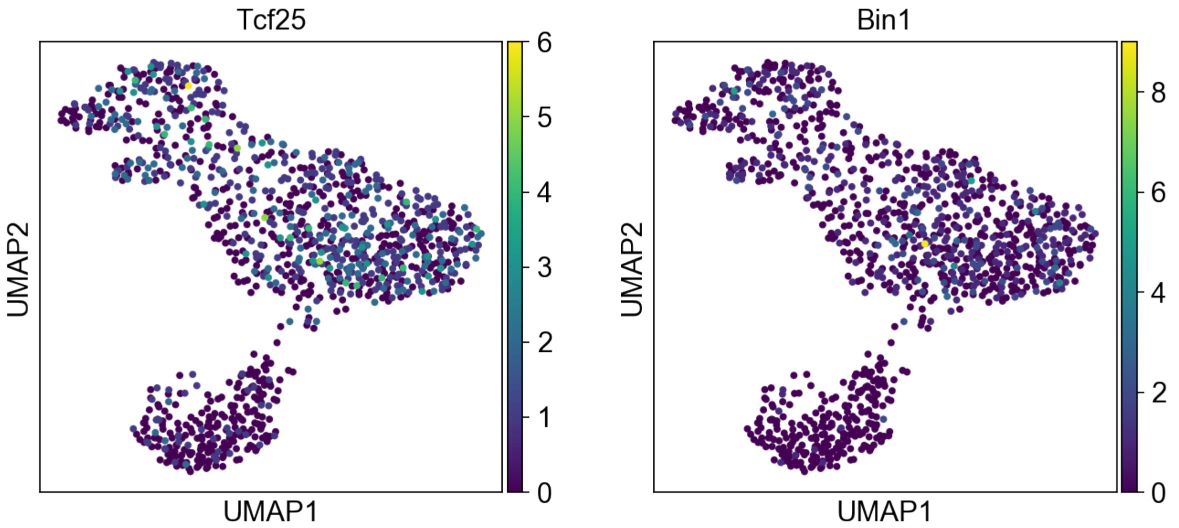


```
['Eif5', 'Slc2a4']
WARNING: saving figure to file /Users/david.fischer/phd/data/Preadipocyt
esBrown/results/panels/umap_preadip_markers_preadipcytes_raw_7.pdf
```

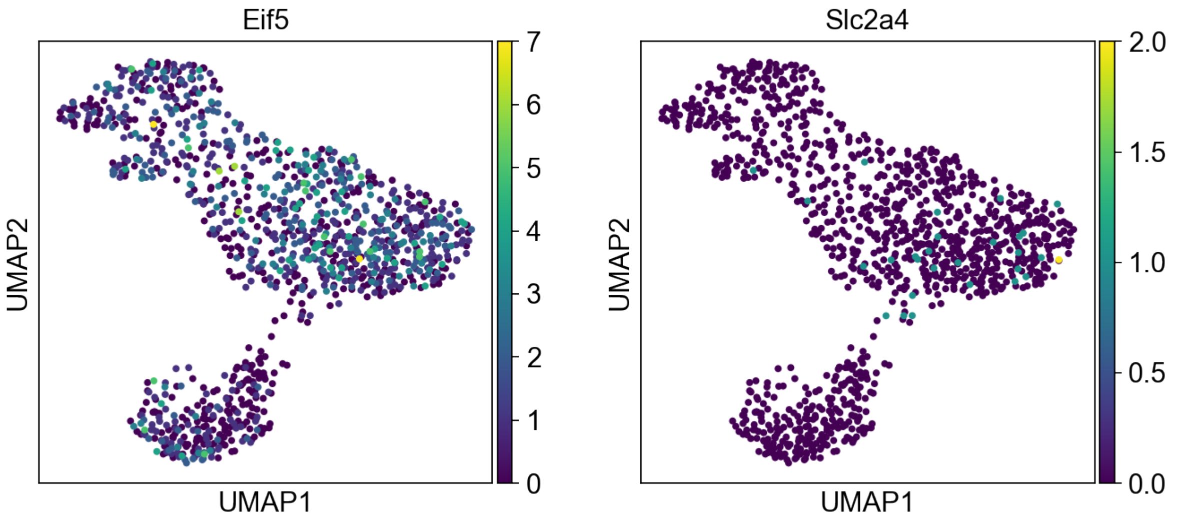


```
['Cebpa', 'Lpl']
WARNING: saving figure to file /Users/david.fischer/phd/data/Preadipocyt
esBrown/results/panels/umap_preadip_markers_preadipcytes_raw_8.pdf
```

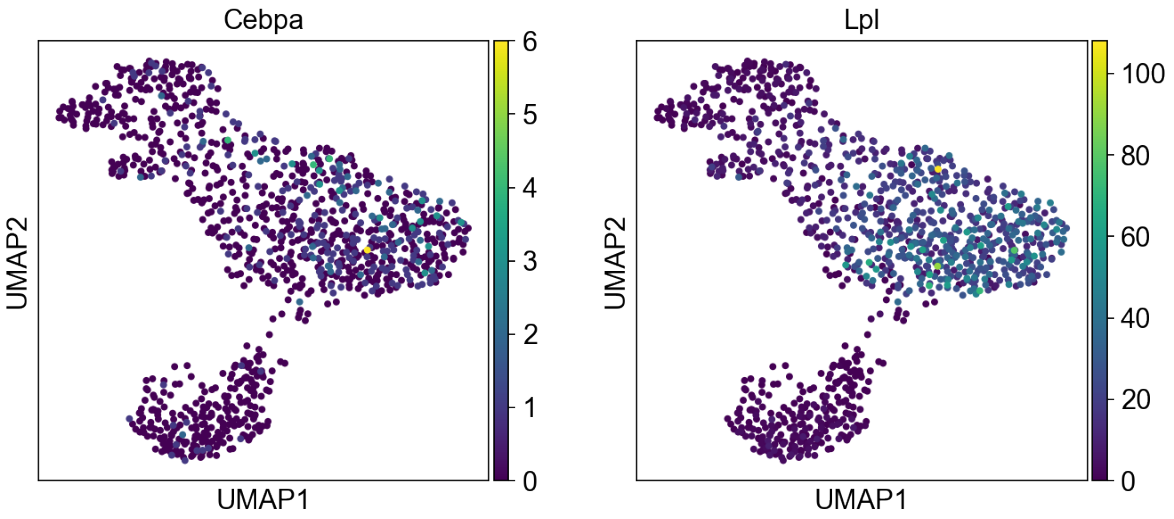


```
['Pnpla2']
WARNING: saving figure to file /Users/david.fischer/phd/data/Pread
ipocytesBrown/results/panels/umap_preadip_markers_preadipcytes_raw
_9.pdf
```

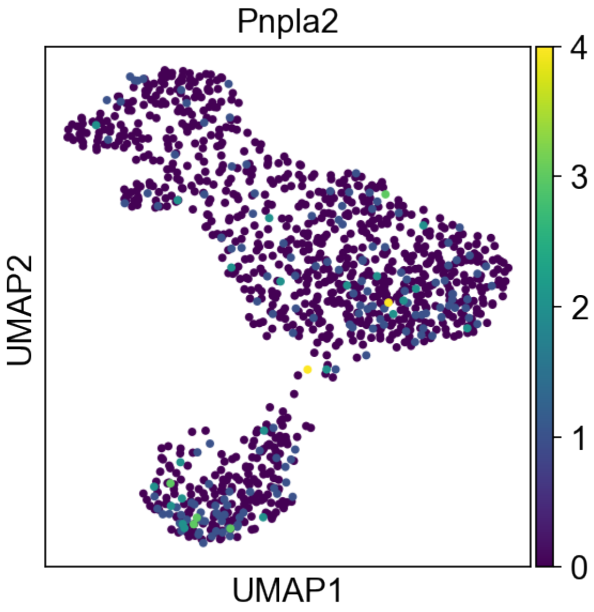

In [50]:
```
if bool_plot == True:
    plot_umap_marker(adata_adip, adipocyte_markers.tolist(), size=5
0, save="_preadip_markers_preadipcytes_norm", use_raw=False)
```

```
['Fgf10', 'Bmp4']
WARNING: saving figure to file /Users/david.fischer/phd/data/Pread
ipocytesBrown/results/panels/umap_preadip_markers_preadipcytes_nor
m_0.pdf
```

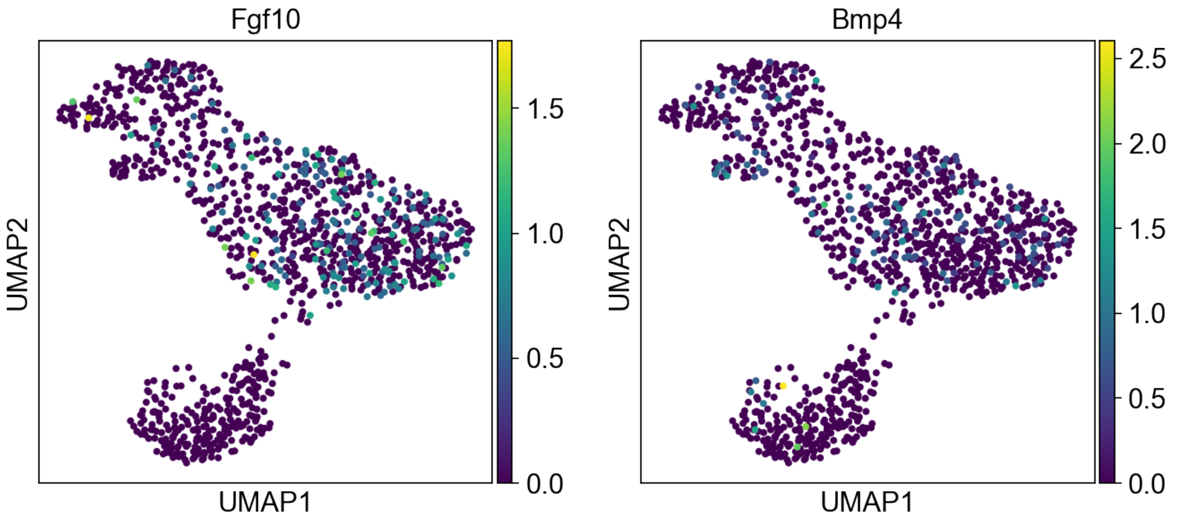


```
['Zfp423', 'Psmb8']
WARNING: saving figure to file /Users/david.fischer/phd/data/Pread
ipocytesBrown/results/panels/umap_preadip_markers_preadipcytes_nor
m_1.pdf
```

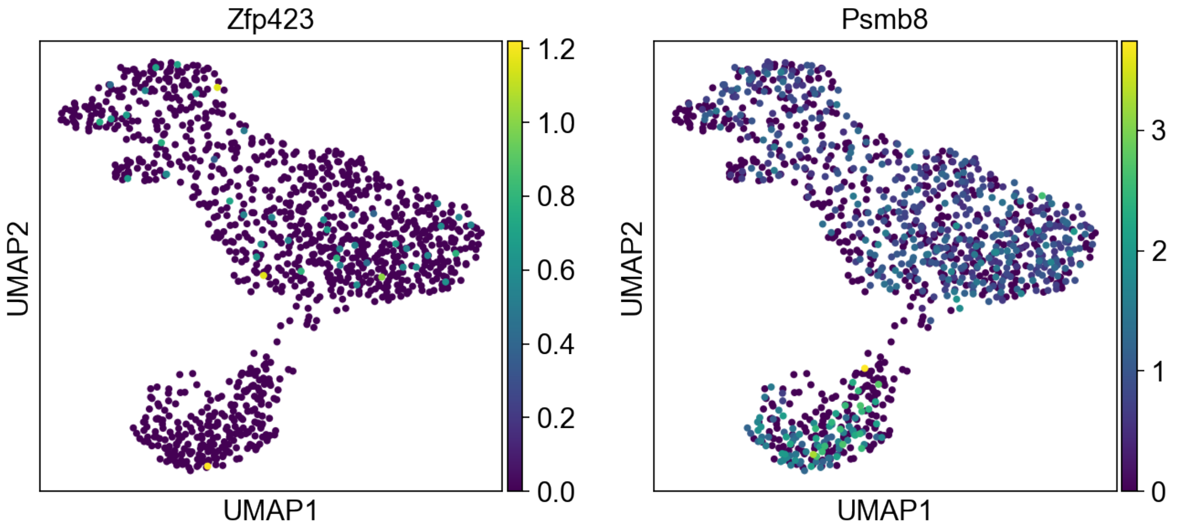


```
['Pdgfra', 'Pparg']
WARNING: saving figure to file /Users/david.fischer/phd/data/Pread
ipocytesBrown/results/panels/umap_preadip_markers_preadipcytes_nor
m_2.pdf
```

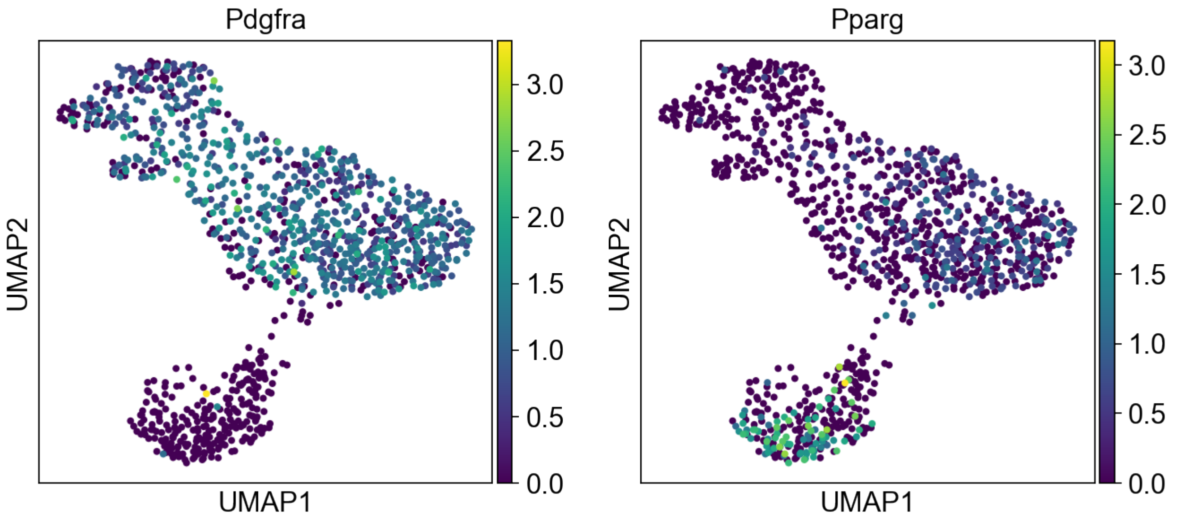


```
['Cd34', 'Dlk1']
WARNING: saving figure to file /Users/david.fischer/phd/data/Pread
ipocytesBrown/results/panels/umap_preadip_markers_preadipcytes_nor
m_3.pdf
```

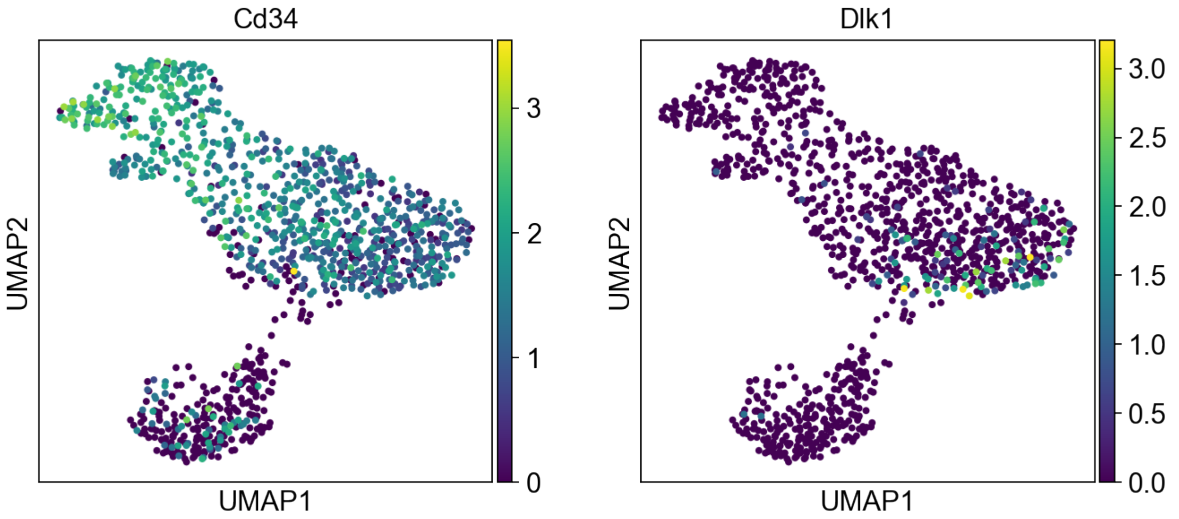


```
['Dpp4', 'Fabp4']
WARNING: saving figure to file /Users/david.fischer/phd/data/Pread
ipocytesBrown/results/panels/umap_preadip_markers_preadipcytes_nor
m_4.pdf
```

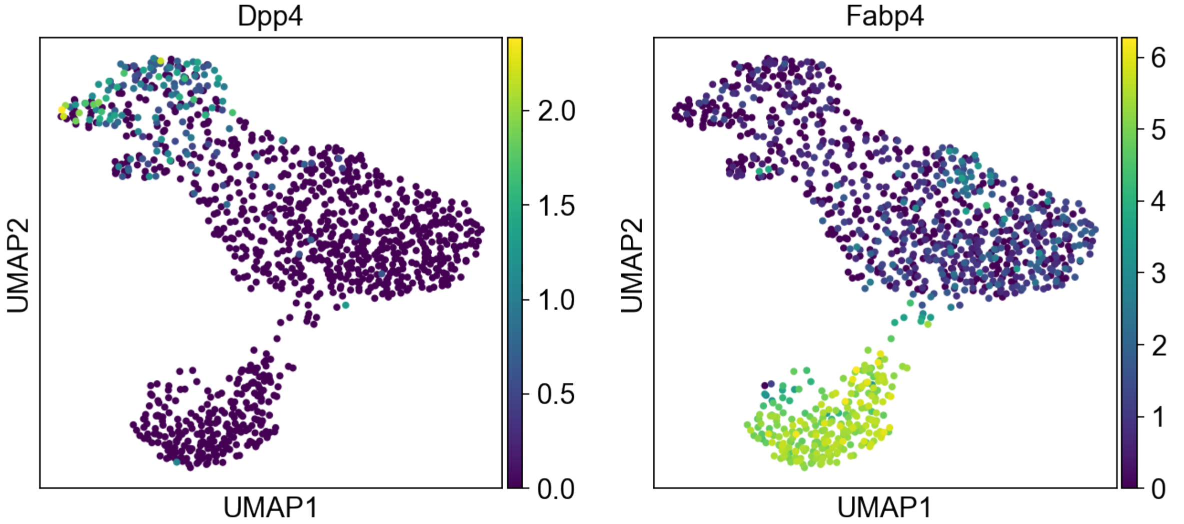


```
['Ucp1', 'Prdm16']
WARNING: saving figure to file /Users/david.fischer/phd/data/Pread
ipocytesBrown/results/panels/umap_preadip_markers_preadipcytes_nor
m_5.pdf
```

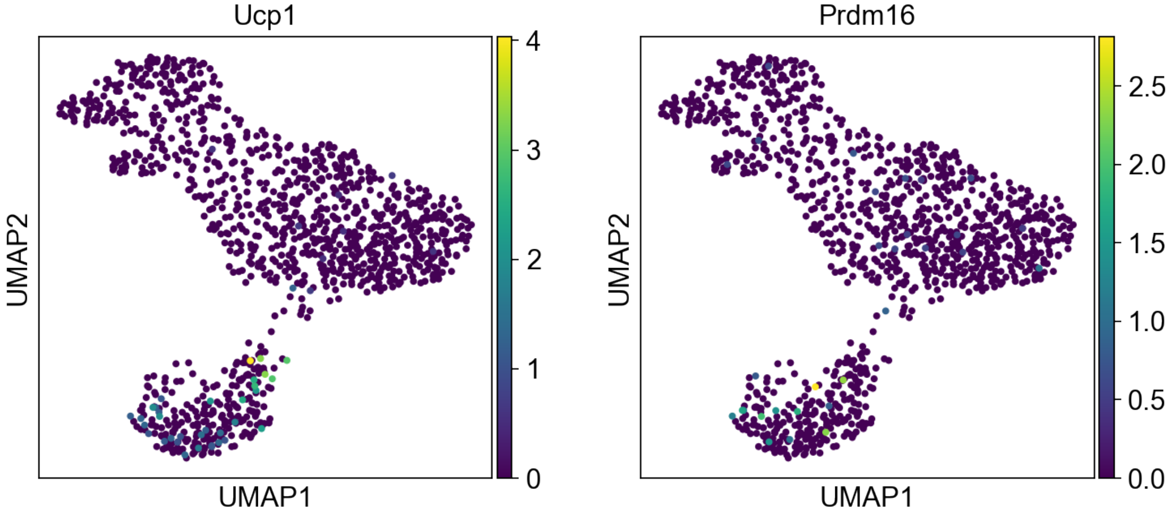


```
['Tcf25', 'Bin1']
WARNING: saving figure to file /Users/david.fischer/phd/data/Pread
ipocytesBrown/results/panels/umap_preadip_markers_preadipcytes_nor
m_6.pdf
```

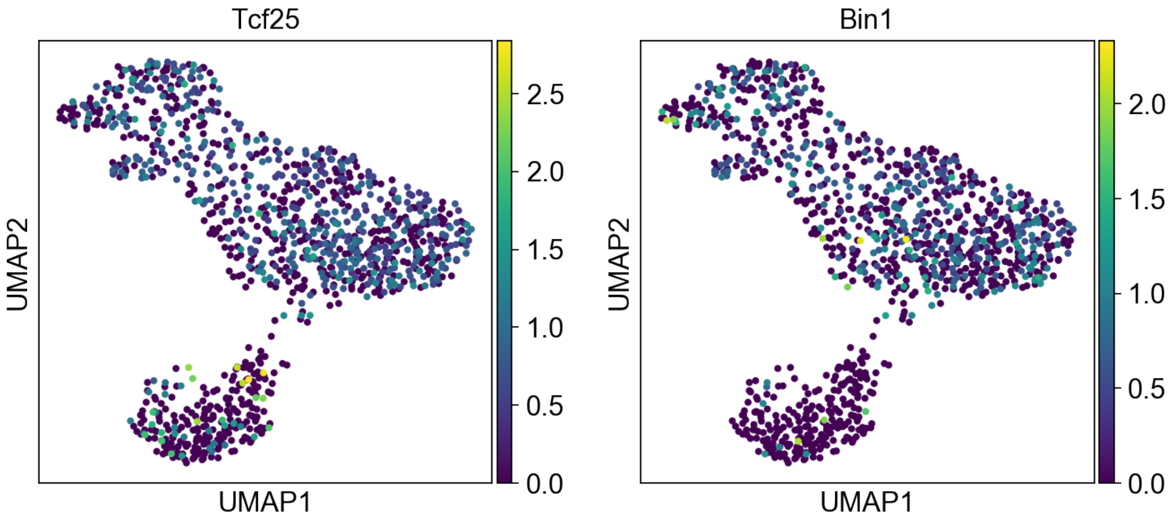


```
['Eif5', 'Slc2a4']
WARNING: saving figure to file /Users/david.fischer/phd/data/Pread
ipocytesBrown/results/panels/umap_preadip_markers_preadipcytes_nor
m_7.pdf
```

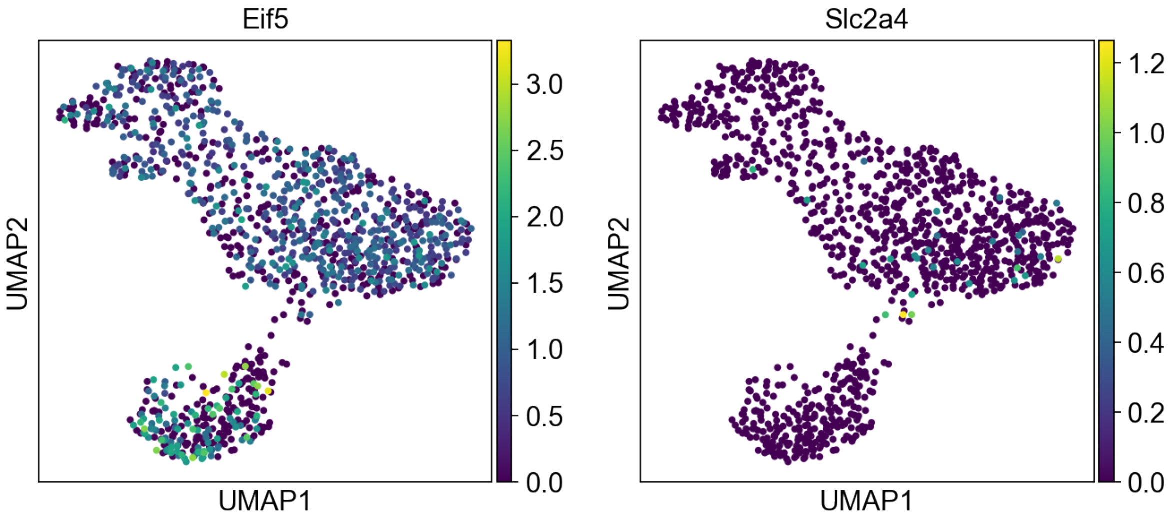


```
['Cebpa', 'Lpl']
WARNING: saving figure to file /Users/david.fischer/phd/data/Pread
ipocytesBrown/results/panels/umap_preadip_markers_preadipcytes_nor
m_8.pdf
```

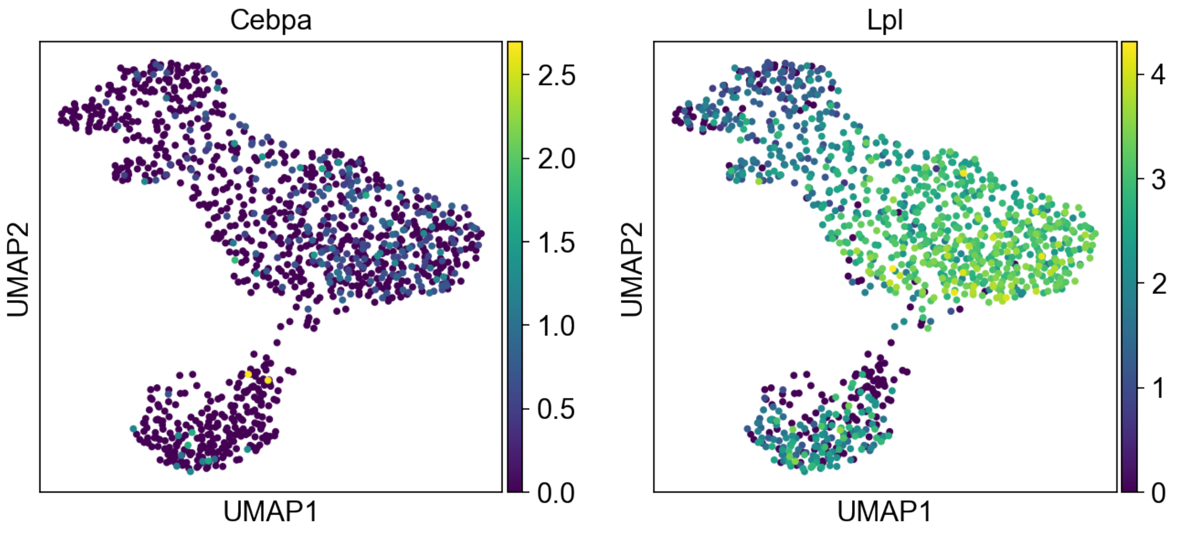


```
['Pnpla2']
WARNING: saving figure to file /Users/david.fischer/phd/data/Pread
ipocytesBrown/results/panels/umap_preadip_markers_preadipcytes_nor
m_9.pdf
```

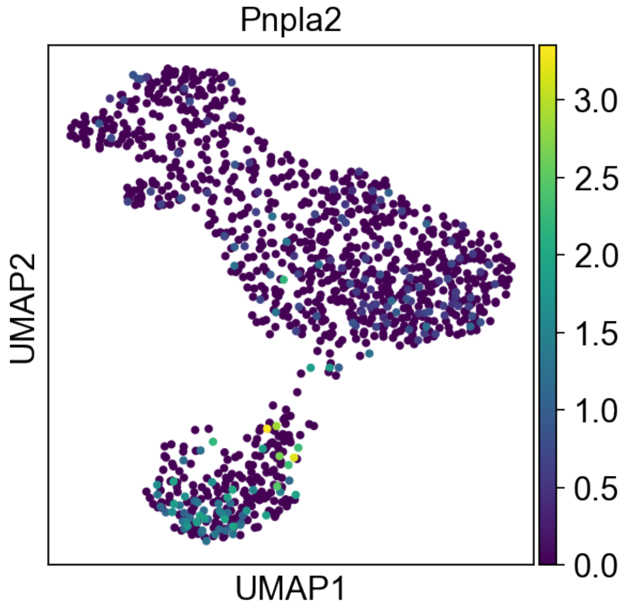

Count distribution for Tcf25, Bin1 and Eif5

Single Double and Triple Positive Counts

Define booleans for single, double and triple positive counts of bin1, eif5 and tcf25

In [54]:
```
non_boolean_int = np.array((adata_adip[:,'Bin1'].X.todense()<=0) &
(adata_adip[:,'Eif5'].X.todense()<=0) & (adata_adip[:,'Tcf25'].X.to
dense()<=0), dtype=int)

bin1_single_boolean = (adata_adip[:,'Bin1'].X.todense()>0) & (adata
_adip[:,'Eif5'].X.todense()<=0) & (adata_adip[:,'Tcf25'].X.todense(
)<=0)
eif5_single_boolean = (adata_adip[:,'Eif5'].X.todense()>0) & (adata
_adip[:,'Bin1'].X.todense()<=0) & (adata_adip[:,'Tcf25'].X.todense(
)<=0)
tcf25_single_boolean = (adata_adip[:,'Tcf25'].X.todense()>0) & (ada
ta_adip[:,'Eif5'].X.todense()<=0) & (adata_adip[:,'Bin1'].X.todense
()<=0)
single_boolean_int = np.array((bin1_single_boolean | eif5_single_bo
olean | tcf25_single_boolean), dtype=int)*1

bin1_eif5_double_boolean = (adata_adip[:,'Bin1'].X.todense()>0) & (
adata_adip[:,'Eif5'].X.todense()>0) & (adata_adip[:,'Tcf25'].X.tode
nse()<=0)
bin1_tcf25_double_boolean = (adata_adip[:,'Eif5'].X.todense()<=0) &
(adata_adip[:,'Bin1'].X.todense()>0) & (adata_adip[:,'Tcf25'].X.tod
ense()>0)
tcf25_eif5_double_boolean = (adata_adip[:,'Tcf25'].X.todense()>0) &
(adata_adip[:,'Eif5'].X.todense()>0) & (adata_adip[:,'Bin1'].X.tode
nse()<=0)
double_boolean_int = np.array((bin1_eif5_double_boolean | bin1_tcf2
5_double_boolean | tcf25_eif5_double_boolean), dtype=int)*2

triple_boolean = (adata_adip[:,'Tcf25'].X.todense()>0) & (adata_adi
p[:,'Eif5'].X.todense()>0) & (adata_adip[:,'Bin1'].X.todense()>0)
triple_boolean_int = np.array(triple_boolean, dtype=int)*3
non_boolean_int *=0
```

In [55]:
```
bin1_single_pos = adata_adip[:,'Bin1'].X[bin1_single_boolean]
eif5_single_pos = adata_adip[:,'Eif5'].X[eif5_single_boolean]
tcf25_single_pos = adata_adip[:,'Tcf25'].X[tcf25_single_boolean]

print('Bin1 Single Positive ',len(bin1_single_pos))
print('eif5 Single Positive ',len(eif5_single_pos))
print('tcf25 Single Positive ',len(tcf25_single_pos))

bin1_eif5_double_pos = adata_adip[:,'Bin1'].X[bin1_eif5_double_bool
ean]
bin1_tcf25_double_pos = adata_adip[:,'Eif5'].X[bin1_tcf25_double_bo
olean]
tcf25_eif5_double_pos = adata_adip[:,'Tcf25'].X[tcf25_eif5_double_b
oolean]

print('Bin1/Eif5 Double Positive ',len(bin1_eif5_double_pos))
print('Bin1/Tcf25 Double Positive ',len(bin1_tcf25_double_pos))
print('Tcf25/Eif5 Double Positive ',len(tcf25_eif5_double_pos))

triple_pos = adata_adip[:,'Bin1'].X[triple_boolean]
print('Triple Positive ',len(triple_pos))
```

```
Bin1 Single Positive  1
eif5 Single Positive  1
tcf25 Single Positive  1
Bin1/Eif5 Double Positive  1
Bin1/Tcf25 Double Positive  1
Tcf25/Eif5 Double Positive  1
Triple Positive  1
```

Define observations for single, double and triple positive counts of bin1, eif5 and tcf25

In [56]:
```
adata_adip.obs['sdt_pos'] = np.array((non_boolean_int + single_bool
ean_int + double_boolean_int + triple_boolean_int), dtype=str)
adata_adip.obs['s_pos'] = np.array((np.array(bin1_single_boolean, d
type=int)*1)+(np.array(eif5_single_boolean, dtype=int)*2)+(np.array
(tcf25_single_boolean, dtype=int)*3), dtype=str)
adata_adip.obs['d_pos'] = np.array((np.array(bin1_eif5_double_boole
an, dtype=int)*1)+(np.array(bin1_tcf25_double_boolean, dtype=int)*2
)+(np.array(tcf25_eif5_double_boolean, dtype=int)*3), dtype=str)
adata_adip.obs['t_pos'] = np.array(triple_boolean, dtype=str)
# make them categorical
adata_adip.obs['sdt_pos'] = pd.Series(adata_adip.obs['sdt_pos'], dt
ype="category")
adata_adip.obs['s_pos'] = pd.Series(adata_adip.obs['s_pos'], dtype=
"category")
adata_adip.obs['d_pos'] = pd.Series(adata_adip.obs['d_pos'], dtype=
"category")
adata_adip.obs['t_pos'] = pd.Series(adata_adip.obs['t_pos'], dtype=
"category")
```

In [57]:
```
if bool_plot == True:
    new_cluster_names = ['Non Positives','Single Positives','Double
Positives','Triple Positives']
    adata_adip.rename_categories('sdt_pos', new_cluster_names)
    sc.pl.umap(adata_adip, color=['sdt_pos'], size=20)
    new_cluster_names = ['Non Single Positives','Bin1 Single Positi
ves','Eif5 Single Positives','Tcf25 Single Positives']
    adata_adip.rename_categories('s_pos', new_cluster_names)
    sc.pl.umap(adata_adip[adata_adip.obs['s_pos']!='Non Single Posi
tives'], color=['s_pos'], size=20)
    new_cluster_names = ['Non Double Positives','Bin1/Eif5 Double P
ositive','Bin1/Tcf25 Double Positive','Tcf25/Eif5 Double Positive']
    adata_adip.rename_categories('d_pos', new_cluster_names)
    sc.pl.umap(adata_adip[adata_adip.obs['d_pos']!='Non Double Posi
tives'], color=['d_pos'], size=20)
    new_cluster_names = ['Non Triple Positives','Triple Positives']
    adata_adip.rename_categories('t_pos', new_cluster_names)
    sc.pl.umap(adata_adip[adata_adip.obs['t_pos']!='Non Triple Posi
tives'], color=['t_pos'], size=20)
```

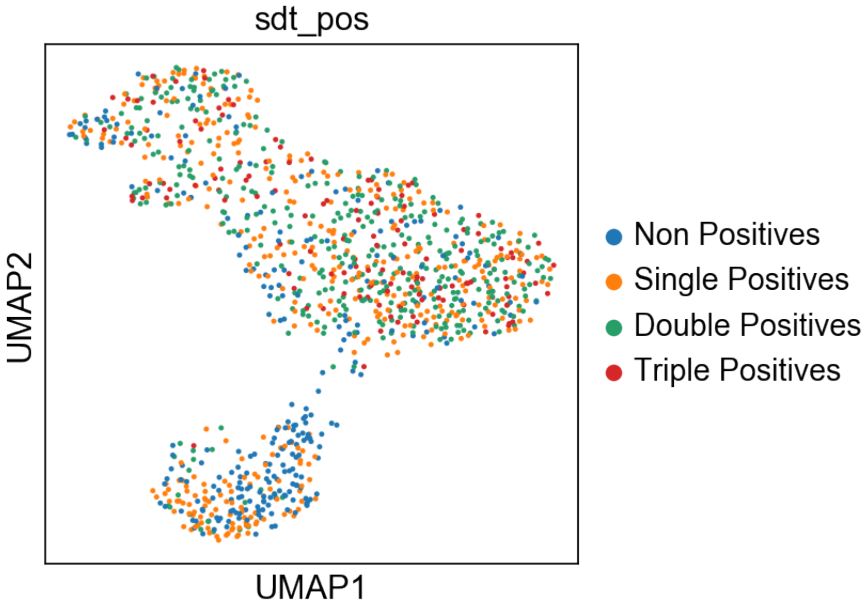


```
Trying to set attribute `.uns` of view, copying.
```

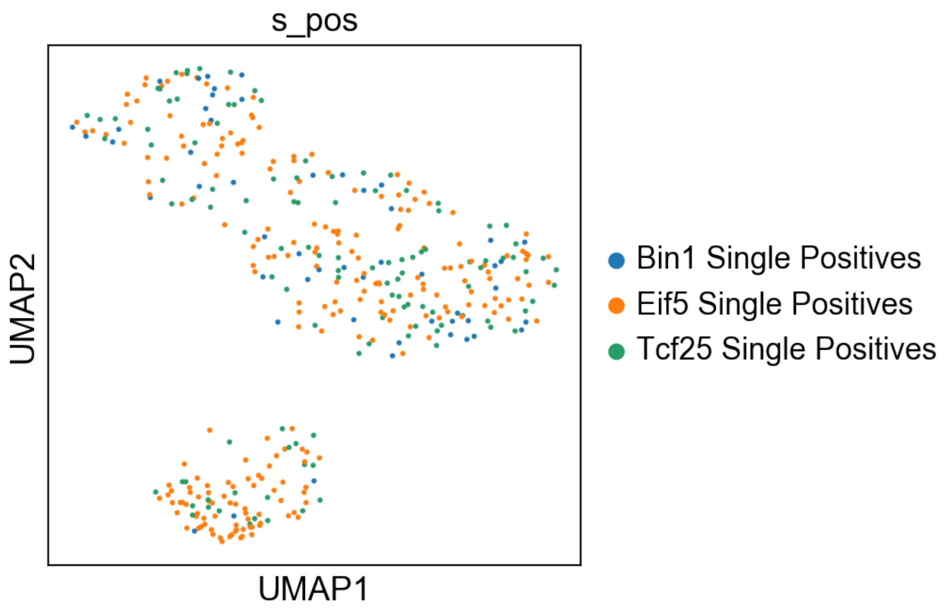


```
Trying to set attribute `.uns` of view, copying.
```

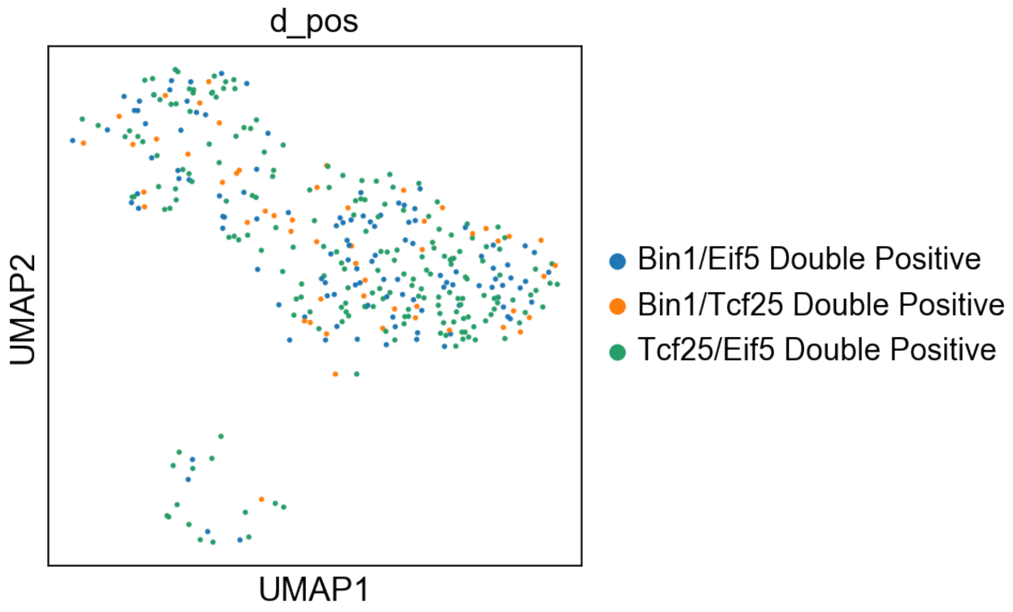


```
Trying to set attribute `.uns` of view, copying.
```

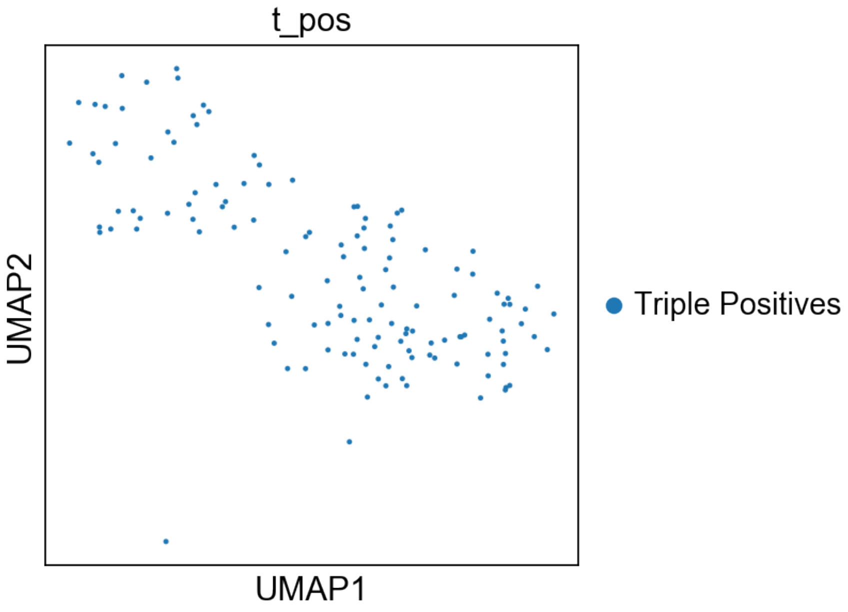

Single positives are found in all 4 clusters. Double positives are mostly in the clusters 0,1 and 3 (preadipocyte clusters) and far less present in cluster 2 (mature adipocyte cluster). Triple positives are found only in clusters 0,1 and 3 with only one exception.

Analysis separately for preadipocytes and mature adipocytes

In [58]:
```
adata_adip.obs['louvain'].head()
adata_preadip = adata_adip[adata_adip.obs['louvain']!='2']
adata_matadip = adata_adip[adata_adip.obs['louvain']=='2']
```

Preadipocyte clusters (0,1,3)

In [59]:
```
if bool_plot == True:
    sc.pl.umap(adata_preadip, color=['sdt_pos'], size=20)
    sc.pl.umap(adata_preadip[adata_preadip.obs['s_pos']!='Non Single Positives'], color=['s_pos'], size=20)
    sc.pl.umap(adata_preadip[adata_preadip.obs['d_pos']!='Non Double Positives'], color=['d_pos'], size=20)
    sc.pl.umap(adata_preadip[adata_preadip.obs['t_pos']!='Non Triple Positives'], color=['t_pos'], size=20)
```

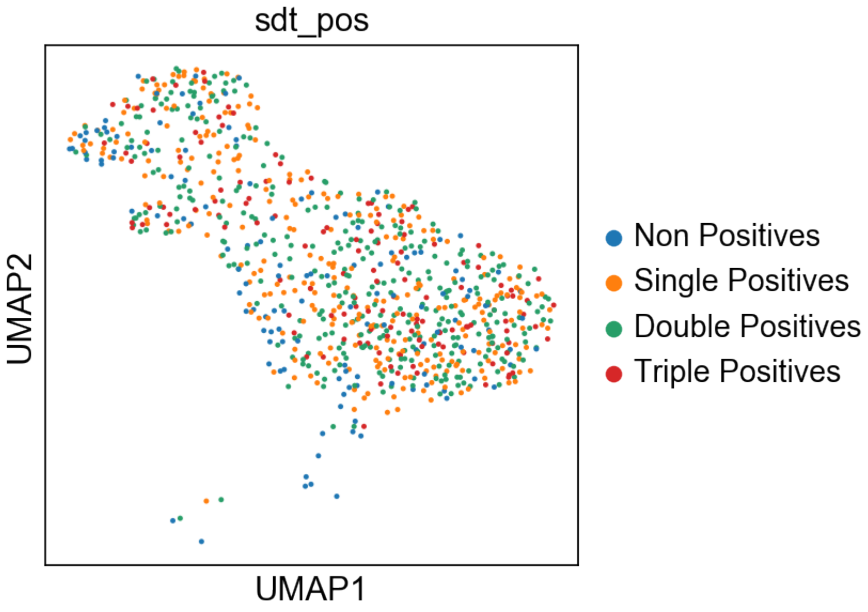


```
Trying to set attribute `.uns` of view, copying.
```

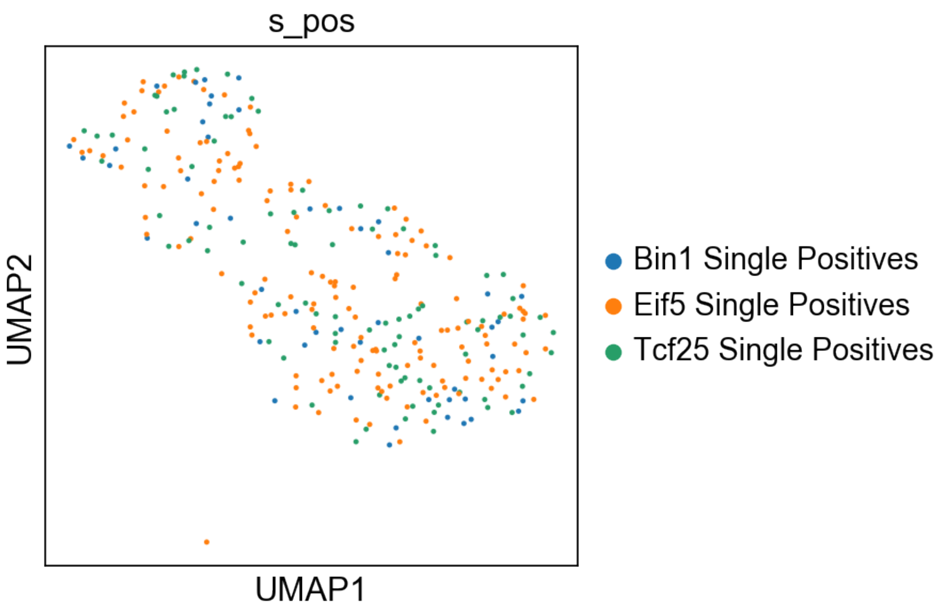


```
Trying to set attribute `.uns` of view, copying.
```

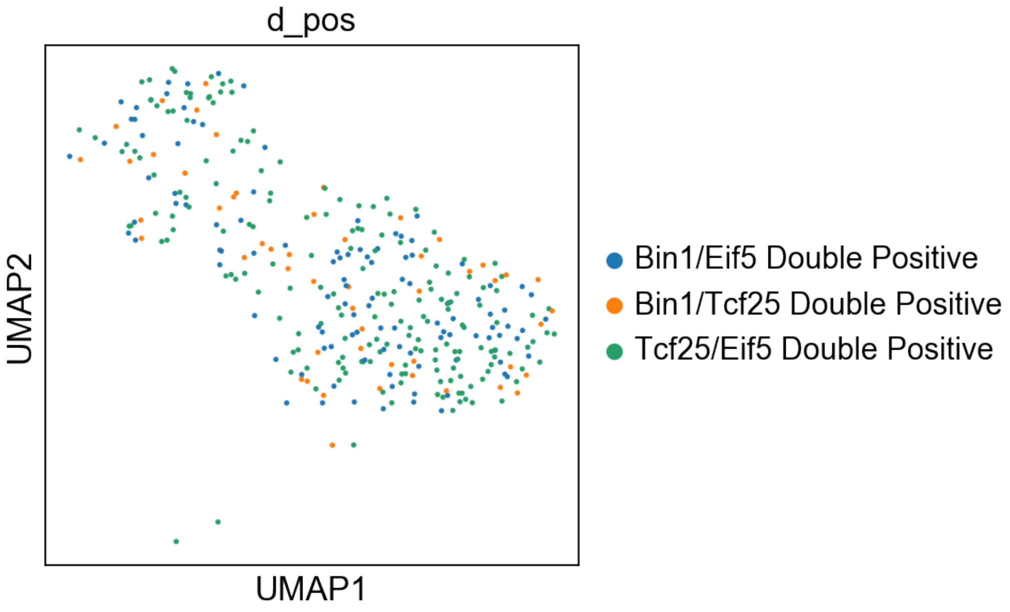


```
Trying to set attribute `.uns` of view, copying.
```

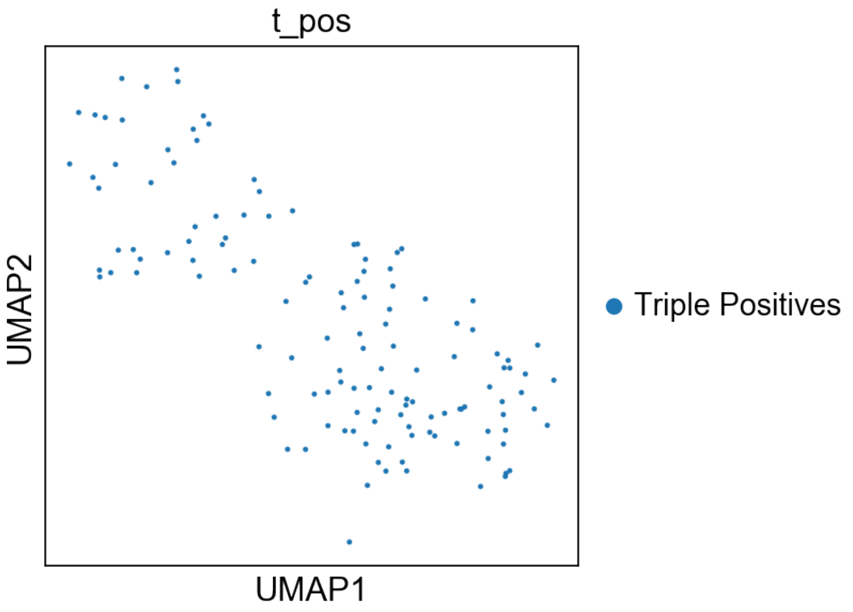

In [76]:
```
import matplotlib_venn

bin1_pos = adata_preadip.obs_names[np.asarray(adata_preadip[:,'Bin1
'].X.todense()).flatten()>0]
eif5_pos = adata_preadip.obs_names[np.asarray(adata_preadip[:,'Eif5
'].X.todense()).flatten()>0]
tcf25_pos = adata_preadip.obs_names[np.asarray(adata_preadip[:,'Tcf
25'].X.todense()).flatten()>0]

matplotlib_venn.venn3([
    set(bin1_pos),
    set(eif5_pos),
    set(tcf25_pos)
], set_labels = ("Bin1", "Eif5", "Tcf25"))
plt.savefig(dir_out + "panels/venn_target_positives_preadipocytes.p
df")
```

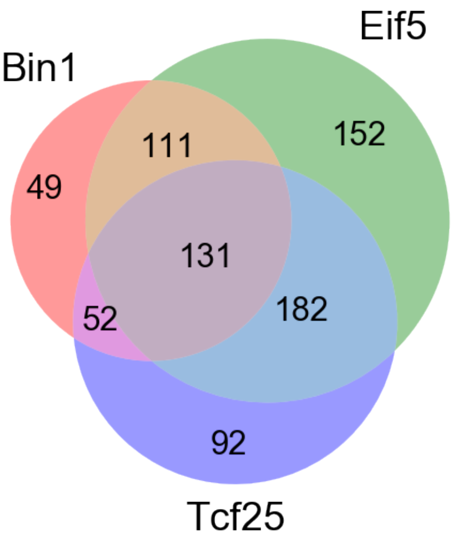

Mature adipocyte cluster

In [60]:
```
if bool_plot == True:
    sc.pl.umap(adata_matadip, color=['sdt_pos'], size=20)
    sc.pl.umap(adata_matadip[adata_matadip.obs['s_pos']!='Non Singl
e Positives'], color=['s_pos'], size=20)
    sc.pl.umap(adata_matadip[adata_matadip.obs['d_pos']!='Non Doubl
e Positives'], color=['d_pos'], size=20)
    sc.pl.umap(adata_matadip[adata_matadip.obs['t_pos']!='Non Tripl
e Positives'], color=['t_pos'], size=20)
```

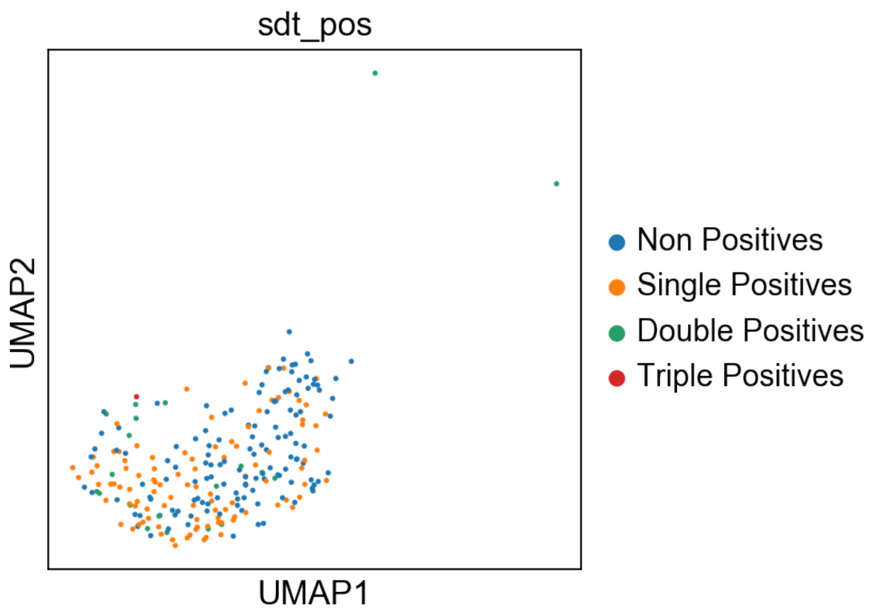


```
Trying to set attribute `.uns` of view, copying.
```

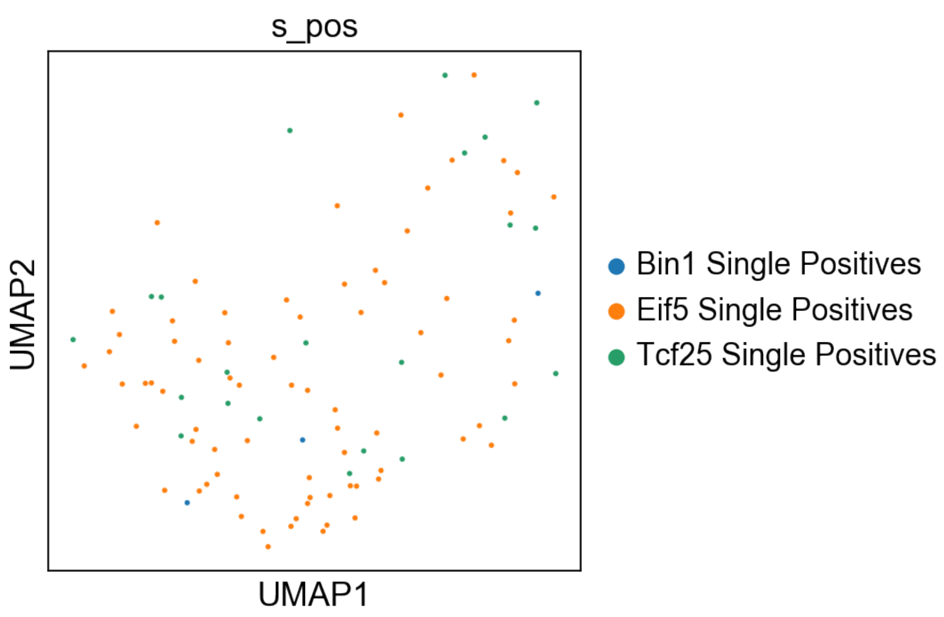


```
Trying to set attribute `.uns` of view, copying.
```

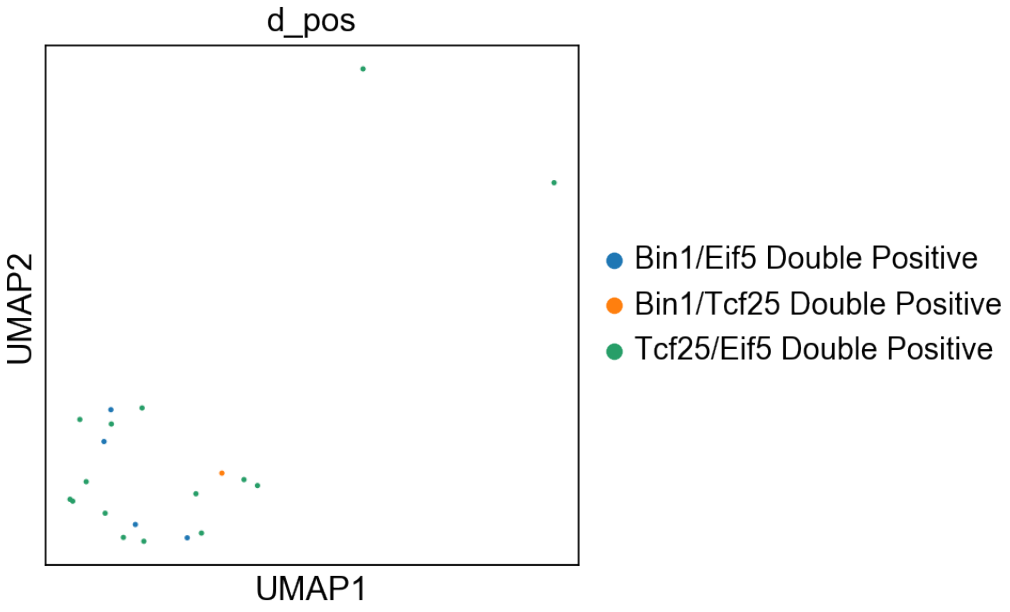


```
Trying to set attribute `.uns` of view, copying.
```

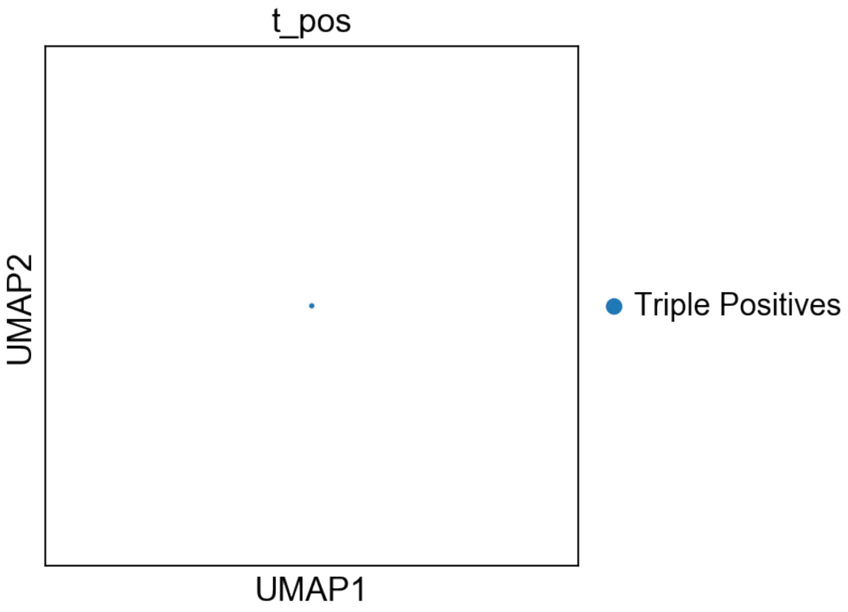

In [75]:
```
import matplotlib_venn

bin1_pos = adata_matadip.obs_names[np.asarray(adata_matadip[:,'Bin1
'].X.todense()).flatten()>0]
eif5_pos = adata_matadip.obs_names[np.asarray(adata_matadip[:,'Eif5
'].X.todense()).flatten()>0]
tcf25_pos = adata_matadip.obs_names[np.asarray(adata_matadip[:,'Tcf
25'].X.todense()).flatten()>0]

matplotlib_venn.venn3([
    set(bin1_pos),
    set(eif5_pos),
    set(tcf25_pos)
], set_labels = ("Bin1", "Eif5", "Tcf25"))
plt.savefig(dir_out + "venn_target_positives_mature_adipocytes.pdf"
)
```

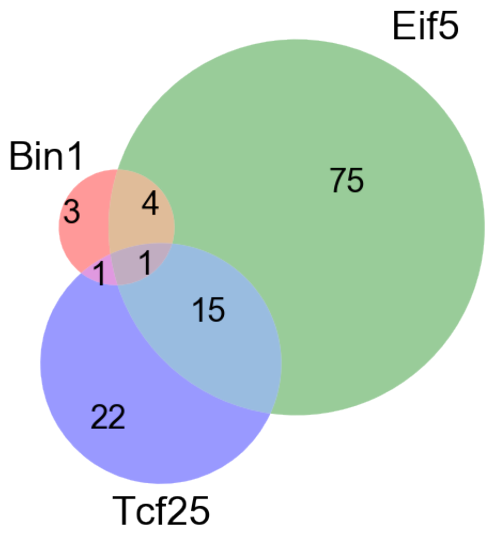

Mean Counts

Normalized

In [61]:
```
print('Bin1 mean counts: ',np.mean(adata_adip[:,'Bin1'].X))
print('Eif5 mean counts: ',np.mean(adata_adip[:,'Eif5'].X))
print('Tcf25 mean counts: ',np.mean(adata_adip[:,'Tcf25'].X))
```

```
Bin1 mean counts:  0.23998265
Eif5 mean counts:  0.6047478
Tcf25 mean counts:  0.37402338
```

Preadipocytes only

In [63]:

```
if bool_recomp == True:
    cell_ids_adip2 = np.asarray(adata_proc.obs_names)[
        [x in ['preadipocytes']
         for x in np.asarray(adata_proc.obs['celltypes'].values)]
    ]
    adata_adip2 = adata_raw[cell_ids_adip2,:].copy()
    adata_adip2.obs['n_genes'] = (adata_adip2.X > 0).sum(1)
    temp_n_counts2 = adata_adip2.obs['n_counts']
    adata_adip2.raw = adata_adip2
    adata_adip2.obs['louvain_old'] = adata_proc[cell_ids_adip2, :].
obs['louvain']
    sc.pp.normalize_per_cell(adata_adip2)
    sc.pp.log1p(adata_adip2)
    sc.pp.highly_variable_genes(adata_adip2,n_top_genes=4000)
    sc.pl.highly_variable_genes(adata_adip2)
    sc.pp.pca(adata_adip2, n_comps=50, use_highly_variable = True,
random_state=0, svd_solver='arpack')
    sc.pp.neighbors(adata_adip2, n_neighbors=100, knn=True, method=
'umap', n_pcs=50, random_state=0)
    sc.tl.umap(adata_adip2)
    if bool_recluster == True:
        sc.tl.louvain(adata_adip2, resolution=1, flavor='vtraag', r
andom_state=0)
        pd.DataFrame(adata_adip2.obs).to_csv(path_or_buf=dir_adata+
'obs_adata_adip2.csv')
    else:
        obs2 = pd.read_csv(dir_adata+'obs_adata_adip2.csv')
        adata_adip2.obs['louvain']=pd.Series(obs2['louvain'].values
, dtype = 'category')
    sc.write(dir_adata+'adata_adip2.h5ad',adata_adip2)
else:
    adata_adip2 = sc.read(dir_adata+'adata_adip2.h5ad')
sc.tl.paga(adata_adip2)
```

```
running PAGA
    finished: added
    'paga/connectivities', connectivities adjacency (adata.uns)
    'paga/connectivities_tree', connectivities subtree (adata.uns)
(0:00:00)
```

In [64]:
```
if bool_plot == True:
    sc.pl.umap(adata_adip2, color=['louvain_old', 'louvain'], size=
30, save="_preadip2_louvain_0.pdf")
    sc.pl.paga(adata_adip2, color=['louvain'], save="_preadip2_louv
ain.pdf")
```

```
WARNING: saving figure to file /Users/david.fischer/phd/data/Pread
ipocytesBrown/results/panels/umap_preadip2_louvain_0.pdf
```

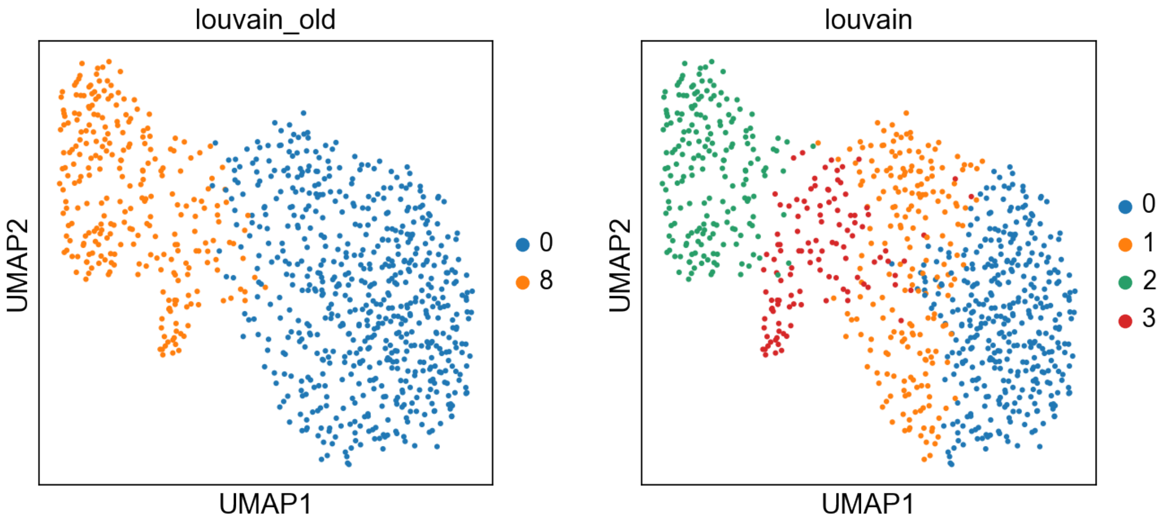


```
--> added 'pos', the PAGA positions (adata.uns['paga'])
WARNING: saving figure to file /Users/david.fischer/phd/data/Pread
ipocytesBrown/results/panels/paga_preadip2_louvain.pdf
```

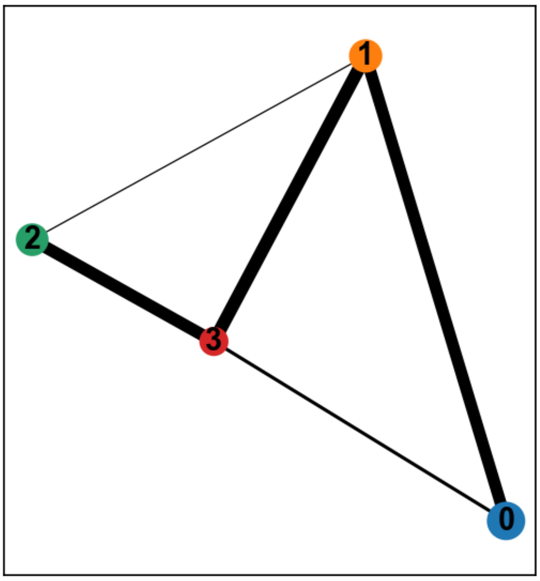

In [65]:
```
if bool_plot == True:
    plot_umap_marker(adata_adip2, adipocyte_markers.tolist(), size=
50, save="_preadip_markers_preadipcytes_norm", use_raw=False)
```

```
['Fgf10', 'Bmp4']
WARNING: saving figure to file /Users/david.fischer/phd/data/PreadipocytesBrown/results/panels/umap_preadip_markers_preadipcytes_norm_0.pdf
```

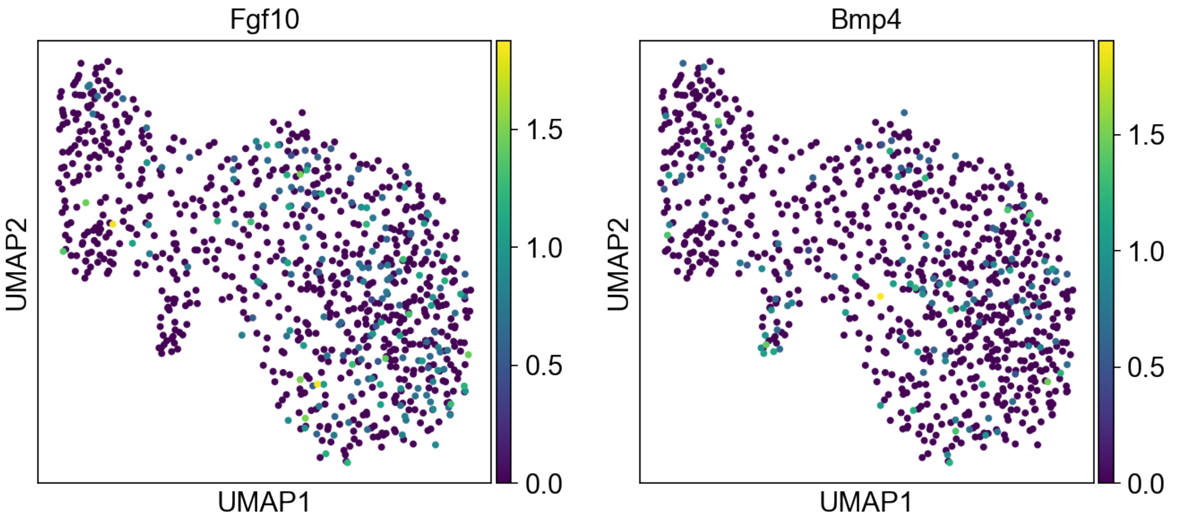


```
['Zfp423', 'Psmb8']
WARNING: saving figure to file /Users/david.fischer/phd/data/PreadipocytesBrown/results/panels/umap_preadip_markers_preadipcytes_norm_1.pdf
```

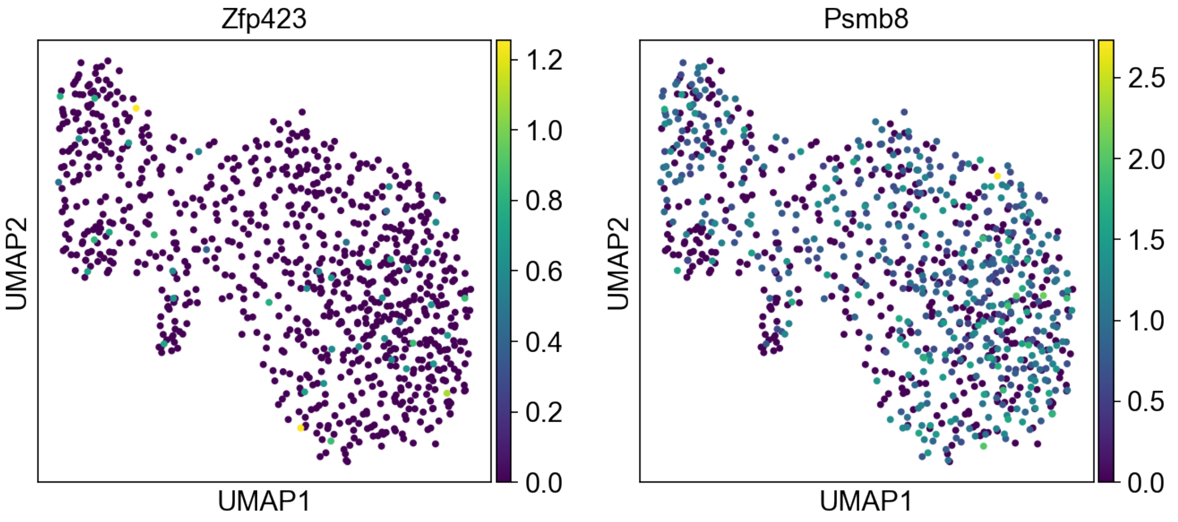


```
['Pdgfra', 'Pparg']
WARNING: saving figure to file /Users/david.fischer/phd/data/PreadipocytesBrown/results/panels/umap_preadip_markers_preadipcytes_norm_2.pdf
```

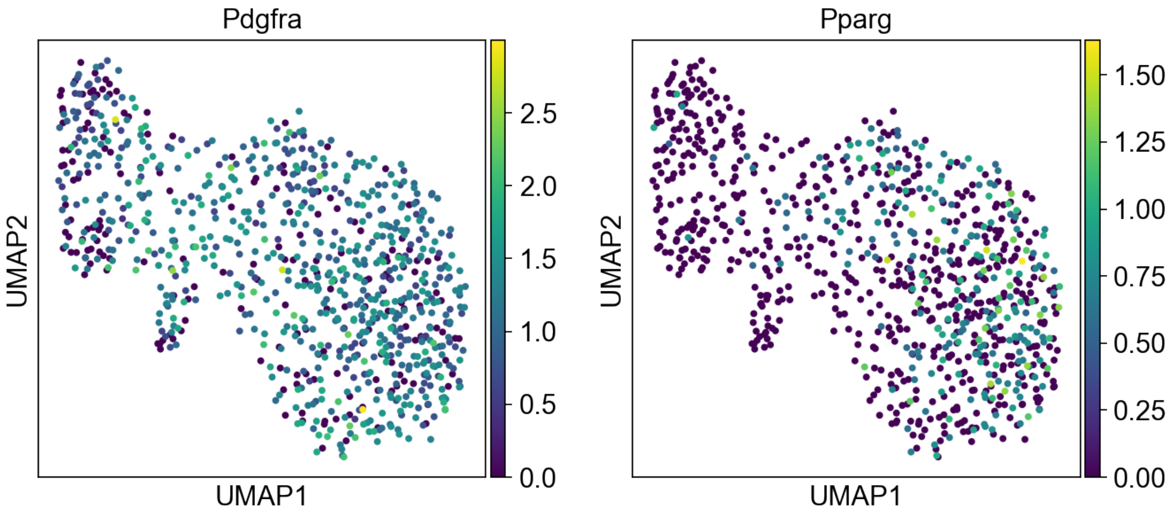


```
['Cd34', 'Dlk1']
WARNING: saving figure to file /Users/david.fischer/phd/data/PreadipocytesBrown/results/panels/umap_preadip_markers_preadipcytes_norm_3.pdf
```

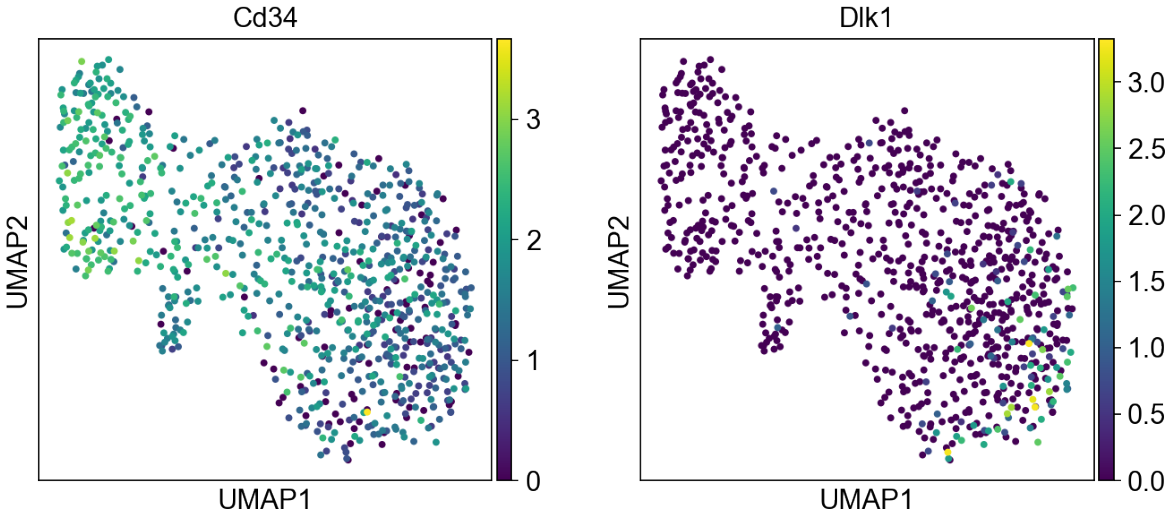


```
['Dpp4', 'Fabp4']
WARNING: saving figure to file /Users/david.fischer/phd/data/PreadipocytesBrown/results/panels/umap_preadip_markers_preadipcytes_norm_4.pdf
```

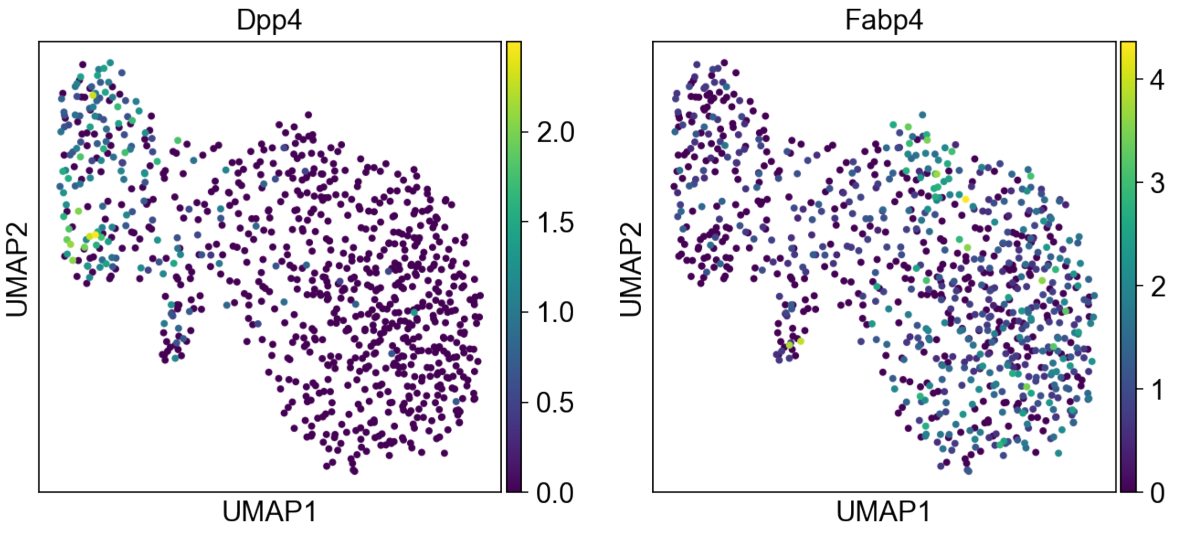

['Ucp1', 'Prdm16']
WARNING: saving figure to file /Users/david.fischer/phd/data/PreadipocytesBrown/results/panels/umap_preadip_markers_preadipcytes_norm_5.pdf

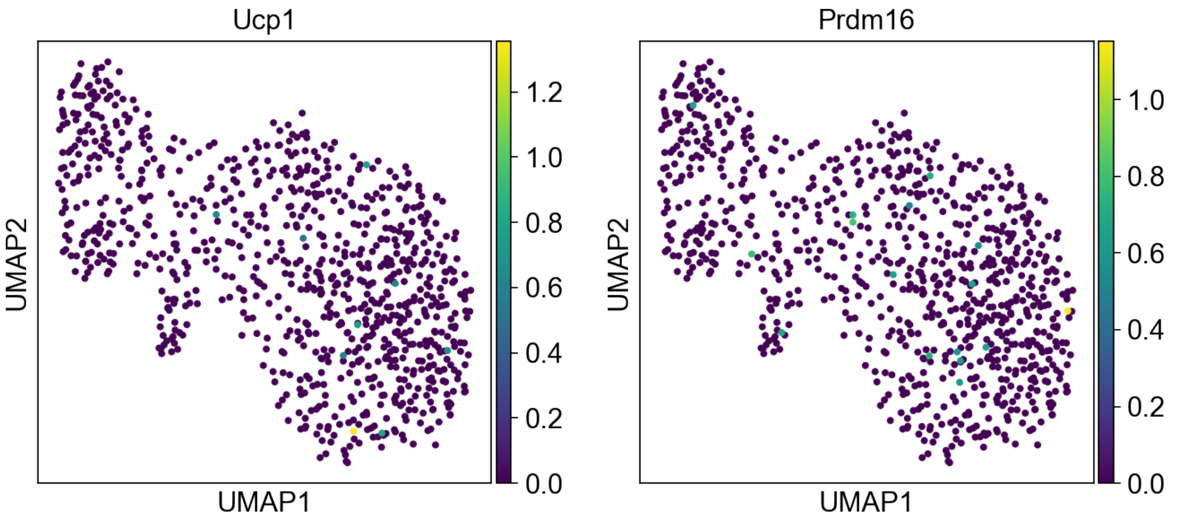

['Tcf25', 'Bin1']
WARNING: saving figure to file /Users/david.fischer/phd/data/PreadipocytesBrown/results/panels/umap_preadip_markers_preadipcytes_norm_6.pdf

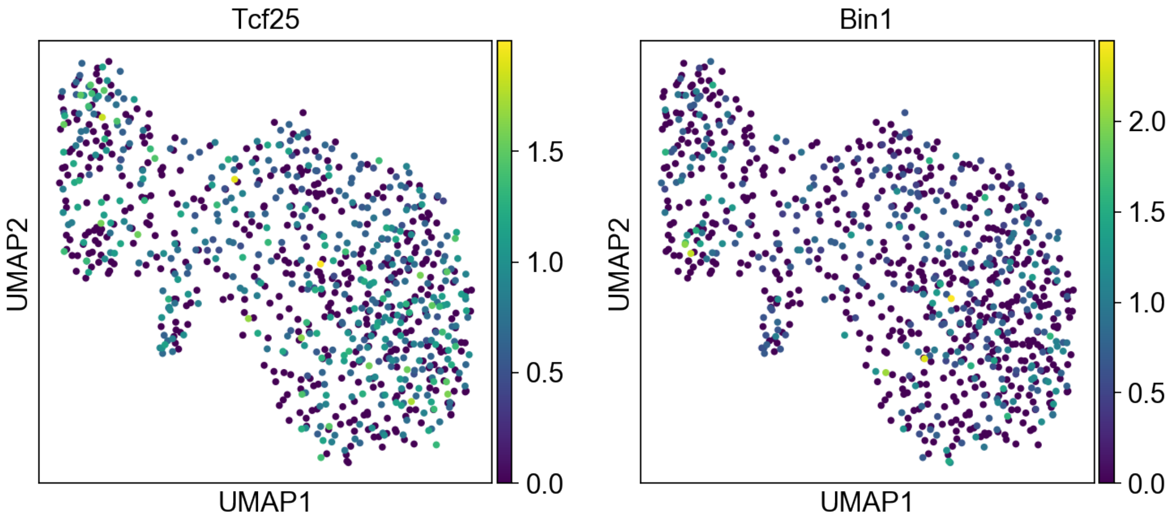


```
['Eif5', 'Slc2a4']
WARNING: saving figure to file /Users/david.fischer/phd/data/Preadipocytes Brown/results/panels/umap_preadip_markers_preadipcytes_norm_7.pdf
```

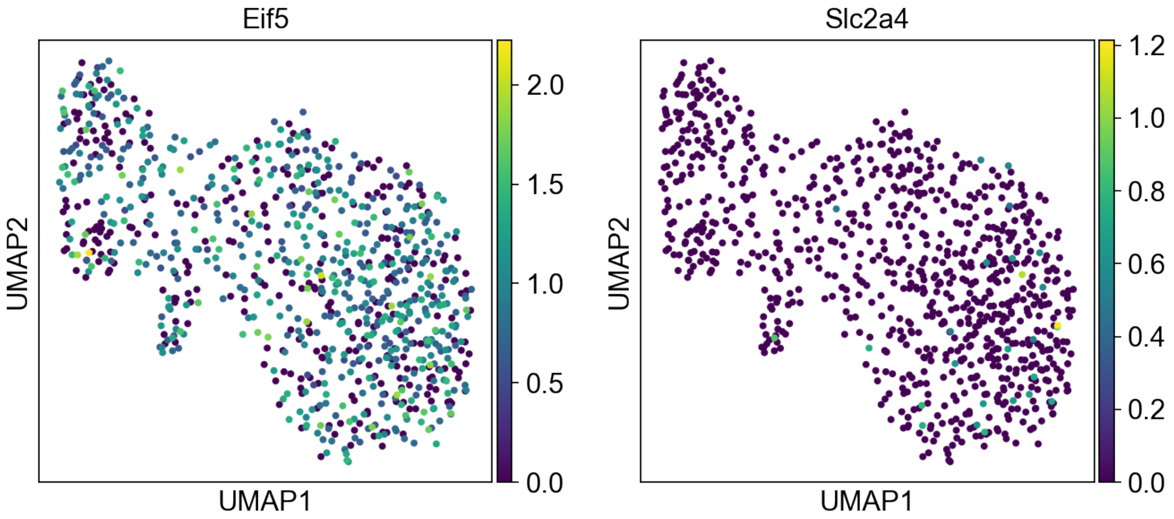


```
['Cebpa', 'Lpl']
WARNING: saving figure to file /Users/david.fischer/phd/data/PreadipocytesBrown/results/panels/umap_preadip_markers_preadipcytes_norm_8.pdf
```

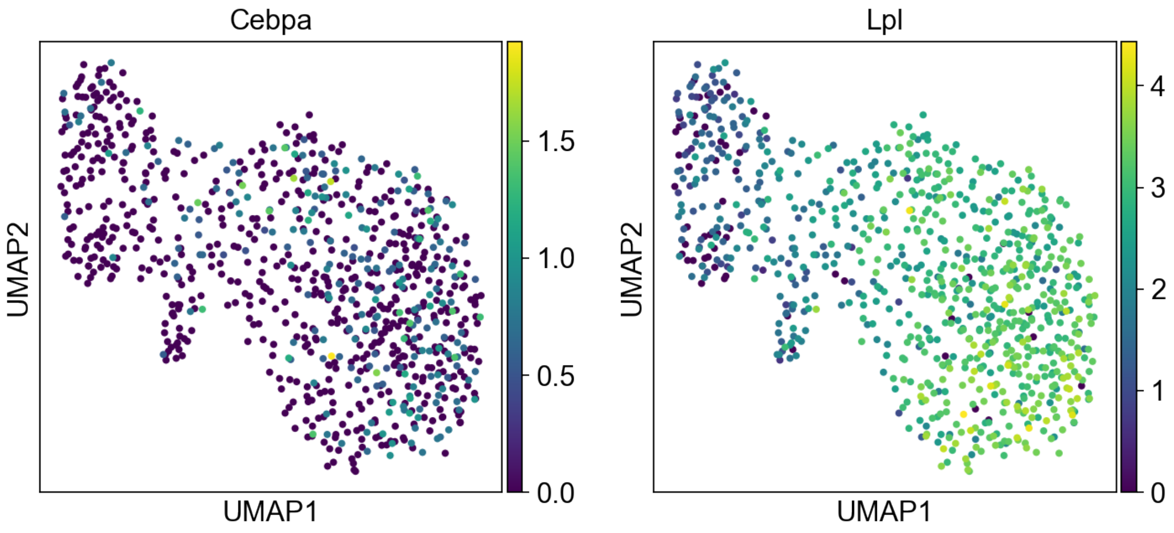


```
['Pnpla2']
WARNING: saving figure to file /Users/david.fischer/phd/data/Pread
ipocytesBrown/results/panels/umap_preadip_markers_preadipcytes_nor
m_9.pdf
```

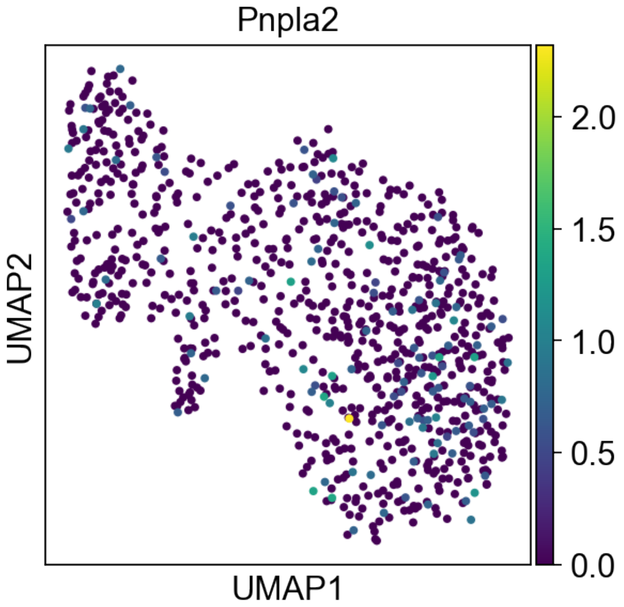

In [66]:
```
if bool_plot==True:
    sc.pl.heatmap(
        adata=adata_adip2,
        var_names=adipocyte_markers,
        groupby="louvain",
        use_raw=False,
        log=False,
        dendrogram=True,
        var_group_rotation=90,
        show_gene_labels=True,
        show=True,
        save="_preadipocyte_markers_celltypes.pdf"
    )
```

```
WARNING: dendrogram data not found (using key=dendrogram_louvain).
Running `sc.tl.dendrogram` with default parameters. For fine tunin
g it is recommended to run `sc.tl.dendrogram` independently.
    using 'X_pca' with n_pcs = 50
Storing dendrogram info using `.uns['dendrogram_louvain']`
WARNING: saving figure to file /Users/david.fischer/phd/data/Pread
ipocytesBrown/results/panels/heatmap_preadipocyte_markers_celltype
s.pdf
```

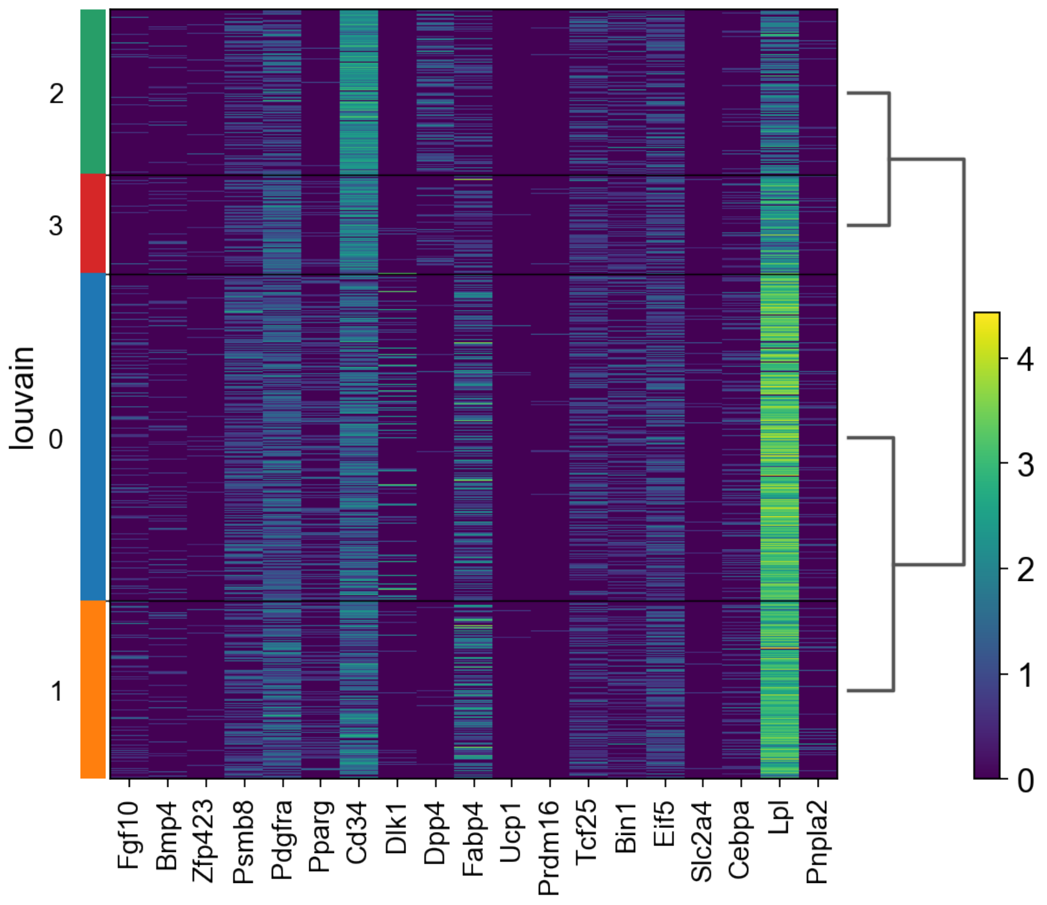

In [67]:
```
sc.tl.rank_genes_groups(adata_adip2, groupby='louvain', key_added='
rank_genes')
if bool_plot == True:
    sc.pl.rank_genes_groups(adata_adip, key='rank_genes', groups=['
0','1','2','3'], fontsize=12, save="_preadip_genes")
```

```
ranking genes
    finished: added to `.uns['rank_genes']`
    'names', sorted np.recarray to be indexed by group ids
    'scores', sorted np.recarray to be indexed by group ids
    'logfoldchanges', sorted np.recarray to be indexed by group id
s
    'pvals', sorted np.recarray to be indexed by group ids
    'pvals_adj', sorted np.recarray to be indexed by group ids (0:
00:00)
WARNING: saving figure to file /Users/david.fischer/phd/data/Pread
ipocytesBrown/results/panels/rank_genes_groups_louvain_preadip_gen
es.pdf
```

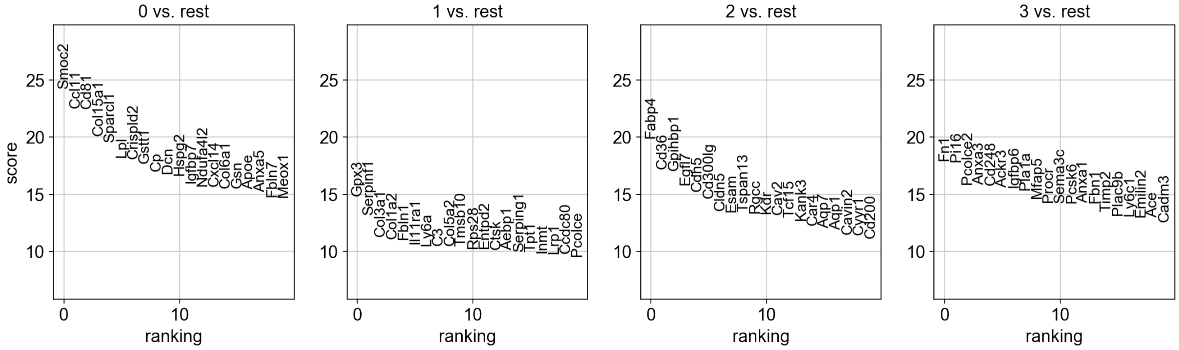

Supplement: Supplementary file 4 [file LSA-2020-00924_Supplemental_Data_1.pdf]
