# Supplementary material for: Identification and characterization of distinct brown adipocyte subtypes in C57BL/6J mice
Source: Life Sci Alliance. 2020 Nov 30;4(1):e202000924. doi: 10.26508/lsa.202000924 (PMC7723269; doi:10.26508/lsa.202000924)
Supplement: Supplementary file 5 [file LSA-2020-00924_TableS1.docx]

**Supplementary Table 1. qPCR primers used in qRT-PCR.**

| **Genes of interest** | **Sequence (5’ to 3’)** |
| --- | --- |
| **Mouse** | |
| Tbp fwd/rev | ACCCTTCACCAATGACTCCTATG / TGACTGCAGCAAATCGCTTGG |
| B2m fwd/rev | CCCCACTGAGACTGATACATACGC / AGAAACTGGATTTGTAATTAAGCAGGTTC |
| Ucp1 fwd/rev | CTGCCAGGACAGTACCCAAG / TCAGCTGTTCAAAGCACACA |
| Pparγ fwd/rev | CCCTGGCAAAGCATTTGTAT / GAAACTGGCACCCTTGAAAA |
| Ppargc1a fwd/rev | AGCCGTGACCACTGACAACGAG / GCTGCATGGTTCTGAGTGCTAAG |
| Prdm16 fwd/rev | CCGCTGTGATGAGTGTGATG / GGACGATCATGTGTTGCTCC |
| P2rx5 fwd/rev | CACTCTGCAGGGAAGTGTCA / CTGCAGCTCACCATCCTGT |
| Pat2 fwd/rev | ATCAGCTGCCACAAGAACG / GGCAATGATGACCAGGCT |
| Tmem26 fwd/rev | GAAACCAGTATTGCAGCACCC / CCCATTCCATTGGTGGCTCT |
| Cd137 fwd/rev | GCCGAACTGTAACATCTGCA / TTCAATGCACTCACACTCCG |
| Asc1 fwd/rev | GCCTGGCTGGATTCCTACTG / GGAAAGACAGGCTGAAGCAC |
| Fkbp8 fwd/rev | GGCTATCACCTCCAACACCA / TCAGACACTTGACCTTCAGCT |
| Mtmr9 fwd/rev | AGGCTCATTATCCCCAGTGG / ACATGCTTCCACGAGTTTGA |
| Kars fwd/rev | CTAAACCAGACTGCTTCCGC / CGGCTTGACTTCGGATCTTG |
| Bin1 fwd/rev | GGCTACCATCCCCAAGTCC / CCTTGGATGGGGTGTGTTTG |
| Eif5 fwd/rev | GTACCGAGGCATGCTTGAC / TCTTTCCTGTACCAATGTCACT |
| Tcf25 fwd/rev | AGGAGTCCGTGTCAACAACC / GGCAGGGTACAGTCAGATCT |
| Myf5 fwd/rev | GAACTATTACAGCCTGCCGG / AAAGCTGCTGTTCTTTCGGG |
| **Human** | |
| BIN1 fwd/rev | GCAGAGATGGGCAGTAAAGG / TTGGTCTCATCTGCCTTCCC |
| EIF5 fwd/rev | TATCGCTACAAGATGCCCCG / CCTTTGCAACGTCAACCATG |
| TCF25 fwd/rev | AAAGGTATTTTGGTGCCCGG / GTGCTTTTAGGGGTGGTCAG |
| UCP1 fwd/rev | ACCGCAGGGAAAGAAACAGC / TCAGATTGGGAGTAGTCCCT |
