# Supplementary material for: Identification and characterization of distinct brown adipocyte subtypes in C57BL/6J mice
Source: Life Sci Alliance. 2020 Nov 30;4(1):e202000924. doi: 10.26508/lsa.202000924 (PMC7723269; doi:10.26508/lsa.202000924)
Supplement: Supplementary file 6 [file LSA-2020-00924_Supplemental_Data_2.pdf]

Assay Class:	Eukaryote Total RNA Nano	Created:	31.05.2016 15:33:48
Data Path:	C:\...Eukaryote Total RNA Nano_DE13806198_2016-05-31_15-33-49.xad	Modified:	31.05.2016 15:57:45

Electrophoresis File Run Summary

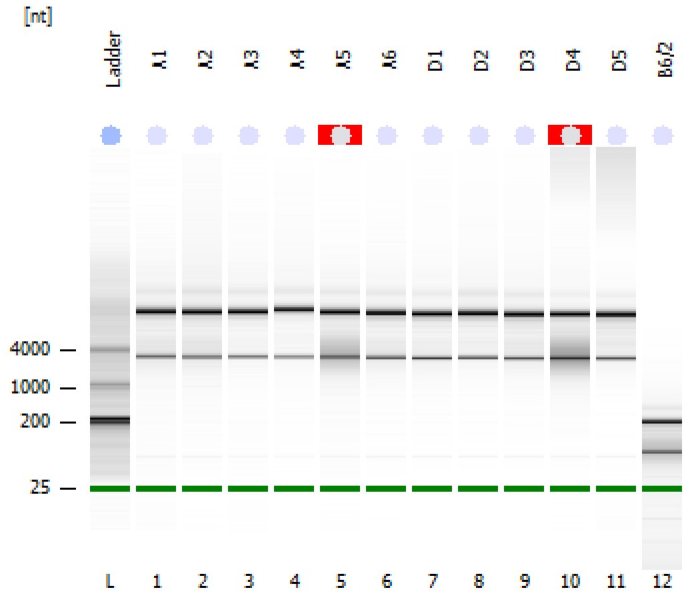

Instrument Information:

Instrument Name:	DE13806198	Firmware:	C.01.069
Serial#:	DE13806198	Type:	G2939A

Assay Information:

Assay Origin Path:	C:\Program Files\Agilent\2100 bioanalyzer\2100 expert\assays\RNA\Eukaryote Total RNA Nano Series II.xsy
Assay Class:	Eukaryote Total RNA Nano
Version:	2.6
Assay Comments:	Total RNA Analysis ng sensitivity (Eukaryote)

© Copyright 2003 - 2009 Agilent Technologies, Inc.

Chip Information:

Chip Lot #:

Reagent Kit Lot #:

Chip Comments:

A1

RIN: 9.40

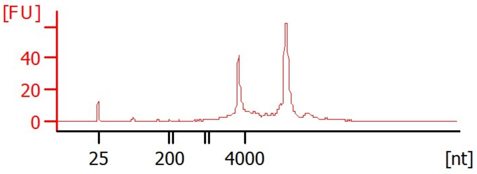

A2

RIN: 9.50

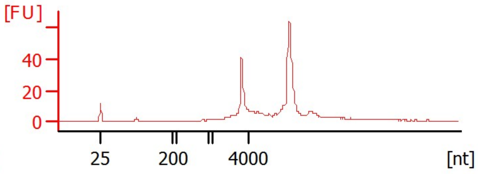

A3

RIN:10

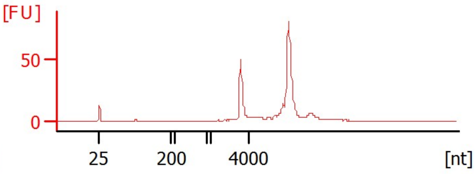

A4

RIN: 9.90

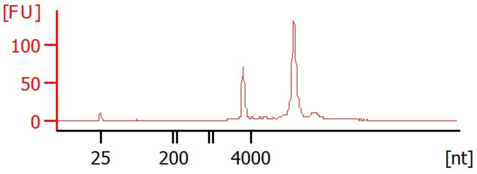

A5

RIN N/A

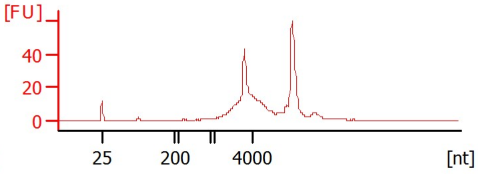

A6

RIN: 9.50

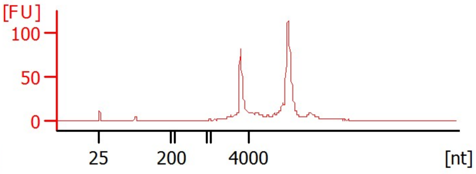

D1

RIN: 9.90

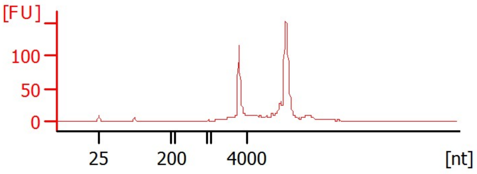

D2

RIN: 9.80

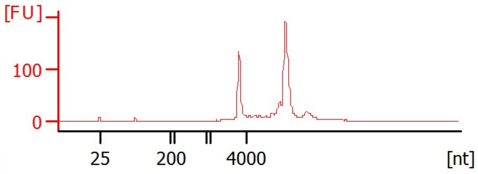

D3

RIN: 9.70

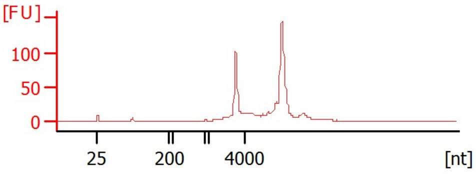

D4

RIN N/A

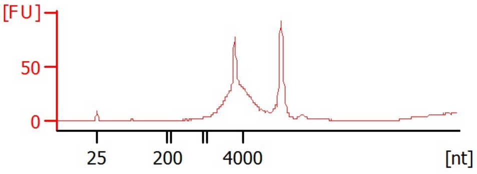

D5

RIN:10

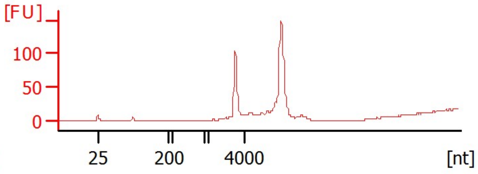

B6/2

RIN:2

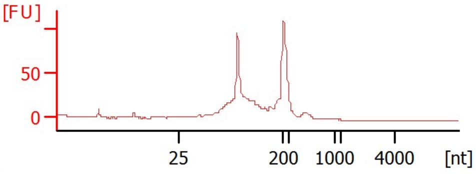

Assay Class: Eukaryote Total RNA Nano
Data Path: C:\...Eukaryote Total RNA Nano_DE13806198_2016-05-31_15-33-49.xad

Created: 31.05.2016 15:33:48
Modified: 31.05.2016 15:57:45

Electrophoresis File Run Summary (Chip Summary)

Sample Name	Sample Comment	Status	Result Label	Result Color
A1		✔	RIN: 9.40	
A2		✔	RIN: 9.50	
A3		✔	RIN:10	
A4		✔	RIN: 9.90	
A5		✔	RIN N/A	
A6		✔	RIN: 9.50	
D1		✔	RIN: 9.90	
D2		✔	RIN: 9.80	
D3		✔	RIN: 9.70	
D4		✔	RIN N/A	
D5		✔	RIN:10	
B6/2		✔	RIN:2	
Ladder		✔	All Other Samples	

Chip Lot # **Reagent Kit Lot #**

Chip Comments :

© Copyright 2003 - 2009 Agilent Technologies, Inc.

Assay Class:	Eukaryote Total RNA Nano	Created:	31.05.2016 15:33:48
Data Path:	C:\...Eukaryote Total RNA Nano_DE13806198_2016-05-31_15-33-49.xad	Modified:	31.05.2016 15:57:45

Electrophoresis Assay Details

General Analysis Settings

Number of Available Sample and Ladder Wells (Max.) : 13
Minimum Visible Range [s] : 17
Maximum Visible Range [s] : 70
Start Analysis Time Range [s] : 19
End Analysis Time Range [s] : 69
Ladder Concentration [ng/µl] : 150
Lower Marker Concentration [ng/µl] : 0
Upper Marker Concentration [ng/µl] : 0
Used Lower Marker for Quantitation
Standard Curve Fit is Logarithmic
Show Data Aligned to Lower Marker

Integrator Settings

Integration Start Time [s] : 19
Integration End Time [s] : 69
Slope Threshold : 0,6
Height Threshold [FU] : 0,5
Area Threshold : 0,2
Width Threshold [s] : 0,5
Baseline Plateau [s] : 6

Filter Settings

Filter Width [s] : 0,5
Polynomial Order : 4

Ladder

Ladder Peak	Size
1	25
2	200
3	500
4	1000
5	2000
6	4000

 © Copyright 2003 - 2009 Agilent Technologies, Inc.

Assay Class: Eukaryote Total RNA Nano Created: 31.05.2016 15:33:48
Data Path: C:\...Eukaryote Total RNA Nano_DE13806198_2016-05-31_15-33-49.xad Modified: 31.05.2016 15:57:45

Electropherogram Summary

Ladder

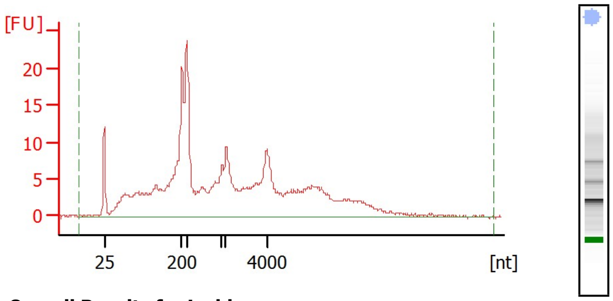

Overall Results for Ladder

RNA Area:	374,5
RNA Concentration:	150 ng/µl
Result Flagging Color:	
Result Flagging Label:	All Other Samples

A1

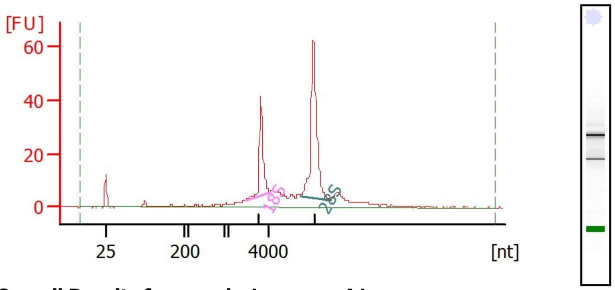

Overall Results for sample 1 : A1

RNA Area:	308,1
RNA Concentration:	123 ng/µl
rRNA Ratio [28s / 18s]:	2,2
RNA Integrity Number (RIN):	9.4 (B.02.08)
Result Flagging Color:	
Result Flagging Label:	RIN: 9.40

Fragment table for sample 1 : A1

Name	Start Size [nt]	End Size [nt]	Area	% of total Area
18S	2.871	4.064	45,1	14,6
28S	5.545	7.068	101,0	32,8

A2

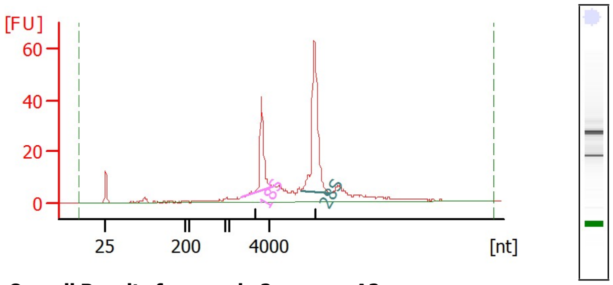

Overall Results for sample 2 : A2

RNA Area:	317,0
RNA Concentration:	127 ng/µl
rRNA Ratio [28s / 18s]:	2,3
RNA Integrity Number (RIN):	9.5 (B.02.08)
Result Flagging Color:	
Result Flagging Label:	RIN: 9.50

Fragment table for sample 2 : A2

Name	Start Size [nt]	End Size [nt]	Area	% of total Area
18S	2.641	4.072	43,4	13,7
28S	5.503	6.933	100,0	31,6

A3

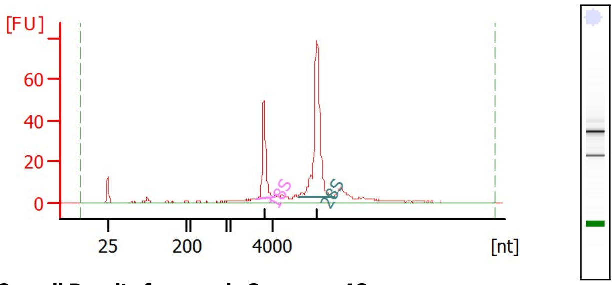

Overall Results for sample 3 : A3

RNA Area:	322,8
RNA Concentration:	129 ng/µl
rRNA Ratio [28s / 18s]:	2,6
RNA Integrity Number (RIN):	10 (B.02.08)
Result Flagging Color:	
Result Flagging Label:	RIN:10

Fragment table for sample 3 : A3

Name	Start Size [nt]	End Size [nt]	Area	% of total Area
18S	3.259	4.095	51,3	15,9
28S	5.257	7.011	131,8	40,8

 © Copyright 2003 - 2009 Agilent Technologies, Inc.

Assay Class: Eukaryote Total RNA Nano
Data Path: C:\...Eukaryote Total RNA Nano_DE13806198_2016-05-31_15-33-49.xad

Created: 31.05.2016 15:33:48
Modified: 31.05.2016 15:57:45

Electropherogram Summary Continued ...

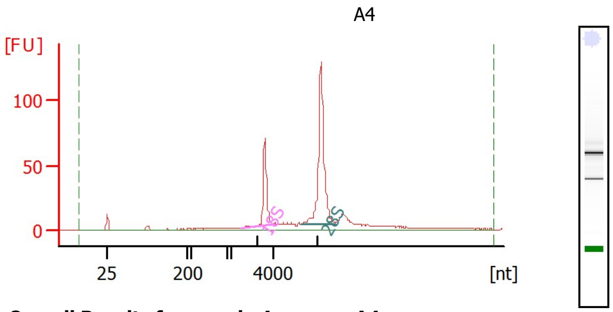

Overall Results for sample 4 : A4

RNA Area:	485,7
RNA Concentration:	195 ng/µl
rRNA Ratio [28s / 18s]:	2,7
RNA Integrity Number (RIN):	9.9 (B.02.08)
Result Flagging Color:	
Result Flagging Label:	RIN: 9.90

Fragment table for sample 4 : A4

Name	Start Size [nt]	End Size [nt]	Area	% of total Area
18S	2.442	3.961	74,1	15,3
28S	5.399	6.980	197,4	40,6

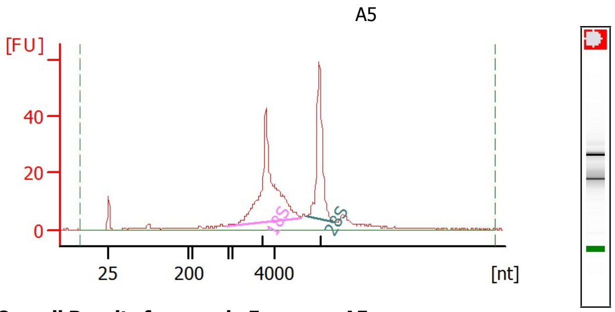

Overall Results for sample 5 : A5

RNA Area:	393,3
RNA Concentration:	158 ng/µl
rRNA Ratio [28s / 18s]:	0,6
RNA Integrity Number (RIN):	N/A (B.02.08)
Result Flagging Color:	
Result Flagging Label:	RIN N/A

Fragment table for sample 5 : A5

Name	Start Size [nt]	End Size [nt]	Area	% of total Area
18S	970	5.258	154,0	39,2
28S	5.521	6.878	91,6	23,3

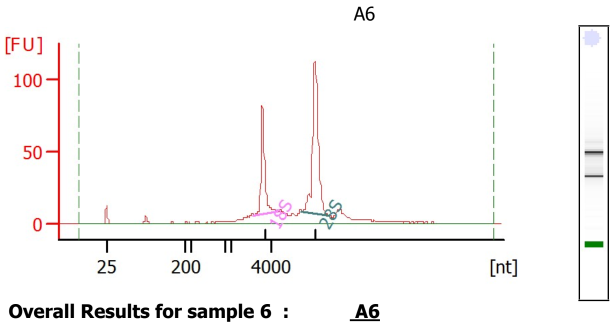

Overall Results for sample 6 : A6

RNA Area:	542,6
RNA Concentration:	217 ng/µl
rRNA Ratio [28s / 18s]:	2,0
RNA Integrity Number (RIN):	9.5 (B.02.08)
Result Flagging Color:	
Result Flagging Label:	RIN: 9.50

Fragment table for sample 6 : A6

Name	Start Size [nt]	End Size [nt]	Area	% of total Area
18S	3.141	4.281	89,1	16,4
28S	5.481	6.784	181,8	33,5

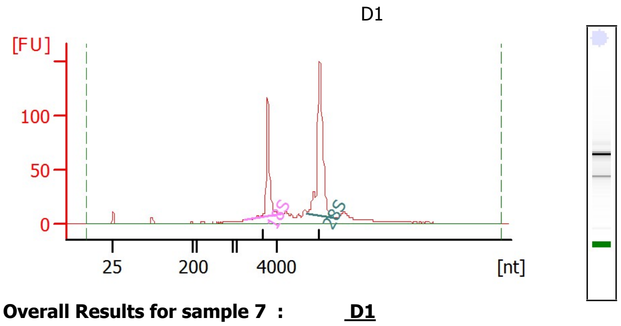

Overall Results for sample 7 : D1

RNA Area:	636,3
RNA Concentration:	255 ng/µl
rRNA Ratio [28s / 18s]:	1,9
RNA Integrity Number (RIN):	9.9 (B.02.08)
Result Flagging Color:	
Result Flagging Label:	RIN: 9.90

Fragment table for sample 7 : D1

Name	Start Size [nt]	End Size [nt]	Area	% of total Area
18S	2.437	4.256	122,8	19,3
28S	5.441	6.729	231,2	36,3

© Copyright 2003 - 2009 Agilent Technologies, Inc.

Assay Class:	Eukaryote Total RNA Nano	Created:	31.05.2016 15:33:48
Data Path:	C:\...Eukaryote Total RNA Nano_DE13806198_2016-05-31_15-33-49.xad	Modified:	31.05.2016 15:57:45

Electropherogram Summary Continued ...

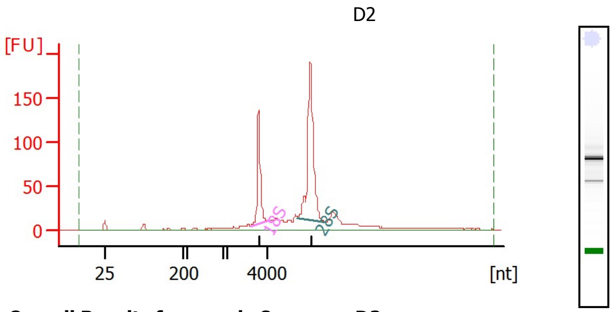

Overall Results for sample 8 : D2

RNA Area:	833,9
RNA Concentration:	334 ng/µl
rRNA Ratio [28s / 18s]:	2,2
RNA Integrity Number (RIN):	9.8 (B.02.08)
Result Flagging Color:	
Result Flagging Label:	RIN: 9.80

Fragment table for sample 8 : D2

Name	Start Size [nt]	End Size [nt]	Area	% of total Area
18S	3.159	4.043	138,7	16,6
28S	5.463	6.800	304,5	36,5

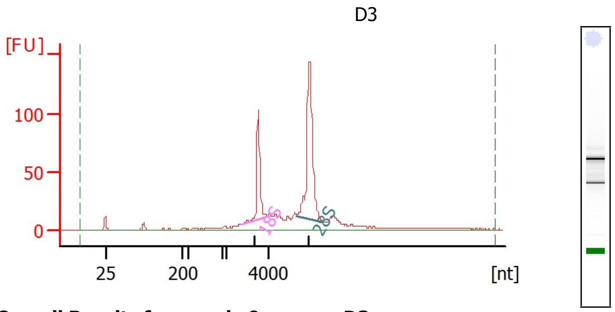

Overall Results for sample 9 : D3

RNA Area:	664,9
RNA Concentration:	266 ng/µl
rRNA Ratio [28s / 18s]:	2,2
RNA Integrity Number (RIN):	9.7 (B.02.08)
Result Flagging Color:	
Result Flagging Label:	RIN: 9.70

Fragment table for sample 9 : D3

Name	Start Size [nt]	End Size [nt]	Area	% of total Area
18S	2.701	3.979	101,9	15,3
28S	5.402	6.701	226,5	34,1

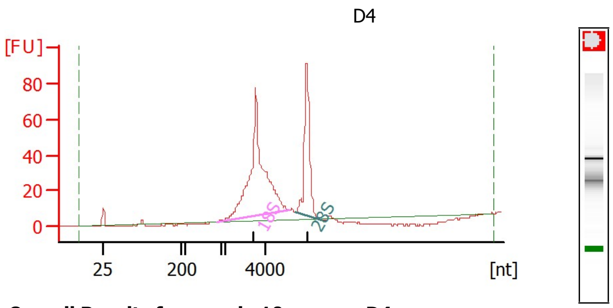

Overall Results for sample 10 : D4

RNA Area:	511,3
RNA Concentration:	205 ng/µl
rRNA Ratio [28s / 18s]:	0,4
RNA Integrity Number (RIN):	N/A (B.02.08)
Result Flagging Color:	
Result Flagging Label:	RIN N/A

Fragment table for sample 10 : D4

Name	Start Size [nt]	End Size [nt]	Area	% of total Area
18S	947	5.216	301,4	59,0
28S	5.506	6.769	132,0	25,8

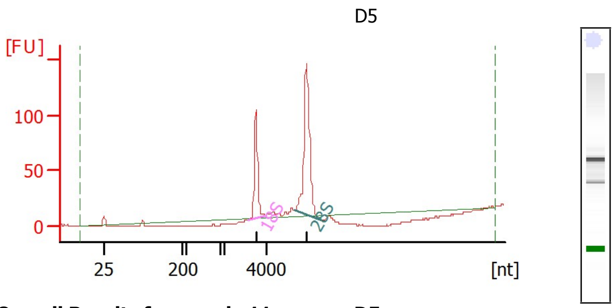

Overall Results for sample 11 : D5

RNA Area:	377,9
RNA Concentration:	151 ng/µl
rRNA Ratio [28s / 18s]:	2,2
RNA Integrity Number (RIN):	10 (B.02.08)
Result Flagging Color:	
Result Flagging Label:	RIN:10

Fragment table for sample 11 : D5

Name	Start Size [nt]	End Size [nt]	Area	% of total Area
18S	3.098	3.970	108,5	28,7
28S	5.382	6.731	238,6	63,2

 © Copyright 2003 - 2009 Agilent Technologies, Inc.

Assay Class:	Eukaryote Total RNA Nano	Created:	31.05.2016 15:33:48
Data Path:	C:\...Eukaryote Total RNA Nano_DE13806198_2016-05-31_15-33-49.xad	Modified:	31.05.2016 15:57:45

Electropherogram Summary Continued ...

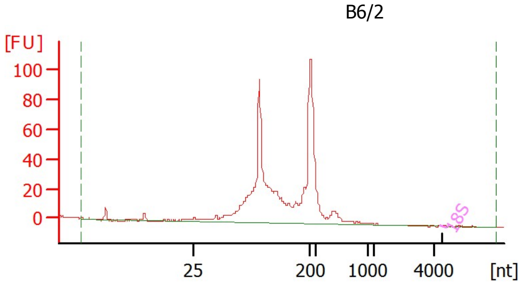

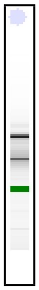

Overall Results for sample 12 : **B6/2**

RNA Area:	698,2
RNA Concentration:	280 ng/µl
rRNA Ratio [28s / 18s]:	0,0
RNA Integrity Number (RIN):	2 (B.02.08)
Result Flagging Color:	
Result Flagging Label:	RIN:2

Fragment table for sample 12 : **B6/2**

Name	Start Size [nt]	End Size [nt]	Area	% of total Area
18S	4.225	4.450	0,2	0,0

 © Copyright 2003 - 2009 Agilent Technologies, Inc.

Assay Class: Eukaryote Total RNA Nano
Data Path: C:\...Eukaryote Total RNA Nano_DE13806198_2016-05-31_15-33-49.xad
Created: 31.05.2016 15:33:48
Modified: 31.05.2016 15:57:45

Gel Image

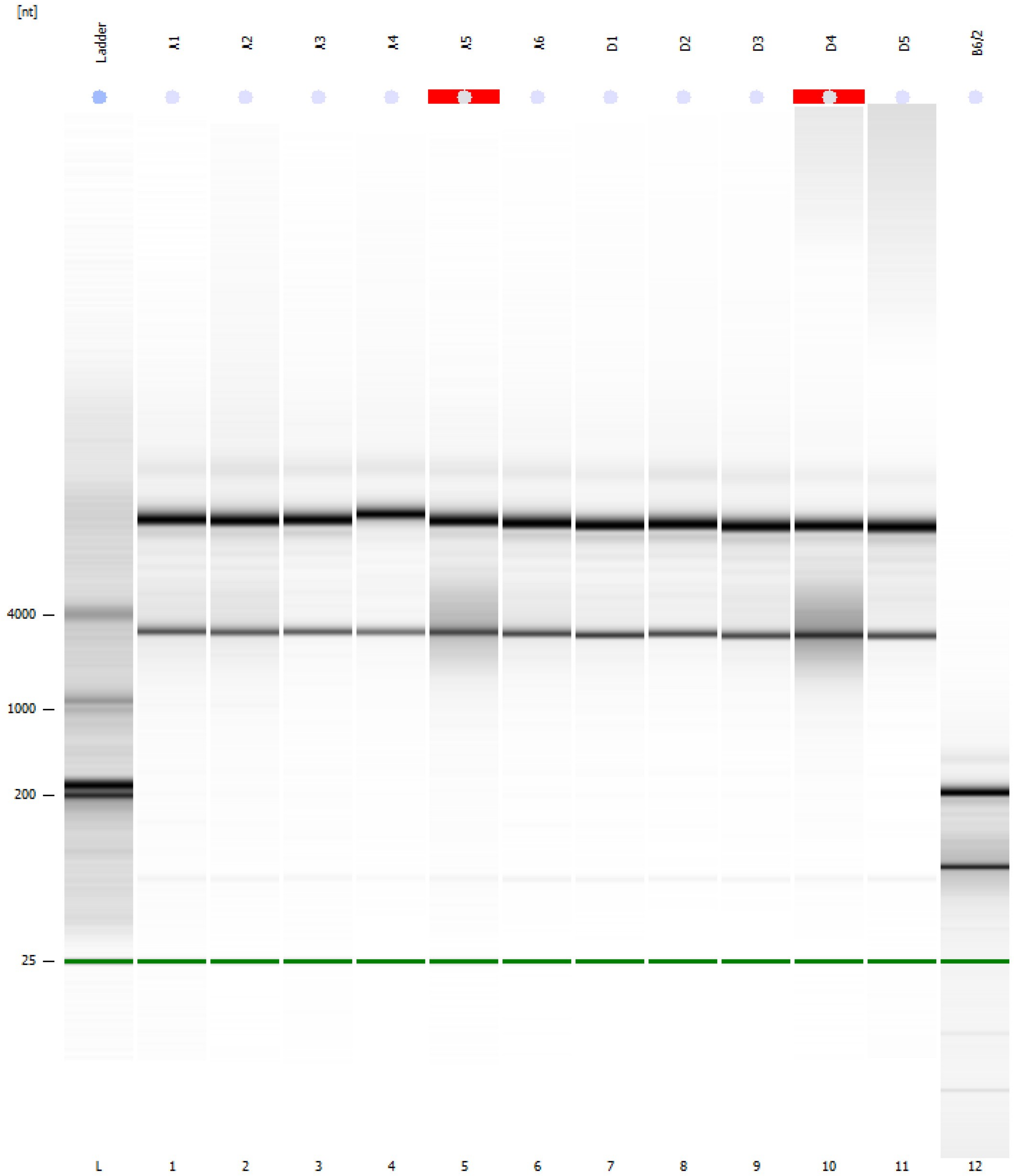

 © Copyright 2003 - 2009 Agilent Technologies, Inc.

Assay Class: Eukaryote Total RNA Nano
Data Path: C:\...Eukaryote Total RNA Nano_DE13806198_2016-05-31_15-33-49.xad
Created: 31.05.2016 15:33:48
Modified: 31.05.2016 15:57:45

Curves

Standard Curve

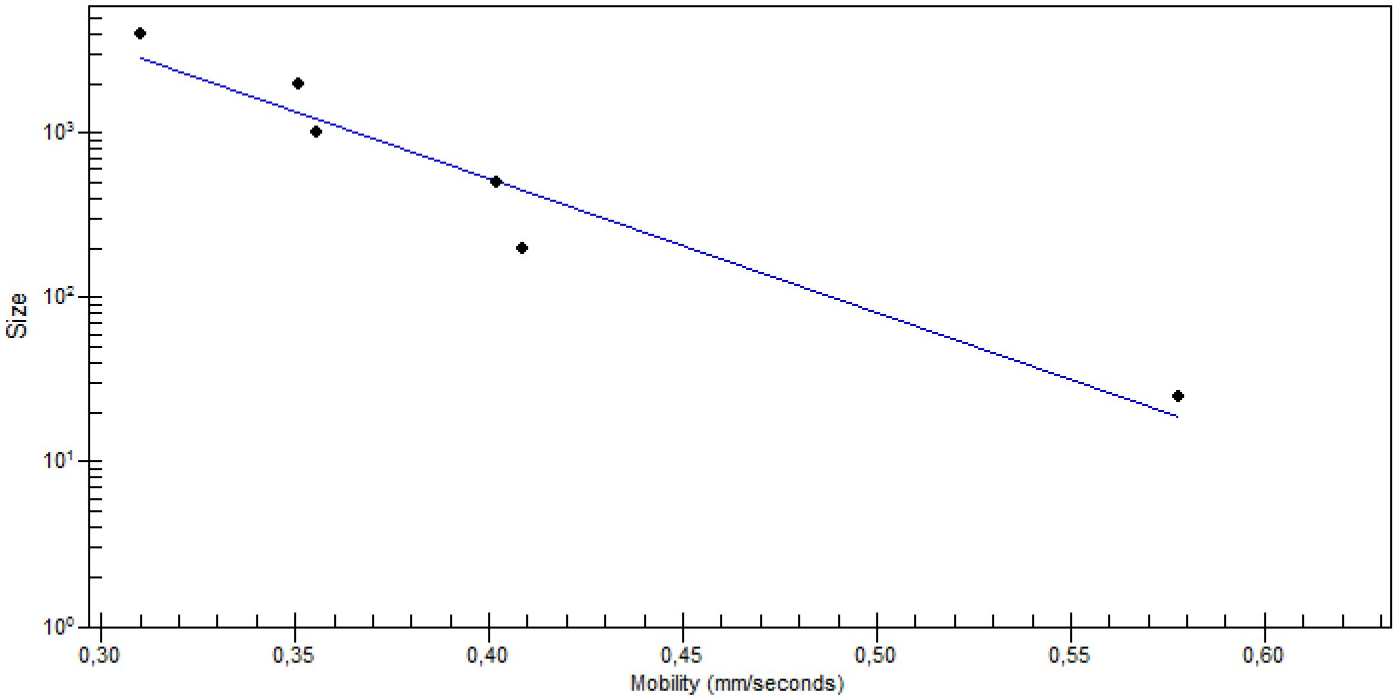

 © Copyright 2003 - 2009 Agilent Technologies, Inc.

Assay Class: Eukaryote Total RNA Nano
Data Path: C:\...Eukaryote Total RNA Nano_DE13806198_2016-05-31_15-33-49.xad
Created: 31.05.2016 15:33:48
Modified: 31.05.2016 15:57:45

Run Logbook

Description	Number	Source	Category	Sub Category	Time	Time Zone	User	Host
Run ended on port 1 (Number of wells acquired: 13)		Instrument	Run		31.05.2016 15:57:41	(GMT +02:00) Romance Standard Time	cary100	cary100-HP
Run started on port 1 (File: C:\Program Files\Agilent\2100 bioanalyzer\2100 expert\Data\2016-05-31\2100 expert_Eukaryote Total RNA Nano_DE13806198_2016-05-31_15-33-49.xad)		Instrument	Run		31.05.2016 15:33:54	(GMT +02:00) Romance Standard Time	cary100	cary100-HP
Product Number : G2939A		Instrument	Run		31.05.2016 15:33:54	(GMT +02:00) Romance Standard Time	cary100	cary100-HP
Name :		Instrument	Run		31.05.2016 15:33:54	(GMT +02:00) Romance Standard Time	cary100	cary100-HP
Vendor : Agilent Technologies		Instrument	Run		31.05.2016 15:33:54	(GMT +02:00) Romance Standard Time	cary100	cary100-HP
Serial# : DE13806198		Instrument	Run		31.05.2016 15:33:54	(GMT +02:00) Romance Standard Time	cary100	cary100-HP
Firmware : C.01.069		Instrument	Run		31.05.2016 15:33:54	(GMT +02:00) Romance Standard Time	cary100	cary100-HP
Cartridge : Electrode		Instrument	Run		31.05.2016 15:33:54	(GMT +02:00) Romance Standard Time	cary100	cary100-HP

© Copyright 2003 - 2009 Agilent Technologies, Inc.
